# Selective inhibition of the kinase DYRK1A by targeting its folding process

**DOI:** 10.1038/ncomms11391

**Published:** 2016-04-22

**Authors:** Isao Kii, Yuto Sumida, Toshiyasu Goto, Rie Sonamoto, Yukiko Okuno, Suguru Yoshida, Tomoe Kato-Sumida, Yuka Koike, Minako Abe, Yosuke Nonaka, Teikichi Ikura, Nobutoshi Ito, Hiroshi Shibuya, Takamitsu Hosoya, Masatoshi Hagiwara

**Affiliations:** 1 Department of Anatomy and Developmental Biology, Graduate School of Medicine, Kyoto University, Yoshida-Konoe-cho, Kyoto 606-8501, Japan; 2 Pathophysiological and Health Science Team, Imaging Application Group, Division of Bio-Function Dynamics Imaging, RIKEN Center for Life Science Technologies, 6-7-3 Minatojima-minamimachi, Chuo-ku, Kobe 650-0047, Japan; 3 Laboratory of Chemical Bioscience, Institute of Biomaterials and Bioengineering, Tokyo Medical and Dental University, 2-3-10 Kanda-Surugadai, Tokyo 101-0062, Japan; 4 Department of Molecular Cell Biology, Medical Research Institute, Tokyo Medical and Dental University, 1-5-45, Yushima, Tokyo 113-8510, Japan; 5 Department of Structural Biology, Graduate School of Medical and Dental Sciences, Medical Research Institute, Tokyo Medical and Dental University, 1-5-45, Yushima, Tokyo 113-8510, Japan

## Abstract

Autophosphorylation of amino-acid residues is part of the folding process of various protein kinases. Conventional chemical screening of mature kinases has missed inhibitors that selectively interfere with the folding process. Here we report a cell-based assay that evaluates inhibition of a kinase at a transitional state during the folding process and identify a folding intermediate-selective inhibitor of dual-specificity tyrosine-phosphorylation-regulated kinase 1A (DYRK1A), which we refer to as FINDY. FINDY suppresses intramolecular autophosphorylation of Ser97 in DYRK1A in cultured cells, leading to its degradation, but does not inhibit substrate phosphorylation catalysed by the mature kinase. FINDY also suppresses Ser97 autophosphorylation of recombinant DYRK1A, suggesting direct inhibition, and shows high selectivity for DYRK1A over other DYRK family members. In addition, FINDY rescues DYRK1A-induced developmental malformations in *Xenopus laevis* embryos. Our study demonstrates that transitional folding intermediates of protein kinases can be targeted by small molecules, and paves the way for developing novel types of kinase inhibitors.

## Abstract (web summary)

Kinase inhibitors are important drugs and usually target the ATP binding pocket of kinases. Here, Kii *et al.* report a completely new type of kinase inhibitor that specifically targets the protein folding intermediate state, but not the mature form, of the protein kinase DYRK1A.

## Graphical abstract

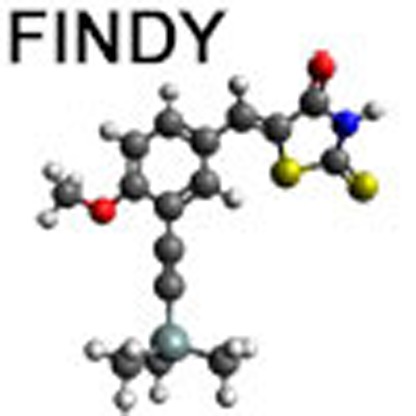

Dysregulation of protein kinase activity has been implicated in pathological conditions, such as neurological disorders and tumorigenesis. The protein kinase family represents an attractive target for drug development^1^,^2^. Kinase inhibitors that target ATP-binding pockets sometimes cause adverse side effects by suppressing unintended kinases, because the sequence and structure of the pockets are well-conserved^3^. Innovative ideas are therefore necessary in drug discovery to obtain a highly selective inhibitor of the target kinase.

*DYRK1A*, dual-specificity tyrosine-phosphorylation-regulated kinase 1A, is a mammalian orthologue of *Drosophila minibrain* and is essential for brain development^4^. The physiological importance of *DYRK1A* has been suggested by its proposed relationship with various symptoms of Down syndrome (DS)^5^,^6^,^7^. *DYRK1A* resides within the obligate trisomic region of human chromosome 21 and the extra copy of the *DYRK1A* gene in people with DS causes a 1.5-fold increased expression of the dual-specificity tyrosine-phosphorylation-regulated kinase 1A (DYRK1A) protein^8^. The excessive DYRK1A activity is not only pivotal in causing the characteristic facial features^9^,^10^ and congenital heart defects^9^ of DS, but is also associated with early-onset of Alzheimer's disease^11^,^12^. The hypothesis that the elevated activity of DYRK1A contributes to these neurological disorders has stimulated an interest in DYRK1A as a potential target for therapeutic drugs^4^,^12^,^13^. In addition, inhibition of DYRK1A increases pancreatic β-cell proliferation, suggesting therapeutic promise for diabetes therapy^14^,^15^. To repress the excessive activity of DYRK1A, we had previously developed a synthetic small molecule, INDY, which potently suppresses the kinase activity of DYRK1A. INDY also inhibits other DYRK family members as well as Cdc2-like kinases^16^.

DYRKs and glycogen synthase kinase 3β (GSK3β) autophosphorylate their own tyrosine residue in their transitional state and phosphorylate serine or threonine residues on their substrates after maturation^17^,^18^,^19^,^20^,^21^. Lochhead *et al.* postulated the existence of a transitional intermediate of these kinases that has biochemical properties distinct from the mature state^18^,^19^. Interestingly, the intermediate showed different sensitivity to chemical inhibitors from the mature kinase. For example, the transitional intermediate of *Drosophila* DYRK2 was inhibited by Purvalanol A, but not by 4,5,6,7-tetrabromo-1H-benzotriazole (TBB), whereas the mature kinase was inhibited by both^18^. So far, selective inhibitors of the transitional intermediate have not yet been identified, because the focus of conventional drug screening has been on the mature forms of the kinases^18^,^22^.

Isolation of transitional intermediates is considered to be quite difficult because of their low population and short lifetimes^23^. Therefore, we developed a cell-based assay, named SPHINKS (substrate phosphorylation by sequential induction of kinase and substrate), to evaluate intermediate-selective inhibitors of DYRK1A. Through examination of our synthetic chemical library, we identified a small compound, referred to as FINDY (folding intermediate-selective inhibitor of DYRK1A), which interfered with the folding process of DYRK1A, but did not inhibit the substrate phosphorylation catalysed by the mature kinase. FINDY specifically suppressed autophosphorylation of Ser97 in DYRK1A, resulting in its degradation by proteasomes. Furthermore, we found that FINDY did not affect the kinase activity of the other members of the DYRK family, indicating the possibility that transitional intermediate-selective kinase inhibitors distinguish between the conserved family members.

## Results

### Design of the cell-based assay targeting the intermediate

**Figure 1:**
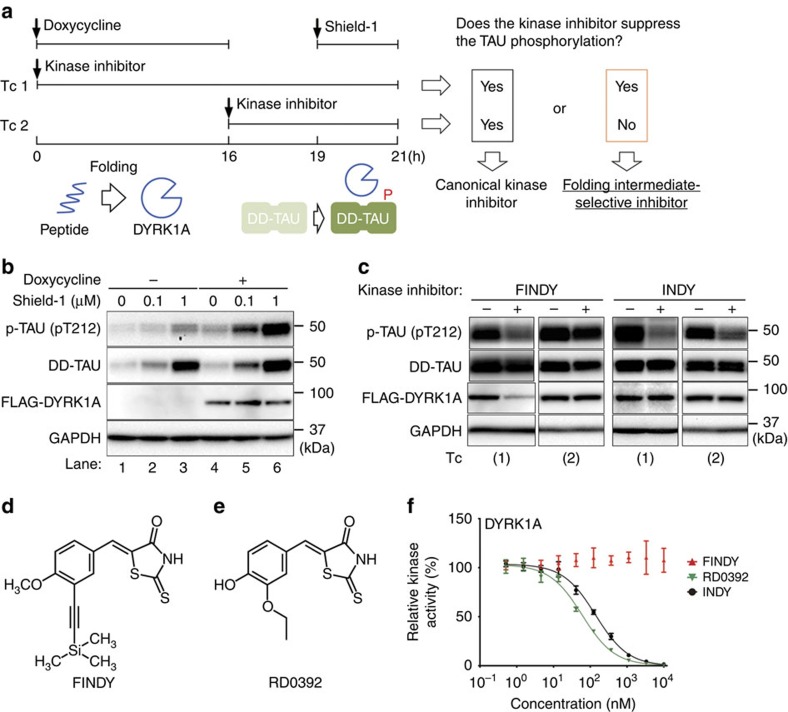
Cell-based assay to evaluate a transitional intermediate-selective inhibitor of DYRK1A. (**a**) Schematic diagram of the SPHINKS assay. Doxycycline induces DYRK1A expression. Subsequently, Shield-1 stabilizes TAU fused with the destabilization domain of FKBP12 (DD-TAU). DYRK1A phosphorylates DD-TAU over the defined time period (19–21 h). Small molecules from our chemical library were added at the indicated points in Tc 1 and 2. Canonical kinase inhibitors suppress TAU phosphorylation in both Tc 1 and 2. Intermediate-selective inhibitors of DYRK1A should suppress TAU phosphorylation in Tc 1, but not in Tc 2. (**b**) Inducible expression of FLAG-DYRK1A and DD-TAU in HEK293 cells. Doxycycline induced FLAG-DYRK1A expression (lanes 4–6) and Shield-1 stabilized DD-TAU (lanes 2, 3, 5, 6). FLAG-DYRK1A predominantly phosphorylated Thr212 of TAU (p-TAU; lanes 5 and 6). p-TAU, TAU, FLAG and GAPDH were detected by western blot, using their corresponding antibodies. Representative data from the triplicate experiments are shown. (**c**) Identification of FINDY as the intermediate-selective inhibitor of DYRK1A. TAU phosphorylation was suppressed by FINDY (10 μM) in Tc 1, but not in Tc 2. In contrast, the canonical DYRK1A inhibitor INDY (10 μM) suppressed TAU phosphorylation in both Tc 1 and 2. Representative data from the triplicate experiments are shown. (**d**) Structure of FINDY. (**e**) Structure of RD0392, a canonical ATP-competitive inhibitor of DYRK1A. (**f**) FINDY did not inhibit the *in vitro* kinase activity of DYRK1A. Recombinant DYRK1A was incubated with the peptide substrate DYRKtide in the presence of FINDY, RD0392 or INDY. RD0392 and INDY inhibited the kinase activity with IC_50_ values of 60.2 and 139 nM, respectively. Representative dose-response curves with Hill slopes are shown. The results are presented as means±s.d. (*n*=4).

To evaluate intermediate-selective inhibitors of DYRK1A, we developed the SPHINKS assay, enabling us to evaluate kinase inhibition at the transitional state during the folding process (Fig. 1a). We first established a HEK293 cell line with dual-inducible expression of DYRK1A and TAU, a well-characterized substrate of DYRK1A^24^,^25^. Expression of FLAG-tagged DYRK1A (FLAG-DYRK1A) was controlled by the *tet* operator, and was initiated by treatment with doxycycline (Fig. 1b, lanes 4–6). TAU was expressed in a fused form with the destabilization domain FKBP12 (DD), which causes degradation of DD fusion proteins in the absence of a small molecule, Shield-1 (ref. 26). Treatment with Shield-1 for 2 h stabilized DD-TAU in a dose-dependent manner (Fig. 1b, lanes 2, 3, 5 and 6). Phosphorylation of the stabilized DD-TAU at Thr212 was enhanced 4 h after the administration of doxycycline (Fig. 1b, lanes 5 and 6; and Supplementary Fig. 1), indicating that DYRK1A is produced from the doxycycline-induced transcript within 4 h.

To distinguish kinase inhibition at the transitional state from that after maturation, we set up two types of time courses. Small molecules were administered either before the induction by doxycycline (time course: Tc 1) or after the accumulation of mature DYRK1A (Tc 2; Fig. 1a). Following additional incubation with Shield-1 for 2 h, total cell lysates were harvested, then subjected to western blot. If the compound selectively inhibits the transitional intermediate, but not the mature DYRK1A, it should suppress TAU phosphorylation in Tc 1, but not in Tc 2, as illustrated in Fig. 1a.

### Identification of FINDY

Using the SPHINKS assay, we examined our chemical library and found a small molecule that suppressed TAU phosphorylation in Tc 1, but not in Tc 2 (Fig. 1c). This small molecule was referred to as FINDY (Fig. 1d). In contrast, the canonical DYRK1A inhibitor INDY suppressed TAU phosphorylation in both Tc 1 and 2 (Fig. 1c).

FINDY is structurally similar to an ATP-competitive inhibitor, RD0392 (Fig. 1e and Supplementary Fig. 2), which we identified through *in vitro* screening using recombinant DYRK1A. RD0392 bears a five-membered heterocyclic ring, rhodanine. This moiety has been reported as a substructure of Pan Assay Interference Compounds^27^, which are compounds that have frequently arisen as hits in high-throughput screening. Conversely, Mendgen *et al.* noted that small molecules bearing rhodanine are potentially valuable and should not be regarded as problematic or promiscuous binders *per se*^28^. Therefore, we synthesized structural derivatives of RD0392, one of which was FINDY.

In contrast to the dose-dependent inhibition by RD0392 and INDY in the *in vitro* assay, FINDY did not exert any observable inhibitory effect on the recombinant DYRK1A (Fig. 1f). This was supported by a computational docking simulation study, which showed that RD0392 was able to fit to the ATP-binding pocket of mature DYRK1A (Supplementary Fig. 3a,b), whereas FINDY could not (Supplementary Fig. 3c,d). FINDY decreased the amount of FLAG-DYRK1A in Tc 1, whereas DD-TAU and glyceraldehyde 3-phosphate dehydrogenase (GAPDH) were unaffected. In contrast, the amount of FLAG-DYRK1A was unaltered in Tc 2 (Fig. 1c). These results suggest that FINDY selectively impairs the stability or translation of DYRK1A, leading to the suppression of TAU phosphorylation in Tc 1.

**Figure 2:**
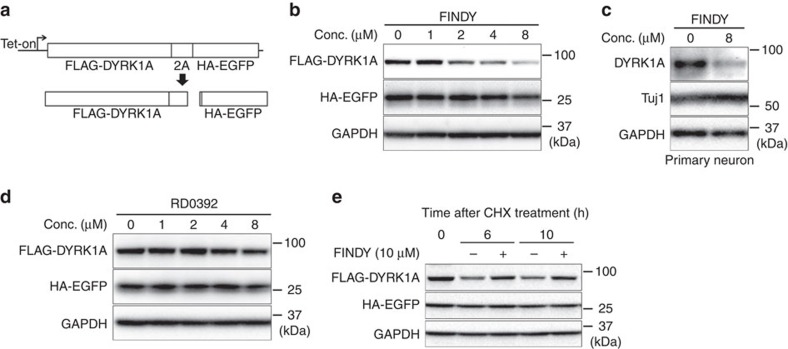
FINDY destabilizes DYRK1A during the folding process. (**a**) Schematic diagram of the 2A peptide-mediated bicistronic expression of FLAG-DYRK1A and HA-EGFP. Expression is controlled by the *tet* operator. Effects of FINDY (**b**) or RD0392 (**d**) on destabilization of DYRK1A. FINDY (**b**) or RD0392 (**e**) was added with doxycycline to HEK293 cells harbouring the bicistronic expression vector, which was stably integrated in the genome. After incubation for 5 h, total cell lysates were subjected to SDS–PAGE followed by western blot analysis using the corresponding antibodies against FLAG, HA and GAPDH. Representative data from the pentaplicate experiments are shown. (**c**) The amount of endogenous DYRK1A was decreased by FINDY. Primary cortical neurons were cultured in the presence of FINDY for 3 days. DYRK1A, Tuj1 (neuron-specific class III β-tubulin) and GAPDH were detected by western blot analysis using their corresponding antibodies. Representative data from the triplicate experiments are shown. (**e**) FINDY did not induce the degradation of mature DYRK1A. The cells were treated with doxycycline for 16 h, then incubated with cycloheximide (CHX) with or without FINDY for the indicated time. Total cell lysates were subjected to SDS–PAGE followed by western blot analysis. Representative data from the triplicate experiments are shown. Conc., concentration.

To determine whether the stability or the translation is affected by FINDY, we constructed a translation reporter system using ribosome skipping mediated by a 2A peptide. The 2A peptide inhibits normal peptide bond formation without affecting translation^29^, which enables the bicistronic expression of FLAG-DYRK1A and haemagglutinin (HA)-tagged enhanced green fluorescent protein (EGFP) (HA-EGFP) under the control of a doxycycline-driven promoter (Fig. 2a). When administered with doxycycline, FINDY decreased the amount of FLAG-DYRK1A in a dose-dependent manner (EC_50_ value: 2.2 μM), without affecting HA-EGFP (Fig. 2b). FINDY also decreased the amount of endogenous DYRK1A in a primary culture of cortical neurons (Fig. 2c). On the other hand, administration of RD0392 did not decrease the amount of FLAG-DYRK1A (Fig. 2d). Next, we examined the effect of FINDY on the degradation of FLAG-DYRK1A in the presence of a translation inhibitor, cycloheximide. Cells were pre-treated with doxycycline for 16 h, then incubated with cycloheximide and FINDY. Under this condition, FINDY did not induce the degradation of FLAG-DYRK1A compared with the vehicle treatment (Fig. 2e), indicating that FINDY destabilizes newly translated FLAG-DYRK1A.

### FINDY suppresses autophosphorylation on Ser97 of DYRK1A

**Figure 3:**
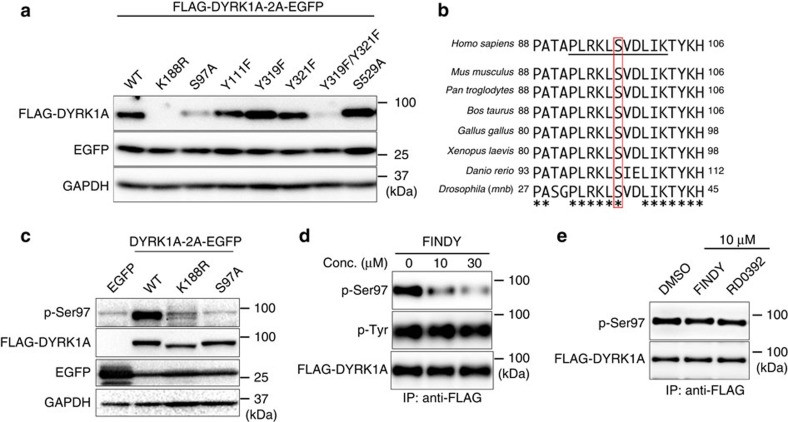
FINDY suppresses autophosphorylation of Ser97 in DYRK1A. (**a**) The bicistronic expression of intact DYRK1A (WT) and the substituted mutants of Lys188 to Arg (K188R), Ser97 to Ala (S97A), Tyr111 to Phe (Y111F), Tyr319 to Phe (Y319F), Tyr321 to Phe (Y321F), Tyr319 and Tyr321 to Phe (Y319F/Y321F) and Ser529 to Ala (S529A) with EGFP in HEK293 cells. The expression vectors were stably integrated in the genome of the cells. Each protein was detected with the corresponding antibody against FLAG, EGFP or GAPDH. Representative data from the quadruplicate experiments are shown. (**b**) The alignment of Ser97 and its surrounding amino-acid sequences from *DYRK1A* of the indicated species. The conserved serine residues corresponding to Ser97 of *Homo sapiens DYRK1A* are highlighted by the red rectangular box. Underlined portions indicate the phospho-peptide used in the immunization. (**c**) The polyclonal antibody against phosphorylated Ser97 (p-Ser97) specifically detected FLAG-DYRK1A (WT), but not the kinase-dead (K188R) or S97A mutants. The cells were transiently transfected with the expression vectors of the indicated constructs and the proteins were overexpressed. Total cell lysates were subjected to SDS–PAGE followed by western blot analysis. Representative data from the triplicate experiments are shown. (**d**) FINDY suppressed Ser97 autophosphorylation. FLAG-DYRK1A was produced in HEK293 cells incubated with epoxomicin and FINDY for 5 h, then purified with anti-FLAG antibody-conjugated beads. Each protein was detected with the corresponding antibodies against p-Ser97, phospho-Tyr (p-Tyr) or FLAG. Representative data from the triplicate experiments are shown. (**e**) FINDY did not suppress Ser97 autophosphorylation in Tc 2. FLAG-DYRK1A was produced in HEK293 cells for 16 h in the presence of doxycycline, after which cells were treated with 10 μM of FINDY or RD0392 for 5 h. Total cell lysates were subjected to immunoprecipitation (IP) with anti-FLAG antibody-conjugated beads. Each protein was detected with the corresponding antibodies against p-Ser97 and FLAG. Representative data from the duplicate experiments are shown. Conc., concentration.

Catalytic activity of several protein kinases is regulated by autophosphorylation to stabilize the active conformation^30^. To investigate whether autophosphorylation is involved in the stabilization of DYRK1A, we prepared a substituted mutant with Arg (K188R) in place of Lys188. Lys188 in DYRK1A interacts directly with ATP; the substitution thus renders the kinase inactive, leading to a ‘kinase-dead' mutant, which lacks autophosphorylation ability^31^. In our study, the K188R mutant protein of FLAG-DYRK1A was barely detected with western blot, whereas EGFP from the bicistronic cassette was detected (Fig. 3a, K188R). This suggests that the abrogation of autophosphorylation destabilizes the DYRK1A protein. To identify the residues required for the stabilization of DYRK1A, we mutated potential autophosphorylation sites. Ser97 and Tyr111 in the non-catalytic amino-terminal domain of DYRK1A can be autophosphorylated^31^. In addition, Tyr319 or Tyr321 in the activation loop are required to retain catalytic activity^31^,^32^,^33^, and the autophosphorylation of these residues has also been reported^21^. Similarly, the autophosphorylation of Ser520, which corresponds to Ser529 in the DYRK1A variant used in the present study, is required for its binding to 14-3-3β (ref. 34). Therefore, we prepared mutants with these residues substituted. The substituted mutant of S97A and the double mutant of Y321F/Y319F were degraded, whereas the other mutants remained (Fig. 3a), indicating that Ser97 and one of the tyrosine residues in the activation loop are required for the DYRK1A stabilization.

Ser97 and its surrounding amino-acid sequence in DYRK1A are highly conserved among various species (Fig. 3b), suggesting their functional importance. To detect phosphorylated Ser97 (p-Ser97), we generated a polyclonal antibody against p-Ser97, and transiently transfected 293T cells with the expression vectors of FLAG-DYRK1A and its mutants, in which the mutants were detectable, probably because the overexpressed proteins were beyond the capacity of proteasomal degradation. This antibody detected the intact DYRK1A (wild type, WT), but barely identified the kinase-dead (K188R) and S97A mutants in the western blot analysis (Fig. 3c). In addition, competition experiments using phospho- and non-phospho-peptides clearly confirmed the antibody's specific recognition ability towards p-Ser97 (Supplementary Fig. 4). We then examined whether FINDY suppresses autophosphorylation. In the presence of FINDY, the amount of p-Ser97 significantly decreased, whereas signals for phosphorylated tyrosine residues (p-Tyr) were unaffected (Fig. 3d). In addition, mass spectrometry analysis of the trypsin-digested peptides of FLAG-DYRK1A showed that FINDY did not suppress the phosphorylation of Tyr321 in the activation loop (Supplementary Fig. 5). To confirm that the FINDY-mediated inhibition of Ser97 autophosphorylation is involved in degradation of DYRK1A, we examined whether FINDY destabilized the DYRK1A mutants of K188R and S97A, which were not able to catalyse Ser97 autophosphorylation. FINDY did not decrease the level of these mutants (Supplementary Fig. 6). Thus, FINDY acts as an inhibitor of the autophosphorylation and does not induce DYRK1A degradation by an independent mechanism. These results thus suggest that FINDY destabilizes DYRK1A by inhibiting Ser97 autophosphorylation.

To further determine whether Ser97 is autophosphorylated by an inter- or intramolecular mechanism, we generated HEK293 cells that co-expressed the kinase-dead K188R mutant (FLAG-DYRK1A K188R) and an intact DYRK1A fused with a DD tag (DD-FLAG-DYRK1A). If Ser97 autophosphorylation were mediated by an intermolecular mechanism, Ser97 of the kinase-dead K188R mutant would be phosphorylated when expressed in the presence of DD-FLAG-DYRK1A. However, we found that the overexpressed DD-FLAG-DYRK1A did not enhance the phosphorylation of Ser97 in the K188R mutant (Supplementary Fig. 7a). In addition, DD-FLAG-DYRK1A did not stabilize the co-expressed K188R protein (Supplementary Fig. 7b). These results suggest that the Ser97 autophosphorylation of DYRK1A is catalysed in an intramolecular manner. Furthermore, treatment with cantharidic acid, a protein phosphatase inhibitor, increased the amount of p-Ser97 signal in FLAG-DYRK1A (Supplementary Fig. 7c), suggesting that p-Ser97 is dephosphorylated by a phosphatase inhibited by cantharidic acid. Interestingly, the FLAG-DYRK1A protein, which was purified from HEK293 cells, did not significantly catalyse Ser97 autophosphorylation (Supplementary Fig. 7d,e). Consistently, FINDY did not decrease the levels of p-Ser97 in HEK293 cells when cells were treated with FINDY after the accumulation of mature DYRK1A (Fig. 3e). Taken together, these results suggest that Ser97 autophosphorylation is catalysed before maturation and that p-Ser97 is susceptible to dephosphorylation after maturation.

### FINDY inhibits incorporation of biotin-ATP

**Figure 4:**
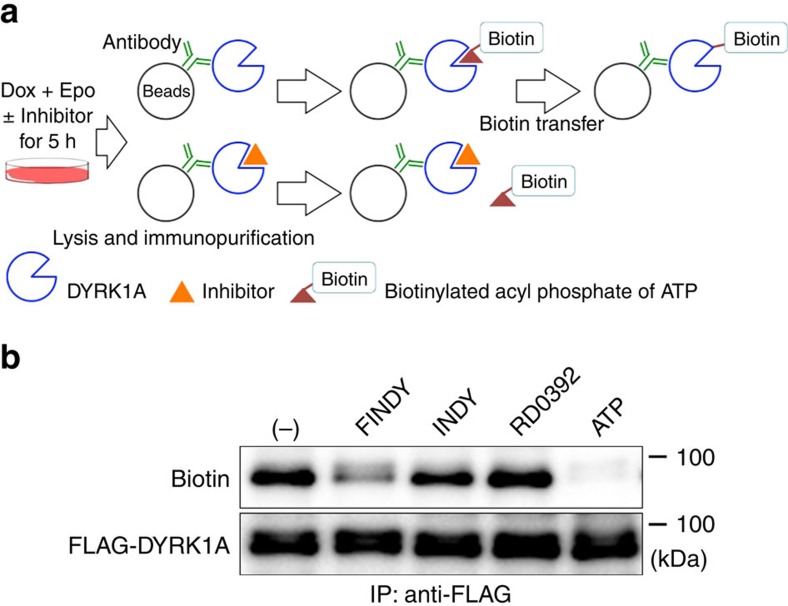
FINDY affects the folding of DYRK1A, targeting the ATP-binding pocket. (**a**) Schematic diagram of the biotin-transfer experiment. Expression of FLAG-DYRK1A is induced by doxycycline (Dox) with epoxomicin (Epo) and kinase inhibitors (10 μM). The resulting FLAG-DYRK1A proteins are immunopurified and reacted with the biotinylated acyl phosphate of ATP (20 μM). If the inhibitor occupies the ATP-binding pocket, then biotin transfer is prevented. (**b**) FINDY competes with the biotin-ATP analogue for the DYRK1A intermediate. FINDY, INDY and RD0392 were used as described in **a**. As a positive control for the competition experiment, the FLAG-DYRK1A protein was pre-treated with ATP (100 μM) before the reaction with the biotin-ATP analogue. The labelled proteins were blotted with streptavidin and anti-FLAG antibody. Representative data from the triplicate experiments are shown. IP, immunoprecipitation.

We examined whether FINDY inhibited incorporation of ATP into the pocket of DYRK1A. An ATP analogue, the biotinylated acyl phosphate of ATP, which fits to the ATP-binding pocket of kinases, irreversibly reacts with the ATP-orienting lysine and transfers biotin to this lysine^35^. We applied this method in designing an experiment, which is illustrated in Fig. 4a. If FINDY occupies the ATP-binding pocket of the purified kinase, it would inhibit the binding of the biotin-ATP analogue to this region. FLAG-DYRK1A produced in the presence or absence of the small molecules was immunopurified from the cells. The biotinylation of the immunopurified FLAG-DYRK1A produced in the presence of FINDY was significantly suppressed compared with the vehicle control, whereas that of FLAG-DYRK1A produced with either INDY or RD0392 was not (Fig. 4b). This indicates that FINDY inhibits the incorporation of biotin-ATP into the DYRK1A intermediate during the folding process. As a positive control, treatment of the immunopurified FLAG-DYRK1A with 100 μM ATP before incubation with the biotin-ATP analogue blocked biotinylation (Fig. 4b).

### Ser97 autophosphorylation in *in vitro* translation

**Figure 5:**
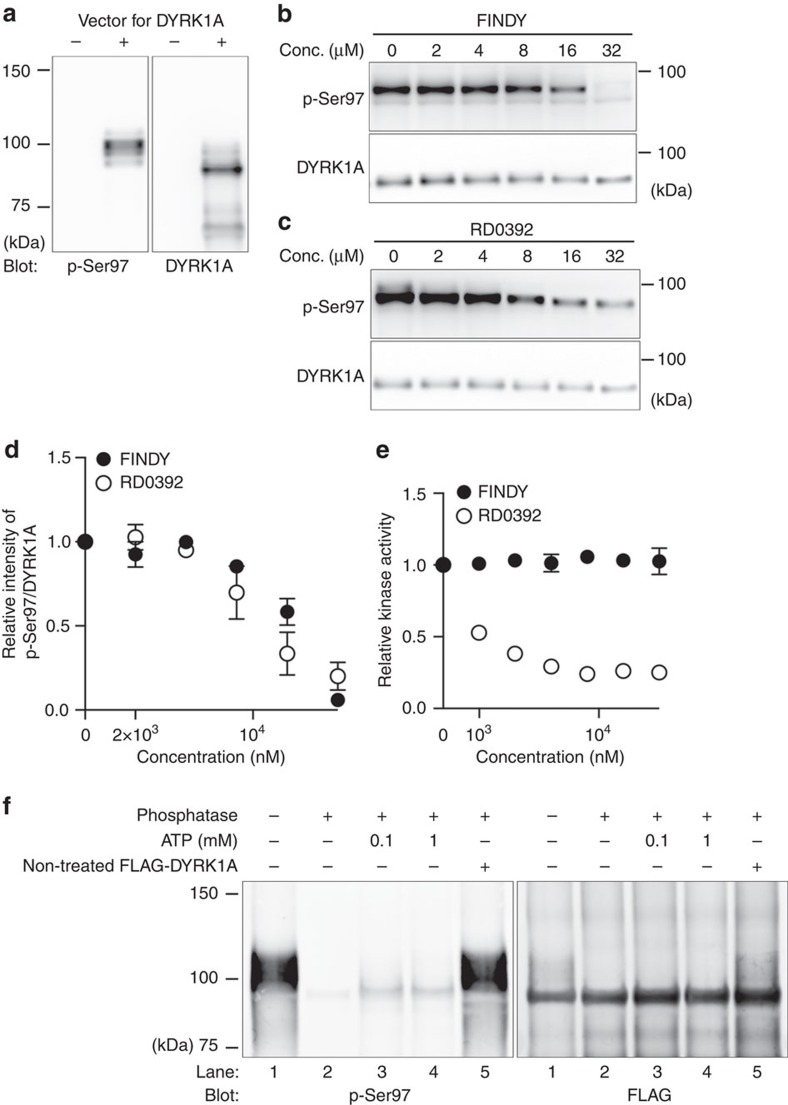
FINDY suppresses Ser97 autophosphorylation of DYRK1A in an *in vitro* transcription-translation system. (**a**) Expression of DYRK1A in a cell-free *E. coli*-based coupled transcription-translation system. Representative data from the duplicate experiments are shown. (**b**,**c**) *In vitro* expression of DYRK1A in the presence of FINDY (**b**) or RD0392 (**c**). Representative data from the triplicate experiments are shown. (**d**) Relative activities of Ser97 autophosphorylation in the *in vitro* expression of DYRK1A in the presence of FINDY or RD0392. The band intensities in **b**,**c** were quantified. The graph shows the means±s.d. (*n*=3). (**e**) DYRK1A produced in the *in vitro* system was incubated with the peptide substrate DYRKtide in the presence of FINDY or RD0392 (up to 32 μM). The results are presented as means±s.d. (*n*=3). (**f**) DYRK1A produced in the *in vitro* system was purified with anti-FLAG antibody-conjugated beads, then reacted with lambda protein phosphatase for 2 h. After washout of phosphatase, the dephosphorylated DYRK1A was allowed to autophosphorylate in the presence of ATP and sodium orthovanadate (1 mM) for 2 h. To check the residual phosphatase activity, the non-treated FLAG-DYRK1A protein was added into the autophosphorylation reaction (lane 5). Representative data from the duplicate experiments are shown. Conc., concentration.

To confirm the effect of FINDY on Ser97 autophosphorylation of DYRK1A in a simpler assay than living cells, we employed an *Escherichia coli*-based coupled *in vitro* transcription-translation system. The PURExpress system is reconstituted from recombinant proteins and purified ribosomes. The signal of p-Ser97 of DYRK1A was detected when expressed in the PURExpress system (Fig. 5a), indicating a possibility that Ser97 autophosphorylation does not require mammalian proteins. The band of p-Ser97 migrated slower than the major band of the FLAG-DYRK1A protein (Fig. 5a), indicating that a small portion of DYRK1A is autophosphorylated in this experiment. This suggests a low probability of the Ser97 autophosphorylation in the *in vitro* translation. We then performed the *in vitro* translation in the presence of FINDY or RD0392. FINDY and RD0392 suppressed Ser97 autophosphorylation in similar dosage ranges (Fig. 5b–d). In addition, FINDY did not inhibit substrate phosphorylation by the DYRK1A protein produced in the PURExpress system (Fig. 5e). In contrast, RD0392 inhibited substrate phosphorylation in a dose-dependent manner (Fig. 5e). We also examined tyrosine autophosphorylation of DYRK1A. In the PURExpress system, no significant effects of FINDY or RD0392 were observed on the signal from phosphorylated tyrosine (Supplementary Fig. 8). These results indicate that FINDY suppresses Ser97 autophosphorylation during the *in vitro* expression of DYRK1A.

We also confirmed whether the mature DYRK1A catalysed Ser97 autophosphorylation or not using the FLAG-DYRK1A protein produced in the PURExpress system. Immunopurified FLAG-DYRK1A bound on resin was reacted with lambda protein phosphatase, then allowed to autophosphorylate in the presence of ATP for 2 h. Consistent with the results using FLAG-DYRK1A purified from HEK293 cells (Supplementary Fig. 7d,e), the purified DYRK1A protein did not catalyse Ser97 autophosphorylation (Fig. 5f). Thus, Ser97 autophosphorylation is a one-off event during the translation or folding process. These results demonstrate that FINDY suppressed Ser97 autophosphorylation before the maturation of DYRK1A.

### Ser97 autophosphorylation of recombinant DYRK1A proteins

**Figure 6:**
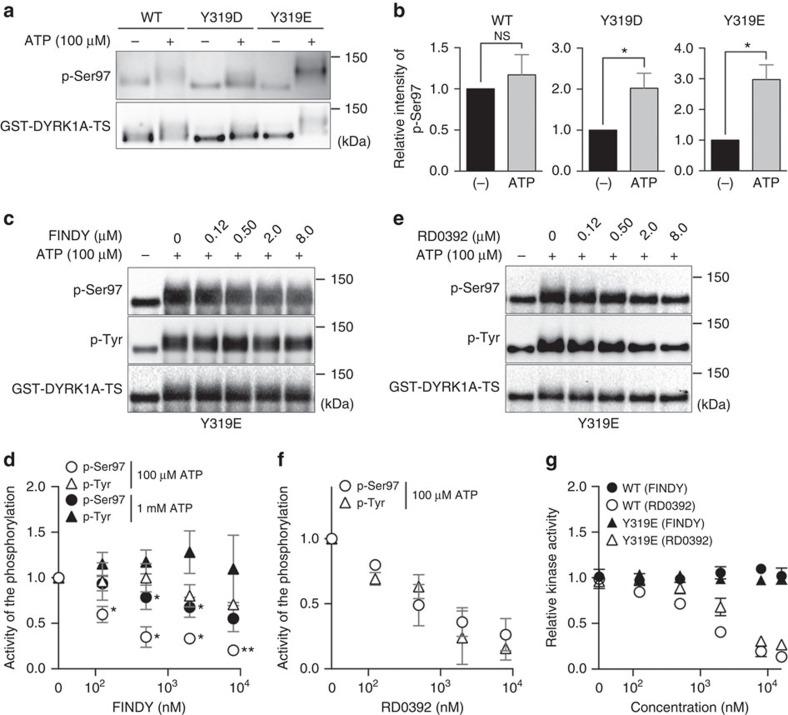
FINDY inhibits Ser97 autophosphorylation of recombinant DYRK1A protein. (**a**) Recombinant GST-DYRK1A-TS protein (WT) and its pseudo-phosphorylation mutants (Y319D and Y319E), produced at 6 °C in *E. coli*, were reacted with ATP (100 μM), then analysed by SDS–PAGE followed by western blot analysis. Representative data from the quadruplicate experiments are shown. (**b**) The band intensities in **a** were quantified. The graph shows the means±s.d. (*n*=4). Statistical significance was calculated compared with the value without ATP. NS indicates not significant. *P<0.05. Statistical analysis was performed with the Mann–Whitney *U*-test. (**c**) The Y319E mutant was reacted with ATP (100 μM) in the presence of FINDY. Representative data from the triplicate experiments are shown. (**d**) Relative activities of Ser97 and tyrosine phosphorylation in the presence of ATP (100 μM and 1 mM) and FINDY, which were calculated from the ATP-dependent increase in the intensities of p-Ser97 and p-Tyr (see the Methods for details). The graph represents means±s.d. (*n*=3). Statistical significance was calculated compared with the value of the relative activity of p-Tyr at the same concentration of FINDY. *P<0.05 and **P<0.01. Data were analysed with the paired *t*-test. (**e**) RD0392 inhibited the phosphorylation of Ser97 and tyrosine residues. The Y319E mutant was reacted with ATP (100 μM) in the presence of RD0392. Representative data from the triplicate experiments are shown. (**f**) Relative activities of Ser97 and tyrosine phosphorylation in the presence of ATP (100 μM) and RD0392, which were calculated from the ATP-dependent increase in the intensities of p-Ser97 and p-Tyr (see the Methods for details). The graph represents means±s.d. (*n*=3). (**g**) The recombinant DYRK1A proteins were incubated with the peptide substrate DYRKtide in the presence of FINDY or RD0392 (up to 16 μM). The results are presented as means±s.d. (*n*=3).

To determine whether FINDY directly inhibits Ser97 autophosphorylation, we developed an *in vitro* autophosphorylation assay using a recombinant DYRK1A protein. DYRK1A fused with a GST-tag at the amino-terminus and with a Twin-Strep-tag (TS) at the carboxy-terminus (GST-DYRK1A-TS) was produced in *E. coli* cells at 6 °C to suppress autophosphorylation, as we found that the p-Ser97 signal decreased in cells cultured at low temperature (Supplementary Fig. 9a). GST-DYRK1A-TS was purified using tandem affinity purification with *Strep*-Tactin Sepharose followed by GSH-conjugating resin (Supplementary Fig. 9b), then subjected to an *in vitro* reaction with ATP (100 μM). Under these conditions, the p-Ser97 signal did not increase, whereas the protein band was broadly shifted (Fig. 6a,b). Therefore, we wondered if the incubation at 6 °C suppressed prior autophosphorylation of the tyrosine residues (Tyr319 and Tyr321) in the activation loop, which are required for Ser97 autophosphorylation (Supplementary Fig. 9c). To verify this possibility, we generated pseudo-phosphorylation mutants, in which these tyrosine residues were substituted for phosphomimetic aspartic or glutamic acid residues (Y319D, Y319E, Y321D and Y321E), then examined their facilitation of Ser97 autophosphorylation. The Y319D and Y319E mutants were purified (Supplementary Fig. 9d), whereas the Y321D and Y321E mutants formed aggregates. As expected, the Y319D and Y319E mutations facilitated Ser97 autophosphorylation of recombinant GST-DYRK1A-TS (Fig. 6a,b). Furthermore, the Y319E mutant catalysed substrate phosphorylation at an equivalent level to the WT kinase (Supplementary Fig. 9e).

We then examined the effect of FINDY on *in vitro* Ser97 autophosphorylation using the Y319E mutant. FINDY inhibited Ser97 autophosphorylation with an IC_50_ value of 110 nM, but did not suppress tyrosine phosphorylation (Fig. 6c,d), which is consistent with results in Fig. 3d. The FINDY-mediated inhibition of Ser97 autophosphorylation decreased due to competition with excess ATP (1 mM; Fig. 6d and Supplementary Fig. 9e). In contrast, the canonical inhibitor RD0392 suppressed both Ser97 and tyrosine phosphorylation in the Y319E mutant (Fig. 6e,f), with IC_50_ values of 205 and 514 nM, respectively. These results demonstrate that FINDY directly and selectively inhibits Ser97 autophosphorylation.

In addition, we examined whether or not FINDY inhibited substrate phosphorylation by these recombinant proteins. The *in vitro* kinase assay showed that FINDY did not inhibit substrate phosphorylation by the WT and Y319E proteins in concentrations of up to 16 μM (Fig. 6g). In contrast, RD0392 inhibited substrate phosphorylation by the WT and Y319E proteins with IC_50_ values of 325 and 813 nM, respectively (Fig. 6g).

### FINDY strengthens interaction between DYRK1A and CDC37

Our recent study indicated that mutations affecting autophosphorylation strengthen the interaction between DYRK1A and the kinase-specific co-chaperone CDC37 (ref. 36). CDC37 assists in the folding of target kinases in cooperation with HSP90 (refs 37, 38). Taipale *et al.* have demonstrated that CDC37/HSP90 is associated with thermodynamically unstable kinases^39^ and that CDC37 fused with luciferase acts as a thermodynamic sensor for kinase structures^40^.

**Figure 7:**
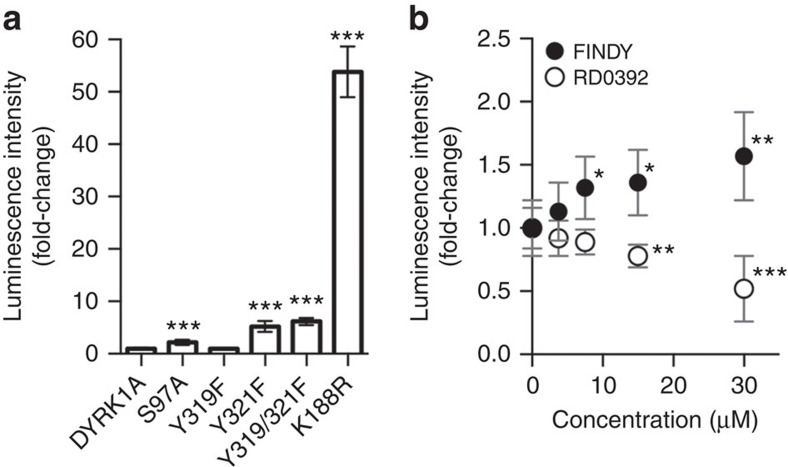
FINDY strengthens the interaction between DYRK1A and the co-chaperone CDC37. (**a**) 293T cells stably expressing CDC37-nanoKAZ were transiently transfected with expression vectors of 3xFLAG-DYRK1A, as were mutants of S97A, Y319F, Y321F, Y319F/Y321F and K188R. Total cell lysates were subjected to the CDC37-nanoKAZ interaction assay (see the Methods for details). Luminescence intensities are shown as fold-changes relative to that of DYRK1A, normalized to the amount of bound protein on a 96-well plate. The graphs represent means±s.d. (*n*=7). Statistical significance was calculated compared with the value of the intact DYRK1A. ***P<0.001. Statistical analysis was performed with the Mann–Whitney *U*-test. (**b**) 293T cells stably expressing CDC37-nanoKAZ were transiently transfected with an expression vector of 3xFLAG-DYRK1A. Twenty-four hours after the transfection, cells were treated with FINDY and RD0392, and incubated for an additional 24 h. Luminescence intensities are shown as fold-changes relative to that at 0 μM, normalized to the amount of 3xFLAG-DYRK1A bound on a 96-well plate. Points on the graph are means±s.d. (*n*=8). Statistical significance was calculated compared with the value at 0 μM. *P<0.05, **P<0.005 and ***P<0.001. Statistical analysis was performed with the Mann–Whitney *U*-test.

To investigate whether the mutation of Ser97 affects the thermodynamic stability of DYRK1A, we examined the interaction between the S97A mutant and CDC37 fused with luciferase nanoKAZ, as described in our recent study^36^, with some modifications (see the Methods for details). The S97A, Y321F, Y319F/Y321F and K188R mutations significantly strengthened the interaction of DYRK1A with CDC37-nanoKAZ (Fig. 7a). The S97A mutant interacted with CDC37-nanoKAZ 2.2-fold more strongly than intact DYRK1A (Fig. 7a). The Y321F, Y319F/Y321F and K188R mutants interacted 5.2-, 6.2- and 54-fold more strongly than intact DYRK1A, respectively (Fig. 7a). Interestingly, the Y321F mutation increased the interaction (Fig. 7a), but did not destabilize the DYRK1A protein in HEK293 cells (Fig. 3a). Thus, the increased interaction with CDC37 is not always correlated with the degradation of DYRK1A mutants. Therefore, another functional role of Ser97 may be involved in the degradation of DYRK1A.

We next investigated whether FINDY decreases the thermodynamic stability of DYRK1A. Treatment with FINDY significantly strengthened CDC37-nanoKAZ interactions in a dose-dependent manner, whereas treatment with RD0392 weakened the interactions (Fig. 7b), suggesting that FINDY induces thermodynamic destabilization of DYRK1A. In addition, we examined the effect of FINDY on the S97A mutant. FINDY also increased the CDC37 interaction with the S97A mutant, but it tended to be less than that with intact DYRK1A (Supplementary Fig. 10). These results suggest that FINDY affected a part of the folding process in a mechanism independent of the Ser97 autophosphorylation.

### Selective inhibition of DYRK1A by FINDY

**Figure 8:**
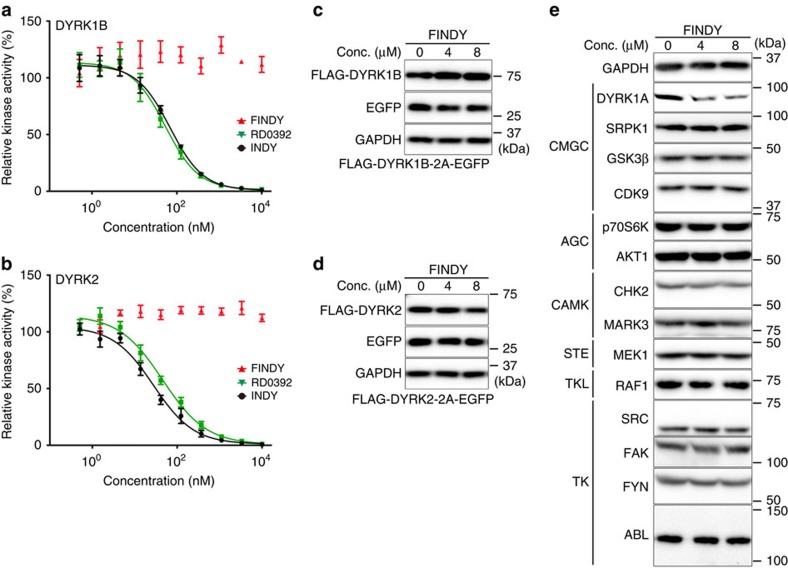
FINDY selectively inhibits DYRK1A. (**a**,**b**) Recombinant DYRK1B (**a**) and DYRK2 (**b**) were incubated with the peptide substrate DYRKtide in the presence of FINDY, RD0392 or INDY. RD0392 and INDY inhibit the kinase activity with IC_50_ values of 53.3 and 69.2 nM, respectively, for DYRK1B, and with IC_50_ values of 45.6 and 27.7 nM, respectively, for DYRK2. The results are presented as means±s.d. (*n*=4). (**c**,**d**) Bicistronic expression of FLAG-DYRK1B (**c**) and FLAG-DYRK2 (**d**) with EGFP in HEK293 cells. FINDY administered at the indicated concentrations (Conc.) did not affect the expression of FLAG-DYRK1B and FLAG-DYRK2. Representative data from the triplicate experiments are shown. (**e**) Intact HEK293 cells were cultured in the presence of FINDY for 3 days. The indicated kinases and GAPDH were detected by western blot analysis using the corresponding antibodies. The categories of the indicated kinases in the kinome are shown. Representative data from the duplicate experiments are shown.

To determine the selectivity of FINDY, potential inhibitory activity was assessed against a panel of 275 kinases using 10 μM of FINDY. Only five other kinases (GSK3β, MARK4, PIM1, PIM3, PLK3) were inhibited by over 75% and none of these showed over 85% inhibition (Supplementary Table 1). In addition, FINDY did not exhibit any inhibitory effect on DYRK1B and DYRK2 in an *in vitro* kinase assay, whereas RD0392 inhibited both kinases in a dose-dependent manner (Fig. 8a,b). DYRK1B, the member of the DYRK family closest to DYRK1A, possesses a conserved serine residue similar to Ser97 of DYRK1A (Supplementary Fig. 11a), the mutation of which also leads to destabilization of DYRK1B (Supplementary Fig. 11b). Therefore, we investigated whether FINDY destabilizes DYRK1B and DYRK2 in cells expressing FLAG-DYRK1B-2A-EGFP and FLAG-DYRK2-2A-EGFP, respectively. Neither the expression of FLAG-DYRK1B nor FLAG-DYRK2 was affected by FINDY (Fig. 8c,d). These results suggest that the transitional intermediate of DYRK1A has a unique structural property distinct from that of DYRK1B and DYRK2. Moreover, FINDY decreased the amount of endogenous DYRK1A in HEK293 cells, whereas no alteration in the endogenous expression of the other 13 kinases was observed (Fig. 8e), indicating that FINDY selectively destabilizes the transitional intermediate of DYRK1A.

**Figure 9:**
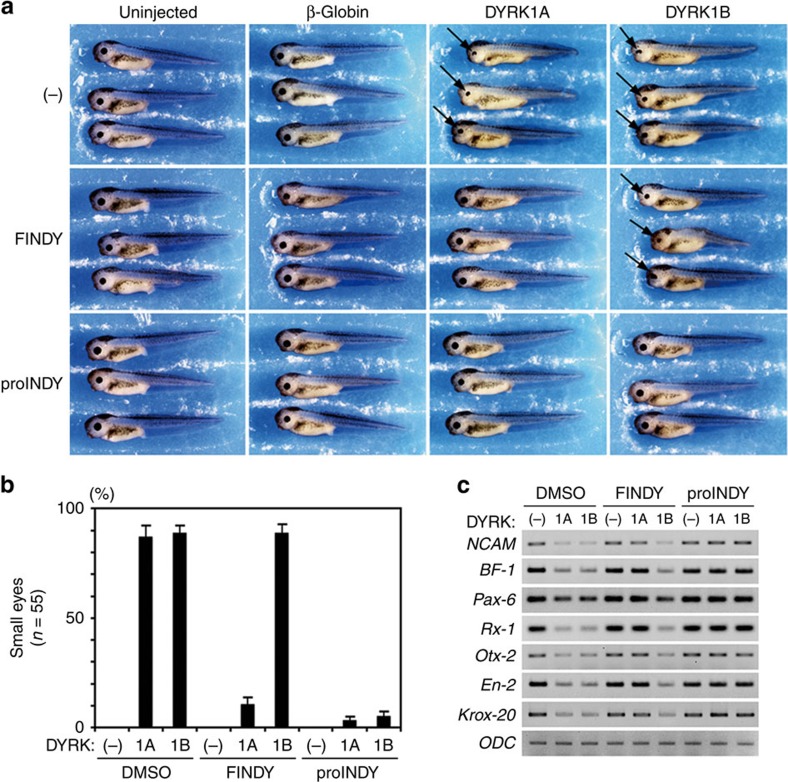
FINDY selectively represses excessive DYRK1A activity in an animal model. (**a**) FINDY rescued the developmental malformation of *Xenopus laevis* embryos as induced by the overexpression of DYRK1A, but did not rescue that induced by DYRK1B. proINDY was effective in both cases. Embryos were treated with the vehicle alone (−), FINDY (2.5 μM) or proINDY (2.5 μM), then two dorsal blastomeres were injected with 750 pg of β*-globin*, *DYRK1A* or *DYRK1B* mRNA at the eight-cell stage. Uninjected embryos were used as controls. Development of the embryos was allowed to proceed to stage 40 in the dark. Representative images are shown. Arrows indicate small eyes. (**b**) Summary of the rescue rates of small eyes as induced by the overexpression of *DYRK1A* or *DYRK1B*. The results are the means±s.e.m. of triplicate experiments (*n*=55). (**c**) RT–PCR analysis of the neural marker genes in the head regions of the embryos at stage 25. Each of the marker genes indicates a specific region: *NCAM* for the pan-neural region, *Brain Factor-1* (*BF-1*) for the forebrain, *Pax-6* and *Rx-1* for the eye, *Otx-2* for the fore-midbrain, *Engrailed-2* (*En-2*) for the mid-hindbrain and *Krox-20* for the hindbrain. *Ornithine decarboxylase* (*ODC*) is an internal control.

We have previously reported that microinjection of *DYRK1A* mRNA into *Xenopus laevis* embryos causes the malformation of neural tissues, and this system has been applied to assess the efficacy of kinase inhibitors *in vivo*^16^. To validate the subtype-selectivity of FINDY in the context of living organisms, we investigated whether FINDY rescues the developmental malformation of *Xenopus* embryos induced by the overexpression of DYRK1A or DYRK1B. *DYRK1A* or *DYRK1B* mRNA was injected into two dorsal blastomeres at the eight-cell stage, resulting in malformation of the eye and the head in stage 40 tadpoles (Fig. 9a). Control β*-globin* mRNA did not cause any morphological alterations compared with uninjected embryos (Fig. 9a). Administration of proINDY, which is an acetylated prodrug with enhanced cell membrane permeability hydrolysed in the cell to form INDY^16^, repressed the malformation induced by both DYRK1A and DYRK1B (Fig. 9a). FINDY rescued the malformation induced by DYRK1A, but not that induced by DYRK1B, demonstrating marked subtype-selectivity (Fig. 9a). Embryos injected with *DYRK1A* mRNA were almost completely rescued with the administration of either FINDY or proINDY (Fig. 9b). Only 10.9% of the tadpoles developed normally with FINDY when injected with *DYRK1B* mRNA, whereas 89.1% were normal in the presence of proINDY (Fig. 9b). In the embryos injected with either *DYRK1A* or *DYRK1B* mRNA, decreased expression of the pan-neural marker *NCAM*, forebrain marker *BF-1*, eye markers *Pax-6* and *Rx-1*, fore-midbrain marker *Otx-2*, mid-hindbrain marker *En-2* and hindbrain marker *Krox-20* were observed (Fig. 9c). FINDY restored the expression of these markers only in DYRK1A-overexpressing tadpoles, whereas proINDY increased the expression of the markers in both DYRK1A- and DYRK1B-overexpressing embryos (Fig. 9c). These results indicate that FINDY rescues the neurological defects induced by DYRK1A in a highly specific manner.

## Discussion

**Figure 10:**
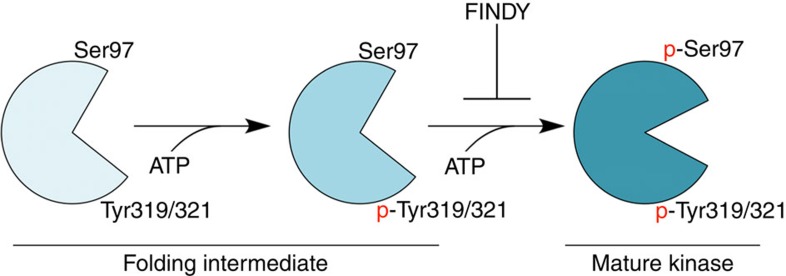
Model of the folding intermediate-selective inhibition by FINDY. In the folding process of DYRK1A, a folding intermediate autophosphorylates Tyr319/321 and subsequently Ser97 in an intramolecular manner, which prevents degradation of DYRK1A. Autophosphorylated DYRK1A takes on a mature conformation. FINDY interferes only with the folding intermediate by preventing the incorporation of ATP, leading to its degradation.

This study provides methods for evaluating chemical compounds that selectively inhibit the transitional intermediates of target kinases. We utilized these methods to identify and evaluate FINDY. Based on the following evidence, we demonstrate that FINDY selectively inhibits a folding intermediate of DYRK1A during its folding process, as illustrated in Fig. 10. First, FINDY suppresses the substrate phosphorylation by DYRK1A only when it is administered before the induced expression (Fig. 1c). Second, neither in the cell-based nor in the *in vitro* assays is FINDY able to inhibit the substrate phosphorylation by the mature DYRK1A (Figs 1c,f, 5e and 6g). Third, FINDY suppressed Ser97 autophosphorylation in cells (Fig. 3d), in the *in vitro* translation (Fig. 5), and in the *in vitro* autophosphorylation assay with the recombinant protein (Fig. 6). The Ser97 autophosphorylation was a one-off intramolecular process that was not catalysed by the mature DYRK1A (Fig. 5f and Supplementary Fig. 7d,e). Furthermore, the CDC37-nanoKAZ interaction assay revealed that FINDY induces the thermodynamic destabilization of DYRK1A (Fig. 7b), suggesting that FINDY affects the folding state of DYRK1A.

Our study demonstrates that FINDY directly suppresses Ser97 autophosphorylation, as discussed below. First, FINDY suppressed Ser97 autophosphorylation in the *in vitro* translation system (Fig. 5), which contained only defined factors, but did not contain mammalian proteins. Second, FINDY suppressed the *in vitro* Ser97 autophosphorylation of the recombinant phosphomimetic mutant (Y319E) of DYRK1A, whereas tyrosine autophosphorylation was not suppressed by FINDY (Fig. 6 and Supplementary Fig. 5). These results are consistent with the results from the cellular assays (Fig. 3d). Several lines of evidence demonstrated that the mature DYRK1A was not able to catalyse Ser97 autophosphorylation (Figs 5f and 6a,b and Supplementary Fig. 7d,e). Thus, the kinase form that catalyses Ser97 autophosphorylation is not the mature form. Therefore, the recombinant Y319E probably reflects an aspect of the folding intermediate. Third, FINDY blocked the incorporation of biotin-ATP into DYRK1A (Fig. 4), indicating two possibilities. One is that FINDY occupies the ATP-binding pocket. The other is that FINDY affects the biotin-ATP incorporation by binding to an allosteric site, which may affect the ATP binding to the pocket. FINDY possesses the same polar groups of RD0392 that targets the ATP-binding pocket of DYRK1A (Supplementary Figs 2 and 3). Thus, the former scenario is most likely. The selectivity of FINDY for the Ser97 and tyrosine autophosphorylation suggests that the transitional state targeted by FINDY is distinct to that of tyrosine autophosphorylation. Further structural studies are necessary to conclude this discussion.

The phosphomimetics experiment suggested that the phosphorylated tyrosine residue in the activation loop is required for the subsequent Ser97 autophosphorylation (Fig. 6). Although intramolecular tyrosine autophosphorylation in the activation loop has been suggested to be a one-off inceptive event to achieve full activity during the co-translational folding process of *Drosophila* DYRKs^17^,^19^,^41^, it is controversial whether the autophosphorylation of these tyrosine residues is essential for the catalytic activity of mammalian DYRK1A^21^,^33^. The double mutation of Y321F/Y319F in the activation loop destabilized FLAG-DYRK1A, whereas a single mutation gave a much smaller effect (Fig. 3a). Our study indicates that the autophosphorylation of at least one of these tyrosine residues is required for Ser97 autophosphorylation, suggesting that Ser97 autophosphorylation acts as a quality control mechanism. It remains elusive how Ser97 autophosphorylation regulates the stability of DYRK1A and why Ser97 autophosphorylation is a one-off process. In comparison to the *in vitro* translation (Fig. 5a), we did not observe the faster migrating band corresponding to the Ser97 non-phosphorylated DYRK1A in the cultured mammalian cells, which may be subjected to degradation during the folding process. The actual amount of the Ser97 autophosphorylation during the folding process in mammalian cells also remains unclear.

Although RD0392 inhibited the Ser97 autophosphorylation in the *in vitro* translation experiment (Fig. 5c,d) and in the autophosphorylation assay using the recombinant DYRK1A protein (Fig. 6e,f), RD0392 did not decrease the FLAG-DYRK1A protein in the cultured cells (Fig. 2d). This discrepancy is possibly due to low cell membrane permeability of RD0392. FINDY has a hydrophobic trimethylsilyl group that may serve to enhance the cell membrane permeability compared with that of RD0392. RD0392 inhibited the Tyr autophosphorylation of the recombinant DYRK1A protein (Fig. 6e,f). In contrast, RD0392 did not inhibit the Tyr autophosphorylation in the *in vitro* translation (Supplementary Fig. 8c). This indicates the possibility that RD0392 is capable of inhibiting post-translational Tyr autophosphorylation, but co-translational Tyr autophosphorylation is resistant to RD0392.

An interesting point in the biotin-ATP experiment is that INDY and RD0392 failed to inhibit biotinylation, whereas FINDY, on the other hand, did not (Fig. 4b). The most likely explanation is that FINDY has a slower rate of dissociation (*K*_off_) from the intermediate of DYRK1A than the *K*_off_s that INDY and RD0392 have to the mature form. This slow *K*_off_ for FINDY may be due to thermodynamic destabilization by FINDY, which is distinct from the effects of INDY and RD0392. FINDY uniquely strengthened the interaction between DYRK1A and CDC37-nanoKAZ (Fig. 7b). In contrast, RD0392 and INDY weakened the interaction (Fig. 7b)^36^, which is in good agreement with previous reports that canonical kinase inhibitors weaken CDC37 interactions^39^,^40^,^42^. A previous study has demonstrated that CDC37 binding competes with ATP for the pocket of its client kinase^42^, suggesting that the enhanced binding of CDC37 plays a role in the suppression of the biotinylation. Furthermore, the thermodynamic destabilization caused by FINDY may also cause hydrophobic aggregation of the DYRK1A protein, which masks the pocket and prevents the incorporation of ATP. Thus, we consider that thermodynamic destabilization by FINDY may result in strong competition with the biotin-ATP.

The SPHINKS assay consists of cells expressing DYRK1A and its substrate fused with a DD tag and the detection of TAU phosphorylation. This assay was developed to evaluate the effect of chemical compounds on the folding process of DYRK1A; however, it is not suitable for high-throughput screening of large-scale chemical libraries. Thus, the SPHINKS assay should be utilized as a second or third screening assay for a small number of chemical compounds that were roughly selected from the library by a robust high-throughput assay. The *in vitro* translation and autophosphorylation assays would be better methods for developing high-throughput assays. Some small molecules may show apparent selectivity in the SPHINKS assay because of slower actions, such as low solubility, pro-drug activation or slow uptake into cells. Thus, the properties of small molecules should be carefully examined.

This study demonstrates that targeting transitional intermediates is a promising strategy for the development of novel kinase inhibitors. Intramolecular autophosphorylation in the activation loop has been demonstrated not only in DYRK1A, but also in other kinases, such as DYRK2 (ref. 19), GSK3β (ref. 18), ERKs^43^ and the Parkinson's disease-associated kinase LRRK2 (refs 44, 45). In addition, autophosphorylation outside the activation loop has been reported in C-RAF^46^ and protein kinase A (PKA)^47^. We believe that intermediate-selective inhibitors for these kinases can be identified. The close structural similarity between the intermediate-selective inhibitors and the canonical inhibitors raises the possibility that canonical ATP-competitive kinase inhibitors can be converted to intermediate-selective inhibitors with only a slight modification of the common chemical scaffold (Supplementary Figs 12 and 13). Numerous compounds targeting various kinases have been rejected in *in silico* or *in vitro* drug screening, but some could potentially inhibit a transitional intermediate of the target kinase. The methods developed in this study can be utilized to reassess these compounds and provide opportunities to rediscover selective kinase inhibitors.

The dynamic state of the transitional intermediate may provide conformational diversity for the inhibitor-binding pocket. It is possible to purify the metastable intermediate of the DYRK1A protein produced in the presence of FINDY, as we have preliminarily tried in Figs 4 and 5. Further studies using biophysical techniques are necessary in order to analyse the intermediate state targeted by FINDY. Resolving the intermediate states of kinases would expand the chemical options available for the design of selective inhibitors.

## Methods

### Materials

Small molecules were prepared using standard synthetic procedures, as described in Supplementary Methods. INDY and proINDY were prepared as described previously^16^. These compounds were dissolved in dimethylsulphoxide (DMSO) at 10 mM as a stock solution. Calyculin A (Cell Signaling Technology), okadaic acid (Tocris Bioscience) and cantharidic acid (Abcam) were purchased. The polyclonal antibody against p-Ser97 was produced by immunizing rabbits with a synthetic phospho-peptide, C-PEG-PLRKLS*VDLIK, which was coupled to KLH via the free cysteine, with S* representing phosphoserine. The specific antibody was purified by phospho-peptide-conjugating resin followed by a subtraction step on a column containing non-phospho-peptide. The polyclonal antibody against p-Ser97 (1/2,000) and antibodies listed below were diluted with Can Gel Signal Immunoreaction Enhancer Solution (TOYOBO) in western blot analysis. Mouse monoclonal anti-DYKDDDDK (FLAG) tag (1/1,000, clone 1E6, 018-22381/Lot: CTR5949) and anti-GFP (1/1,000, mFX75, 012-22541/Lot: STK2113) antibodies were purchased from Wako Pure Chemical Industries, mouse monoclonal anti-HA (1/1,000, clone HA124, 06340-54/Lot: M2E8170) antibody from Nacalai Tesque, mouse monoclonal anti-GAPDH (1/10,000, clone 6C5, AM4300/Lot: 1311029) from Thermo Fisher Scientific, mouse monoclonal anti-TAU (1/3,000, clone TAU-5, 577801/Lot: D00100518) from Calbiochem (Merck), rabbit polyclonal antibody against pT212 of TAU (1/3,000, 44740G/Lot: 1392715A) from Life Technologies, mouse monoclonal anti-phosphotyrosine (1/3,000, 4G10 Platinum, 05-1050/Lot: 2567063) from Millipore, mouse monoclonal anti-GST-tag (1/3,000, clone GT5, M209-3/Lot: 001) from MBL, mouse monoclonal anti-SRPK1 (1/3,000, clone 12/SRPK1, 611072) from BD Transduction Laboratories (BD Biosciences), mouse monoclonal anti-CDK9 (1/3,000, clone D-7, sc-13130/Lot: c0613) from Santa Cruz Biotechnology and mouse monoclonal anti-neuronal class III β-Tubulin (1/3,000, clone TUJ1, MRB-435P/Lot: E10045BF) from Covance. Rabbit polyclonal anti-DYRK1A (1/3,000, #2771S/Lot: 1), p70S6K (1/3,000, #9202S/Lot: 8), CHK2 (1/3,000, #2662S/Lot: 5), MARK3 (1/3,000, #9311BC/Lot: 1), RAF1 (1/3,000, #9422BC/Lot: 5), FAK (1/3,000, #3285BC/Lot: 6), FYN (1/3,000, #4023BC/Lot: 2) and ABL (1/3,000, #2862BC/Lot: 11) antibodies were obtained from Cell Signaling Technology. Rabbit monoclonal anti-GSK3β (1/3,000, clone 27C10, #9315S/Lot: 4), AKT1 (1/3,000, clone C73H10, #2938BC/Lot: 2), MEK1 (1/3,000, clone 30C8, #9146BC/Lot: 1) and SRC (1/3,000, clone 32G6. #2123BC/Lot: 3) antibodies were purchased from Cell Signaling Technology. HRP-linked anti-rabbit (1/4,000, NA9340V/Lot: 9611780) and anti-mouse (1/4,000, ab5887/Lot: GR16899-9) IgG were purchased from GE Healthcare and Abcam, respectively. HRP-labelled streptavidin (1/2,000, 474-3000/Lot: 060065) was obtained from Kirkegaard & Perry Laboratories.

### Vector construction

In brief, the bicistronic expression vectors for the DYRKs were constructed with pcDNA5/FRT/TO (Life Technologies). DD-TAU and DD-FLAG-DYRK1A were inserted into the pCAGIPuro vector. To prepare *in vitro* transcribed mRNAs for injection, DYRKs were inserted into the pCS2+ vector. pCold (Takara Bio) and pET28a(+) vectors were used for the preparation of recombinant protein in *E. coli* cells. The pET28a(+) vector was also used for the *in vitro* translation experiments. PCR-amplified fragments of the human microtubule-associated protein TAU (0N4R) and FLAG-tagged DYRK1A were fused in-frame to the carboxyl-terminus of DD tag by the overlap-extension PCR method to generate DD-TAU and DD-FLAG-DYRK1A, respectively, as described previously^48^, with some modifications. The combined fragments were inserted into the pCAGIPuro vector, an IRES-based bicistronic expression vector where the gene of interest and a puromycin-resistant gene are expressed from a single mRNA, which enables almost all of the cells selected with puromycin to express the gene product. A PCR-amplified fragment of FLAG-tagged DYRK1A was subcloned into pcDNA5/FRT/TO. For the bicistronic cassette using a 2A peptide, PCR-amplified fragments of FLAG-tagged DYRKs were fused in-frame to the amino-terminus of EGFP via the F2A peptide sequence by the overlap-extension PCR method. The combined fragments were inserted into pcDNA5/FRT/TO (Life Technologies). The site-directed mutagenesis of DYRK1A and DYRK1B was performed as described previously^49^, with some modifications. The reconstituted vector sequences are available upon request.

### Cell culture and transfection

Flp-In/T-REx HEK293 (Life Technologies) and intact HEK293 cells were maintained in low-glucose DMEM (Nacalai Tesque) supplemented with 10% fetal bovine serum (FBS; Nichirei Biosciences), 100 U ml^−1^ of penicillin and 100 μg ml^−1^ of streptomycin (Nacalai Tesque). Cells were transfected with plasmid DNAs using polyethylenimine MAX (Polysciences) as described previously^50^, then selected with hygromycin B (Life Technologies) for the pcDNA5/FRT/TO vectors and/or with puromycin (Nacalai Tesque) for the pCAGIPuro vectors to establish stable cell lines.

### SPHINKS assay

Our original synthetic chemical library used in this study consisted of newly synthesized structural derivatives of ATP-competitive inhibitors of DYRK1A, which had been identified by an *in vitro* kinase assay in our laboratory. Flp-In/T-REx HEK293 cells transfected with pcDNA5/FRT/TO-FLAG-DYRK1A and pCAGIPuro-DD-TAU were seeded on polyethylenimine-coated six-well plates with 2 ml of DMEM and 1% FBS. Cells were then cultured 24 h later in the presence of 1 μg ml^−1^ of doxycycline (Nacalai Tesque) for 16 h, then treated with 1 μM of Shield-1 (Clontech, Takara Bio) for 2 h. Small molecules from our chemical library were diluted in 1 ml of DMEM and 1% FBS, then administered into the cells at a final concentration of 10 μM (0.1% DMSO), as illustrated in Fig. 1a. Total cell lysates were prepared with SDS-urea buffer (50 mM Tris-HCl, pH 7.0, 1% SDS, 4 M urea, 10% glycerol, 20 mM dithiothreitol, 0.5 mg ml^−1^ BSA), then analysed with SDS–polyacrylamide gel electrophoresis (SDS–PAGE) followed by western blot analysis using antibodies against phospho-TAU (pT212) (1/3,000) and total TAU (1/3,000) proteins (Supplementary Fig. 14). Details are summarized in Supplementary Table 2.

### Immunoprecipitation of FLAG-DYRK1A

HEK293 cells were cultured in the presence of 1 μg ml^−1^ of doxycycline, 1 μM of Shield-1, 1 μM of epoxomicin and a proteasome inhibitor (Peptide Institute), with or without 10 μM of small molecules for 5 h. The cells were lysed in 50 mM Tris (pH 7.4), 150 mM NaCl, 0.5 mM EDTA, 1% Empigen BB and protease/phosphatase inhibitor cocktails (Nacalai Tesque). The clarified cell lysates were processed with anti-FLAG (clone M2) beads (F2426, Sigma-Aldrich) and the bound proteins were then eluted with SDS-urea buffer, then analysed with SDS–PAGE followed by western blot analysis (Supplementary Fig. 14).

### Biotin transfer experiment

Biotinylation of the lysine in the ATP-binding pocket was performed using the Pierce Kinase Enrichment Kit with an ATP Probe (Thermo Fisher Scientific), according to the manufacturer's instructions with some modifications. HEK293 cells were cultured with 1 μg ml^−1^ of doxycycline and 1 μM of epoxomicin, with or without 10 μM of small molecules for 5 h, then harvested and lysed in Pierce IP Lysis Buffer containing 0.5% Empigen BB. The clarified cell lysates were processed with anti-FLAG (M2) beads, then equilibrated with the kit's reaction buffer. The beads were subsequently reacted with 20 μM of the kit's ActivX Desthiobiotin-ATP probe for 30 min at room temperature. The bound proteins were eluted with SDS-urea buffer, then analysed with SDS–PAGE followed by western blot analysis (Supplementary Fig. 14).

### *E. coli*-based *in vitro* translation assay

The PURExpress kit (New England Biolabs) was employed. DYRK1A was expressed at 37 °C for 40 min in a 12.5-μl reaction mixture containing 30 ng of pET28a(+)-FLAG-DYRK1A. For inhibitor experiments, FINDY or RD0392 was added prior to the initiation of transcription. For immunoprecipitation, 100 μl of PBS containing 0.5% Empigen BB, 0.5 mg ml^−1^ BSA and 5 mM EDTA was added into the reaction mixture, then incubated with anti-FLAG (M2) beads at 4 °C for 6 h. The samples were prepared with an SDS-urea buffer, then analysed with SDS–PAGE followed by western blot (Supplementary Fig. 14). The band intensities were measured using the LAS-3000 (Fujifilm) and Multi Gauge software (Fujifilm).

For the phosphatase experiment, immunoprecipitated FLAG-DYRK1A bound on the beads was incubated in a total volume of 100 μl containing 1,600 U of lambda protein phosphatase for 2 h at 30 °C. After washing the beads with autophosphorylation reaction (AR) buffer (see below) containing 0.1 mg ml^−1^ of BSA (Sigma-Aldrich), the beads were incubated with the indicated concentrations of ATP and sodium orthovanadate (1 mM) for 2 h at 37 °C and prepared with the SDS-urea buffer. To check the residual phosphatase activity, non-treated immunoprecipitated FLAG-DYRK1A bound on the beads was added into the reaction.

For the *in vitro* kinase assay, 0.5 μl of the reaction mixture was incubated in a total volume of 25 μl of AR buffer containing 0.1 mg ml^−1^ of BSA, 100 μM of ATP, 50 μM of DYRKtide peptide (Anaspec) and the indicated concentrations of the small molecules for 2 h at 37 °C. The consumption of ATP was measured with ADP-Glo Kinase Assay kit (Promega Corporation).

### *In vitro* autophosphorylation assays

*E. coli* strain Rosetta(DE3)pLysS was transformed with the pCold-I-GST-DYRK1A-TS vector. The cells were cultured with IPTG (final 1 mM) for 24 h at 6 °C, then lysed with GET buffer (20 mM HEPES-KOH, pH 8.0, 150 mM NaCl, 1 mM EDTA, 2 mM dithiothreitol, 10% glycerol, 0.5% Empigen BB, 0.5% Triton X-100). Clarified cell lysates were loaded onto a *Strep*-Tactin Sepharose column (IBA) and the bound proteins were then eluted with GET buffer containing 2.5 mM desthiobiotin (IBA) and 1 mg ml^−1^ BSA. The eluates were subsequently applied to GST-Accept resin (Nacalai Tesque). The resin was washed extensively, first with GET buffer, then with AR buffer (5 mM MOPS, pH7.2, 2.5 mM β-glycerol-phosphate, 5 mM MgCl_2_, 1 mM EGTA, 0.4 mM EDTA, 2 mM tris(2-carboxyethyl)phosphine). The GST-DYRK1A-TS (0.5 pmol) bound on the resin (bed vol. 2 μl) was suspended in 100 μl of AR buffer, mixed with 1 μl of a stock DMSO solution of FINDY or RD0392, then incubated at 37 °C for 30 min. We then added 5 μl of 2 mg ml^−1^ BSA to the suspension (final concentration of 0.1 mg ml^−1^), followed by incubation at 37 °C for 30 min. We then added 5 μl of ATP solution to the suspension (final concentrations of 100 μM and 1 mM), which was subsequently incubated at 37 °C for 2 h. The bound proteins were eluted with SDS-urea buffer, then analysed with SDS–PAGE followed by western blot (Supplementary Fig. 14).

The band intensities were measured using the ChemiDoc MP Imaging System (Bio-Rad). In Fig. 6d,e, the relative activity of the phosphorylation was calculated from the ATP-dependent increase in the intensity of the p-Ser97 and p-Tyr signals by subtracting the band intensity in the absence of ATP from that in the presence of ATP.

For the *in vitro* kinase assay, 2 μl (bed vol.) of the resin was incubated in a total volume of 25 μl of AR buffer containing 0.1 mg ml^−1^ of BSA, 100 μM of ATP, 50 μM of DYRKtide peptide and the indicated concentrations of the small molecules, for 2 h at 37 °C. The consumption of ATP was measured with ADP-Glo Kinase Assay kit.

### CDC37-nanoKAZ interaction assay

The CDC37-nanoKAZ interaction assay was performed as described previously^36^, with some modifications. 293T cells stably expressing CDC37-nanoKAZ were transiently transfected with the expression vector 3xFLAG-DYRK1A, then cultured in a 96-well plate. Twenty-four hours after the transfection, FINDY and RD0392 were added to the wells at the indicated concentrations and the cells were cultured for an additional 24 h. The cells were lysed in ice-cold HENG buffer (50 mM HEPES-KOH, pH 7.9, 150 mM NaCl, 20 mM Na_2_MoO_4_, 2 mM EDTA, 5% glycerol, 0.5% Triton X-100) containing protease inhibitor cocktail (Nacalai Tesque) on ice. The cleared lysates were added to 96-well plates (OptiPlate-96 HB; Perkin Elmer) coated with antibody against FLAG peptide (clone M2, F3165/Lot: SLBH1191V) diluted in sodium bicarbonate buffer (pH 9.6), and incubated at 4 °C for 3 h. The wells were wash three times with ice-cold HENG buffer and then a luminescence assay was performed. The luminescence of the captured CDC37-nanoKAZ was measured using the Centro LB 960 Microplate Luminometer (Berthold Technologies). A reaction mixture containing *bis*-coelenterazine (final concentration of 0.1–1 μg in 100 μl) in PBS containing 0.02% Tween-20 and 20 mM EDTA was injected. The luminescence intensity was recorded at 0.1 s intervals for 10 s or 10 min. The maximum luminescence intensity (*I*_max_), represented in relative luminescence units, was used in the study. The captured 3xFLAG-DYRK1A was also measured using HRP-conjugated anti-FLAG antibody (clone M2, A8592/Lot: SLBH1183V, Sigma-Aldrich) and TMD Super Sensitive One Component HRP Microwell Substrate (SurModics, Eden Prairie). The relative amount of protein bound on the well was estimated from the absorbance. The relative luminescence intensities were normalized to the amount of captured 3xFLAG-DYRK1A, and calculated as the fold-change relative to the value at 0 μM of the compounds.

### Handling of *Xenopus laevis* embryos and reverse transcription (RT)–PCR analysis

*DYRK1A* and *DYRK1B* mRNA, which were synthesized from linearized vectors with the mMessage mMachine Kit (Life Technologies), were injected into two dorsal blastomeres at the eight-cell-stage for RT–PCR analysis and observation of the embryo phenotypes. Solutions containing proINDY or FINDY (2.5 μM each) were used to treat the embryos just after fertilization. The head regions of the injected embryos were dissected at the neural stage (stage 25) and the total RNA was isolated using TRIzol (Life Technologies). cDNA synthesis was carried out using Moloney murine leukaemia virus reverse transcriptase (Life Technologies). The sequences of the primer pairs were as follows^51^,^52^: NCAM, 5′-GCGGGTACCTTCTAATAGTCAC-3′ and 5′-GGCTTGGCTGTGGTTCTGAAGG-3′; Brain factor 1 (BF-1), 5′-TCAACAGCCTAATGCCTGAAGC-3′ and 5′-GCCGTCCACTTTCTTATCGTCG-3′; Pax-6, 5′-CAGAACATCTTTTACCCAGGA-3′ and 5′-ACTACTGCTAATGGGAATGTG-3′; Rx1, 5′-CCCCAACAGGAGCATTTAGAAGAC-3′ and 5′-AGGGCACTCATGGCAGAAGGTT-3′; Otx-2, 5′-GGATGGATTTGTTGCACCAGTC-3′ and 5′-CACTCTCCGAGCTCACTTCTC-3′; En-2, 5′-CGGAATTCATCAGGTCCGAGATC-3′ and 5′-GCGGATCCTTTGAAGTGGTCGCG-3′′; Krox-20, 5′-AACCGCCCCAGTAAGACC-3′ and 5′-GTGTCAGCCTGTCCTGTTAG-3′; ornithine decarboxylase (ODC), 5′-GTCAATGATGGAGTGTATGGATC-3′ and 5′-TCCATTCCGCTCTCCTGAGCAC-3′. No randomization was used, and no blinding was done.

### Mass spectrometry

HEK293 cells were cultured in the presence of 1 μg ml^−1^ doxycycline and 1 μM epoxomicin, with or without 10 μM FINDY for 8 h. The cells were then harvested and lysed in 50 mM Tris (pH 7.4), 150 mM NaCl, 0.5 mM EDTA, 1% Empigen BB and protease/phosphatase inhibitor cocktails (Nacalai Tesque). The clarified cell lysates were processed with anti-FLAG (M2) beads (Sigma-Aldrich) and the bound proteins were then eluted with glycine buffer (pH 3.0), which was immediately neutralized with 1 M Tris (pH 8.0). After reductive alkylation, the samples were trypsinized, then processed using ZipTip pipette tips containing C18 (Millipore). The digested and purified peptides were analysed by LC-MS/MS on TripleTOF 5600 (AB SCIEX). Database searches were performed using ProteinPilot Software (AB SCIEX).

### RI-*In vitro* kinase assay

The RI-*in vitro* kinase assay was performed as described previously^16^,^53^, with some modifications. His-tagged human DYRK1A kinase domain (residues 127–485) was produced in *E. coli* then purified with Ni-NTA. GST-tagged full-length human DYRK1B recombinant proteins and His-tagged full-length human DYRK2 recombinant proteins were obtained from Life Technologies and Millipore, respectively. The kinase reaction was performed in a reaction mixture containing serially diluted inhibitors, 10 mM MOPS-KOH (pH 7.0), 10 mM magnesium acetate, 200 μM EDTA, 1 μM ATP, 0.1–0.4 μCi [γ-^32^P]ATP, 5 μM DYRKtide and 0.1–1 μg recombinant kinase, for a final volume of 25 μl. The final concentration of DMSO was adjusted to 1%, regardless of the inhibitor concentration. The reaction mixture was incubated at 30 °C for 10 min and phosphoric acid (final 5%) was then added to stop the reaction. We then dispensed 25 μl of the reaction mixture onto P81, a phosphocellulose membrane (Whatman, GE Healthcare), and washed four times in 5% phosphoric acid. Cherenkov light from the incorporated ^32^P was measured using a liquid scintillation counter. The kinase assay conditions, including the incubation period and the concentration of the kinases and substrates, were optimized to maintain linearity during the incubation. The net radioactivity was determined by subtracting the background count from the reaction mixture without kinase, and the data were expressed as a percentage of the control sample containing the solvent.

For the ATP kinetics, the same methodology was applied as above, except that the ATP concentration ranged from 1.25 to 40 μM. The amount of incorporated ^32^P was calculated from the standard line. The *K*_m_, *K*_i_ and *V*_max_ values were calculated with Prism5 software (GraphPad Software) using the competitive inhibition model.

### Inhibitory profiling against 275 recombinant kinases

The effects of FINDY against 275 kinases, listed in Supplementary Table 1, at an inhibitor concentration of 10 μM, were tested using the QuickScout Screening Assist Mobility Shift Assay or an immobilized metal ion affinity-based fluorescence polarization screening express kit with ATP concentration at *K*_m_ or 1 mM (Carna Biosciences). Detailed information on the assay conditions is available on the website of Carna Biosciences (http://www.carnabio.com).

### Docking simulation

We selected the crystal structure of the human DYRK1A/INDY complex (RCSB Protein Data Bank accession code 3ANQ)^16^ as the starting model for the simulation. Docking simulations of RD0392 to DYRK1A were performed with CDOCKER and related modules in Discovery Studio 3.0 (Accelrys). As the same procedure failed to produce any reasonable binding model for FINDY, its complex with DYRK1A was built by manually modifying that for DYRK1A/RD0392 obtained above, maintaining the common moiety of the two compounds. The energy of the hypothetical DYRK1A/FINDY complex was then minimized with the same program suite.

### Statistical analysis

Statistical analysis of the experimental data (Figs 6b and 7 and Supplementary Figs 7e, 9e and 10) was performed with the Mann–Whitney *U*-test. The data in Fig. 6d were analysed with the paired *t*-test. The results are shown as means±s.d. with *P* values (*P<0.05, **P<0.01, ***P<0.001). The data were fitted to a four-parameter logistic curve (variable slope) for Hill slope determination, from which the IC_50_ and EC_50_ values were calculated using Prism 6.0 (GraphPad Software).

## Additional information

**How to cite this article:** Kii, I. *et al.* Selective inhibition of the kinase DYRK1A by targeting its folding process. *Nat. Commun.* 7:11391 doi: 10.1038/ncomms11391 (2016).

## Supplementary Material

Supplementary Information Supplementary Figures 1-14, Supplementary Tables 1-2, Supplementary Methods and Supplementary Reference.
