# Supplementary material for: Selective inhibition of the kinase DYRK1A by targeting its folding process
Source: Nat Commun. 2016 Apr 22;7:11391. doi: 10.1038/ncomms11391 (PMC4844702; doi:10.1038/ncomms11391)
Supplement: Supplementary Information — Supplementary Figures 1-14, Supplementary Tables 1-2, Supplementary Methods and Supplementary Reference. [file ncomms11391-s1.pdf]

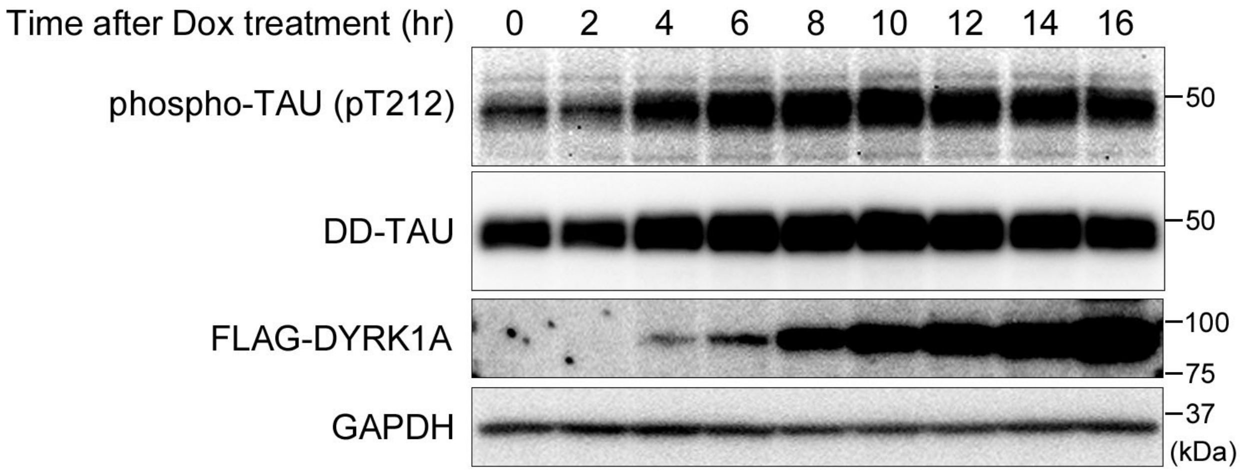

Supplementary Figure 1: Time course of TAU phosphorylation by mature DYRK1A after doxycycline treatment

HEK293 cells were treated with doxycycline for the indicated time and DD-TAU was then stabilized by Shield-1 treatment for an additional 2 h. Total cell lysates were subjected to SDS-PAGE, followed by Western blot analysis with their corresponding antibodies. Representative data from the triplicate experiments is shown.

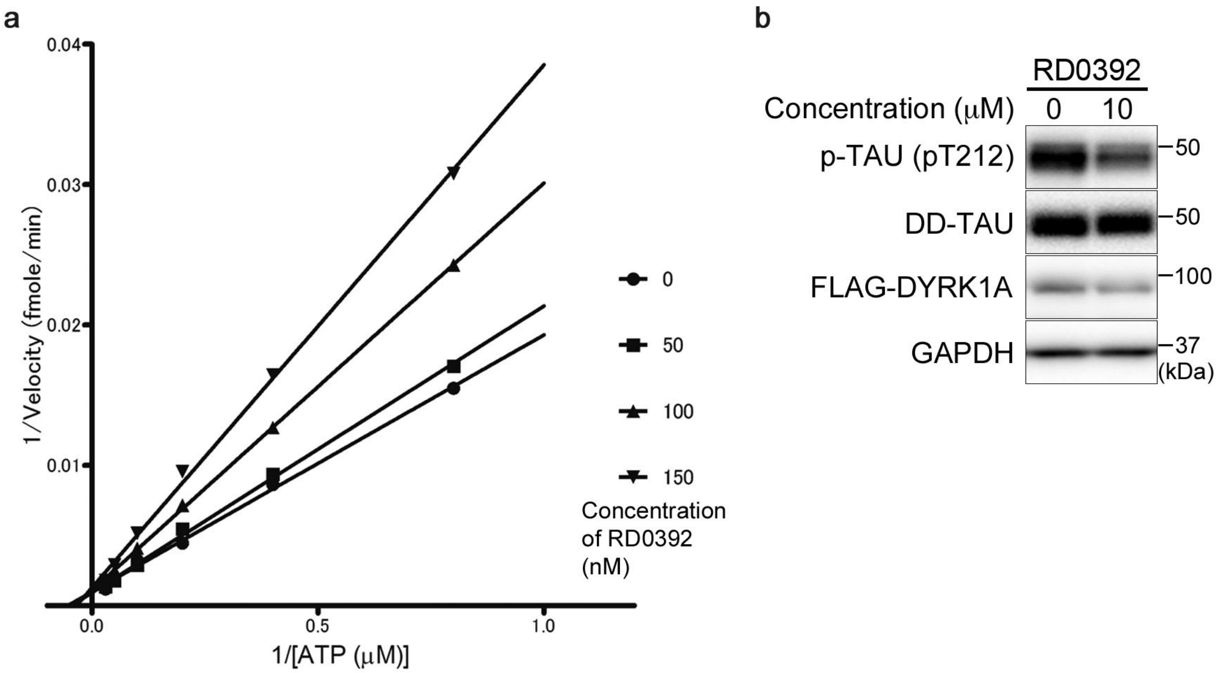

Supplementary Figure 2: RD0392 is a potent ATP-competitive inhibitor of DYRK1A

(**a**) Double-reciprocal plots showing the competitive inhibition of ATP by RD0392. DYRK1A kinase activity was measured at the indicated concentrations of RD0392 and ATP. Reciprocal velocity was plotted versus 1/[ATP]. K_m = 25.2 μM, $V_{\max}$ = 1328 fmol/min/μg, and K_i = 132.2 nM. The double-reciprocal plots show first-order inhibitory kinetics, demonstrating that RD0392 competes with ATP for a single site on DYRK1A, the ATP-binding pocket.

(**b**) RD0392 suppresses TAU phosphorylation in HEK293 cells. Representative data from the duplicate experiments is shown.

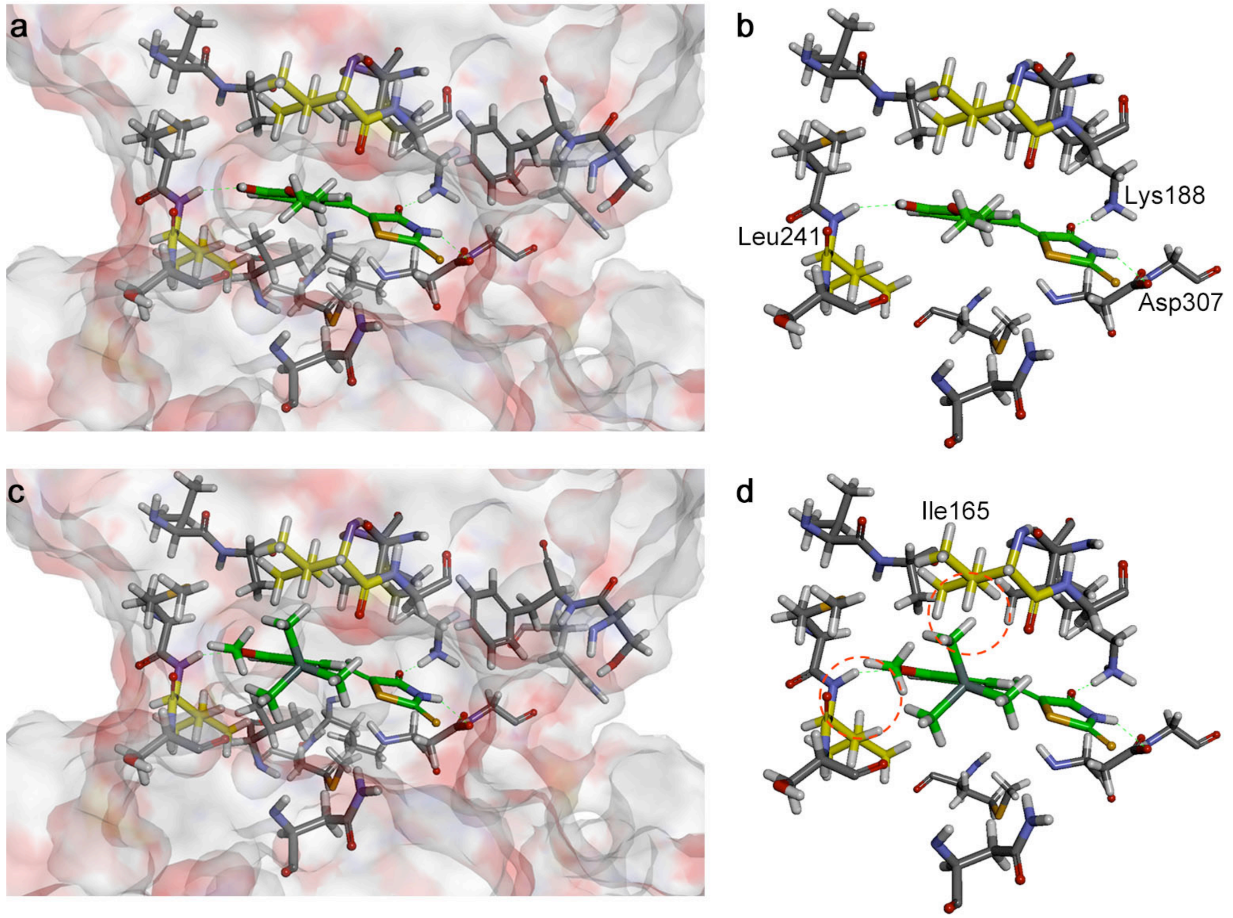

Supplementary Figure 3: FINDY does not fit into the ATP-binding pocket of mature DYRK1A

A docking simulation of RD0392 to the kinase domain of DYRK1A was performed. This model was built based on the coordinates of the co-crystal structure of DYRK1A and INDY, which we have disclosed in a previous study [1]. The docking simulation predicted that RD0392 fits into the ATP-binding pocket (**a**). The three polar groups of RD0392 maintain the spatial configuration of the ligand via three hydrogen bonds: the phenolic hydroxyl group to the main chain nitrogen of Leu241, and the oxygen and nitrogen of the rhodanine moiety to the side-chain amino group of Lys188 and the side-chain oxygen group of Asp307, respectively (**b**). The predicted results may correspond to the fact that RD0392 is a more potent ATP-competitive inhibitor than INDY (shown in **Fig. 1f**), which only formed two polar interactions, with Leu241 and Lys188, in the ATP-binding pocket [1].

FINDY was superimposed onto the predicted model of RD0392 and the binding energy was re-calculated. The resulting docking pose with the most favourable binding energy is shown in (**c**). The three polar interactions were conserved in FINDY; however, the bulky hydrophobic trimethylsilyl moiety and the methoxy moiety of FINDY conflicted with the

side-chain of Ile165 and the main-chain of Leu241, respectively, and thus it did not fit well (**d**). This model agrees with the observation that FINDY does not inhibit mature DYRK1A.

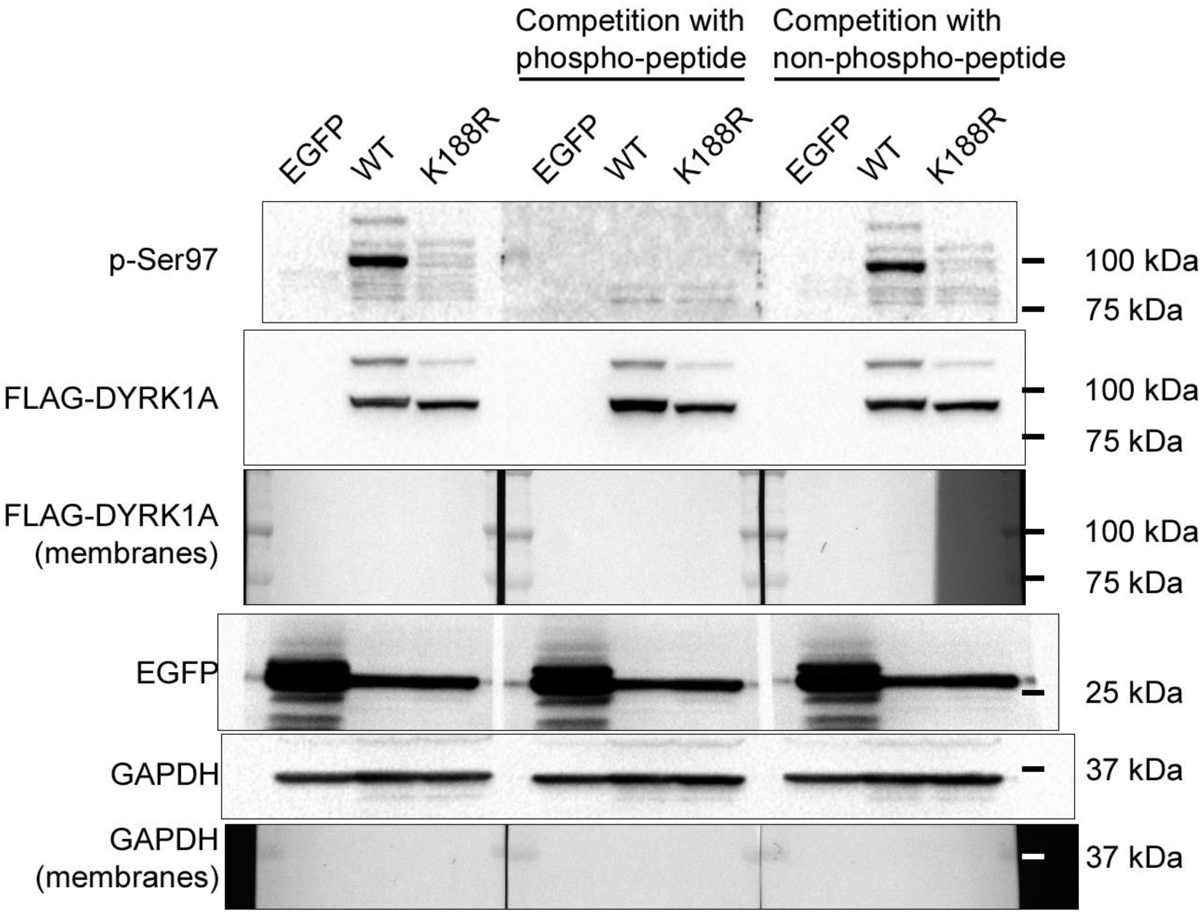

Supplementary Figure 4: Peptide competition experiment of the antibody generated against p-Ser97

293T cells were transiently transfected with the expression vectors of EGFP, DYRK1A (WT), and the kinase-dead mutant (K188R), and the proteins were overexpressed. Twenty-four hours after the transfection, total cell lysates were subjected to SDS-PAGE, followed by Western blot analysis. The transferred membranes were incubated with the rabbit polyclonal p-Ser97 antibody and phosphorylated or non-phosphorylated Ser97 peptides, which were used for immunization and purification. The membranes were aligned side by side in the same tray containing the HRP substrate and luminescence images were captured. The membranes also reacted with antibodies against FLAG, EGFP, and GAPDH. Representative data from the duplicate experiments is shown.

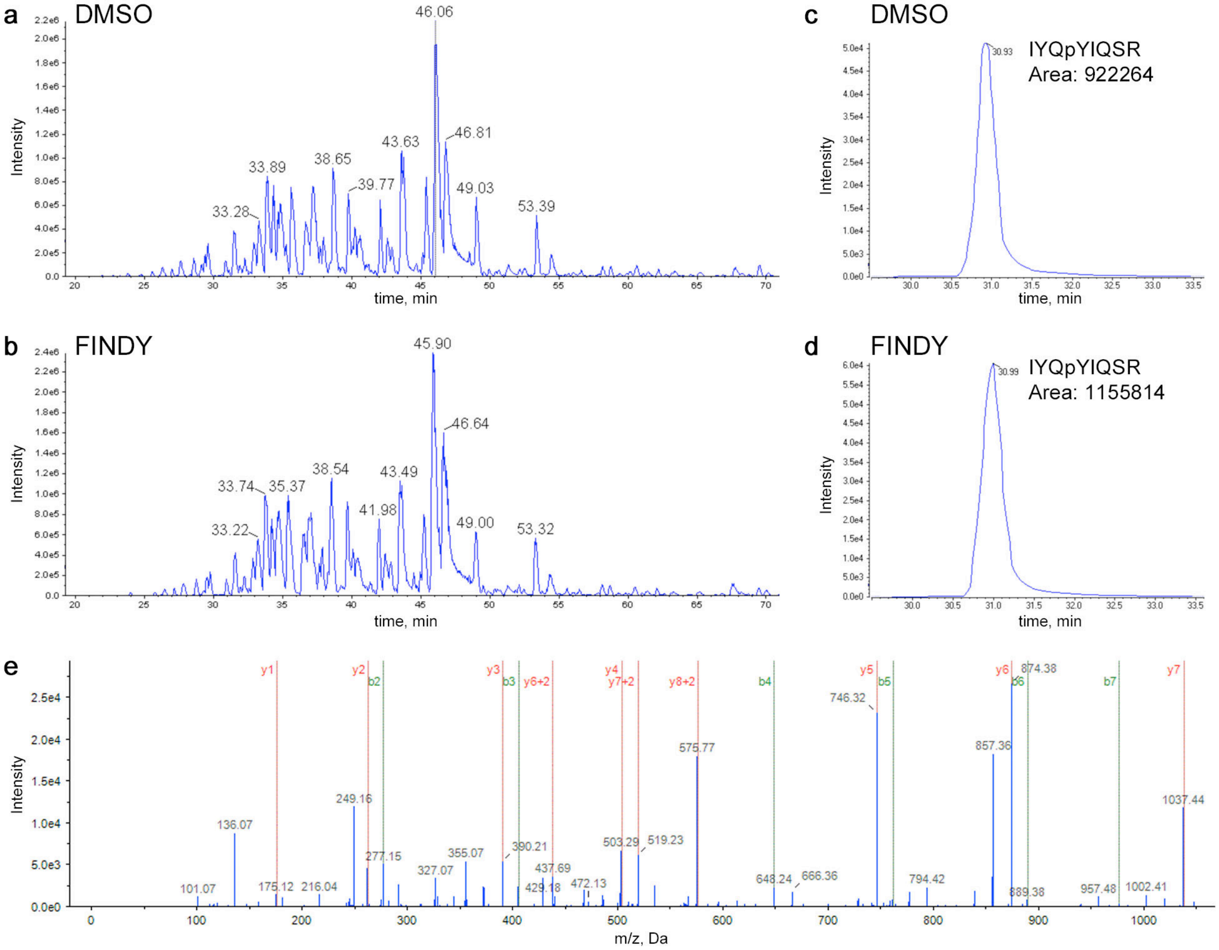

f

Residue	b	b+2	y	y+2
I	114.09	57.549	1150.5	575.77
Y	277.15	139.08	1037.4	519.23
Q	405.21	203.11	874.38	437.69
phospho-Y	648.24	324.63	746.32	373.67
I	761.33	381.17	503.29	252.15
Q	889.39	445.2	390.21	195.61
S	976.42	488.71	262.15	131.58
R	1132.5	566.76	175.12	88.063

Supplementary Figure 5: Detection of activation-loop autophosphorylation in DYRK1A
Expression of FLAG-DYRK1A was induced by doxycycline and epoxomicin for 5 h in the presence or absence of FINDY (10 μM). The HEK293 cell lysates containing FLAG-DYRK1A were incubated with anti-FLAG antibody-conjugated beads and the immunocomplexes were subjected to elution with glycine buffer (pH 3.0) followed by neutralization. The eluted proteins were treated with trypsin, and the digested peptides were analysed by LC-MS/MS. Peptides and sites of phosphorylation were identified from the peptide mass and the fragmentation pattern following collision-induced dissociation.

(**a** and **b**) Total ion chromatograms of the trypsin-digested peptides from the FLAG-DYRK1A proteins produced in the (**a**) absence or (**b**) presence of FINDY. Nearly identical quantities of the peptides were observed between (**a**) and (**b**).

(**c** and **d**) Selected ion chromatograms of the tryptic phosphopeptide IYQY*IQSR from FLAG-DYRK1A produced in the (**c**) presence or (**d**) absence of FINDY. Y* represents phospho-Tyr321. FINDY did not affect the amount of the phosphopeptide. The non-phosphorylated peptide IYQYIQSR was not detected in the FLAG-DYRK1A proteins produced in the presence or absence of FINDY (data not shown).
(**e** and **f**) LC-MS/MS spectrum with the detailed peak assignment of the tryptic phosphopeptide IYQY*IQSR.
Representative data from the duplicate experiments is shown.

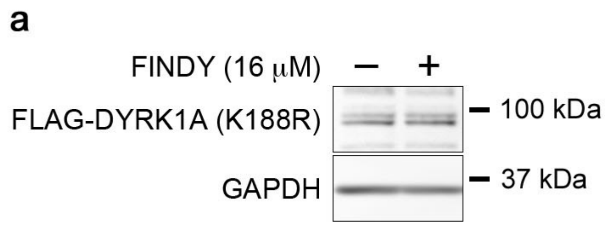

Supplementary Figure 6: FINDY does not decrease the amount of DYRK1A with the K188R and S97A mutations

(**a** and **b**) FINDY was added at 16 μM with doxycycline to HEK293 cells harboring the expression vectors of FLAG-DYRK1A with K188R (**a**) or S97A (**b**), which were stably integrated in the genome. After incubation for 16 h, total cell lysates were subjected to SDS-PAGE followed by Western blot analysis using the corresponding antibodies against FLAG and GAPDH. Representative data from the duplicate experiments is shown.

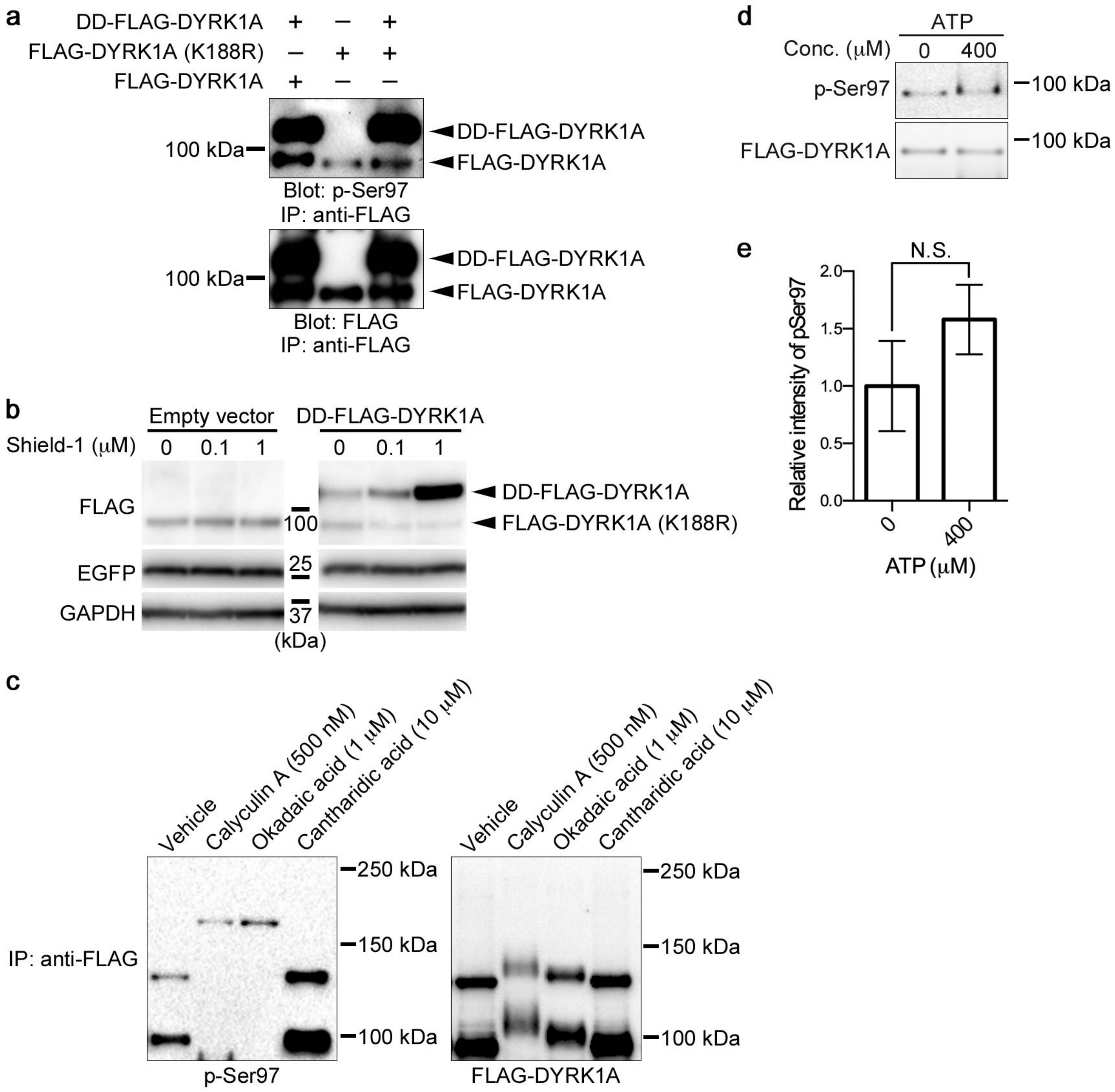

Supplementary Figure 7: Characterization of Ser97 autophosphorylation

(**a**) Intramolecular autophosphorylation of Ser97 in DYRK1A. DYRK1A was expressed as a fusion protein with DD and FLAG tags (DD-FLAG-DYRK1A). Bicistronic expression of the kinase-dead mutant of FLAG-DYRK1A (K188R) with EGFP was driven by doxycycline. HEK293 cells harbouring the expression vectors of DD-FLAG-DYRK1A and intact FLAG-DYRK1A or mutant FLAG-DYRK1A (K188R) were incubated with Shield-1 for 16 h to allow for the stabilization of DD-FLAG-DYRK1A. After accumulation of DD-FLAG-DYRK1A, FLAG-DYRK1A or FLAG-DYRK1A (K188R) were expressed for 5 h in the presence of Shield-1, doxycycline, and epoxomicin. The HEK293 cell lysates were then incubated with anti-FLAG antibody-conjugated beads and the immunocomplexes were subjected to Western

blot analysis using antibodies against p-Ser97 and FLAG. If Ser97 autophosphorylation were mediated by an intermolecular mechanism, then Ser97 of the kinase-dead K188R mutant would be phosphorylated when expressed in the presence of DD-FLAG-DYRK1A. However, in spite of the excess amount of DD-FLAG-DYRK1A, the phosphorylation of Ser97 in the kinase-dead mutant did not increase compared to results obtained in the absence of DD-FLAG-DYRK1A. This suggests Ser97 autophosphorylation of DYRK1A is catalysed in an intramolecular manner. Representative data from the triplicate experiments is shown.

(**b**) HEK293 cells harbouring the empty vector or the expression vector of DD-FLAG-DYRK1A with the bicistronic expression cassette of the kinase-dead FLAG-DYRK1A (K188R) and EGFP were incubated with Shield-1 at the indicated concentrations for 16 h. After accumulation of DD-FLAG-DYRK1A, FLAG-DYRK1A (K188R) was expressed for 16 h in the presence of Shield-1 and doxycycline. The kinase-dead FLAG-DYRK1A (K188R) did not increase in the presence of DD-FLAG-DYRK1A, supporting the conclusion in (**a**). Representative data from the triplicate experiments is shown.

(**c**) p-Ser97 is susceptible to dephosphorylation in HEK293 cells. The HEK293 cells were treated with the indicated concentration of calyculin A, okadaic acid, and cantharidic acid in the presence of doxycycline for 8 h. The total cell lysates were subjected to immunoprecipitation using antibody against FLAG-tag. The immuno-precipitated FLAG-DYRK1A was detected using antibodies against p-Ser97 and FLAG-tag. Western blot analysis showed that the treatment with cantharidic acid increased the p-Ser97 signal. In contrast, the treatment with calyculin A and okadaic acid did not increase the p-Ser97 signal, but shifted the band of FLAG-DYRK1A, which may indicate that calyculin A and okadaic acid inhibited dephosphorylation of other sites in DYRK1A. Although these three compounds commonly inhibited PP1 and PP2A, protein phosphatases inhibited by these compounds have not been completely identified. Phosphatase(s) inhibited by cantharidic acid may play a role in dephosphorylation of p-Ser97 in the cells. Representative data from the triplicate experiments is shown.

(**d**) FLAG-DYRK1A was produced in HEK293 cells for 16 h, then purified with anti-FLAG antibody-conjugated beads. These beads were incubated at 37 ºC for 2 h in the presence or absence of ATP (400 μM). Each protein was detected with the corresponding antibodies against p-Ser97 or FLAG. Representative data from the duplicate experiments is shown.

(**e**) The band intensities in (**d**) were quantified. The graph shows the means +/– SD (n=4). N.S. indicates not significant. Statistical analysis was performed with the Mann–Whitney U test.

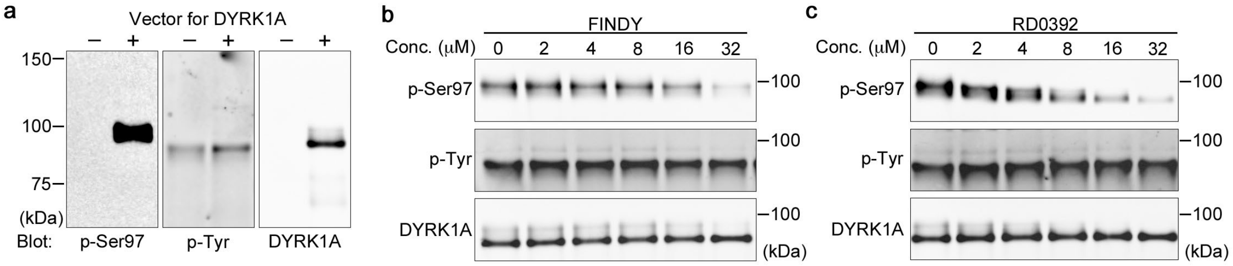

Supplementary Figure 8: *In vitro* coupled transcription-translation of DYRK1A

(**a**) FLAG-DYRK1A expressed in the *in vitro* coupled transcription-translation system was immunopurified with anti-FLAG antibody conjugated beads. The labelled proteins were blotted with the corresponding antibodies against p-Ser97, p-Tyr, or FLAG. Representative data from the triplicate experiments is shown.

(**b** and **c**) FLAG-DYRK1A was expressed in the *in vitro* system in the presence of (**b**) FINDY or (**c**) RD0392. The expressed proteins were immunopurified, then subjected to SDS-PAGE followed by Western blot analysis. Representative data from the triplicate experiments is shown.

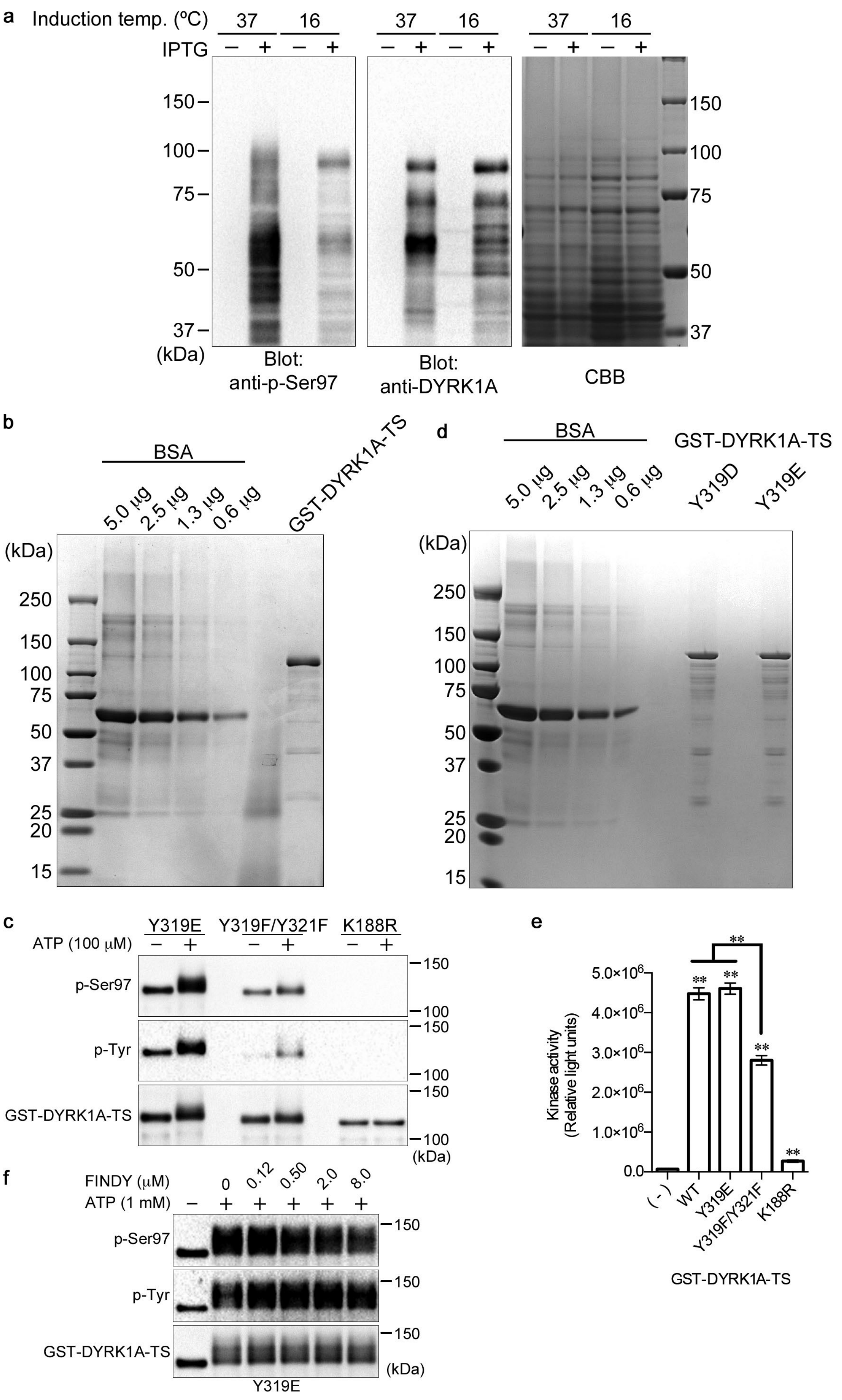

a
Induction temp. (ºC)
37
16
IPTG
Blot: anti-p-Ser97
Blot: anti-DYRK1A
CBB
(kDa)
b
BSA
5.0 µg
2.5 µg
1.3 µg
0.6 µg
GST-DYRK1A-TS
c
Y319E
Y319F/Y321F
K188R
ATP (100 µM)
p-Ser97
p-Tyr
GST-DYRK1A-TS
d
GST-DYRK1A-TS
Y319D
Y319E
e
Kinase activity (Relative light units)
(-)
WT
Y319E
Y319F/Y321F
K188R
GST-DYRK1A-TS
f
FINDY (µM)
ATP (1 mM)
Y319E

Supplementary Figure 9: Development of the *in vitro* autophosphorylation assay

(**a**) Incubation of transformed *E. coli* cells with the expression vector for DYRK1A at 16 ºC suppressed the Ser97 autophosphorylation of the recombinant DYRK1A protein. Rosetta(DE3)pLys cells were transformed with pET28a(+)-His-DYRK1A and cultured at 30 ºC. Expression of His-DYRK1A was induced by the addition of IPTG at 37 ºC or 16 ºC. Total cell lysates were subjected to SDS-PAGE, followed by Western blot analysis and staining with CBB. Overall, the p-Ser97 signals of the recombinant His-DYRK1A proteins produced at 16 ºC were weaker than those produced at 37 ºC. Blotting with anti-DYRK1A antibody showed partial fragments of His-DYRK1A, which prompted us to purify the recombinant DYRK1A using tandem affinity purification with Twin-Strep-tag (TS) followed by GST-tag.

(**b** and **d**) Recombinant GST-DYRK1A-TS protein (wild-type in **b**, and Y319D and Y319E in **d**) was prepared via tandem affinity purification, separated by SDS-PAGE, then stained with CBB. BSA was also loaded to measure the amount of protein.

(**c**) The Y319, Y319F/Y321F, and K188R mutants producing recombinant GST-DYRK1A-TS protein at 6 ºC were reacted with ATP (100 μM) for 2 h. The resulting proteins were analysed by SDS-PAGE followed by Western blot. Representative data from the duplicate experiments is shown.

(**e**) The recombinant DYRK1A protein and its mutants (Y319E, Y319F/Y321F, and K188R), which were produced in *E. coli*, were subjected to an *in vitro* kinase assay. The proteins bound on GST-Accept resin were reacted with the substrate peptide in the presence of 100 μM of ATP for 2 h. Kinase activities were measured by ADP-Glo kinase assay kit (see Methods). Statistical significance was calculated compared with no DYRK1A protein (–) and also calculated between WT or Y319E, and Y319F/Y321F. $^{**}p<0.05$, n=6. Statistical analysis was performed with the Mann–Whitney U test.

(**f**) The Y319E mutant was reacted with ATP (1 mM) in the presence of FINDY. Representative results are shown. Quantitative data is shown in **Figure 6d**.

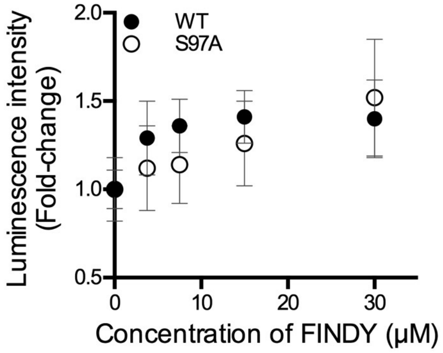

Supplementary Figure 10: FINDY strengthens the interaction between DYRK1A with the S97A mutation and the co-chaperone CDC37.

293T cells stably expressing CDC37-nanoKAZ were transiently transfected with an expression vector of 3xFLAG-DYRK1A (WT) and the S97A mutant. Twenty-four hours after the transfection, cells were treated with FINDY, and incubated for an additional 24 h. Luminescence intensities are shown as fold-changes relative to that at 0 μM, normalized to the amount of 3xFLAG-DYRK1A (WT) and the S97A mutant bound on a 96-well plate. Points on the graph are means +/– SD (n=8). There is no significant difference between WT and S97A at the same concentration of FINDY. Statistical analysis was performed with the Mann–Whitney U test. Representative data from the duplicate experiments is shown.

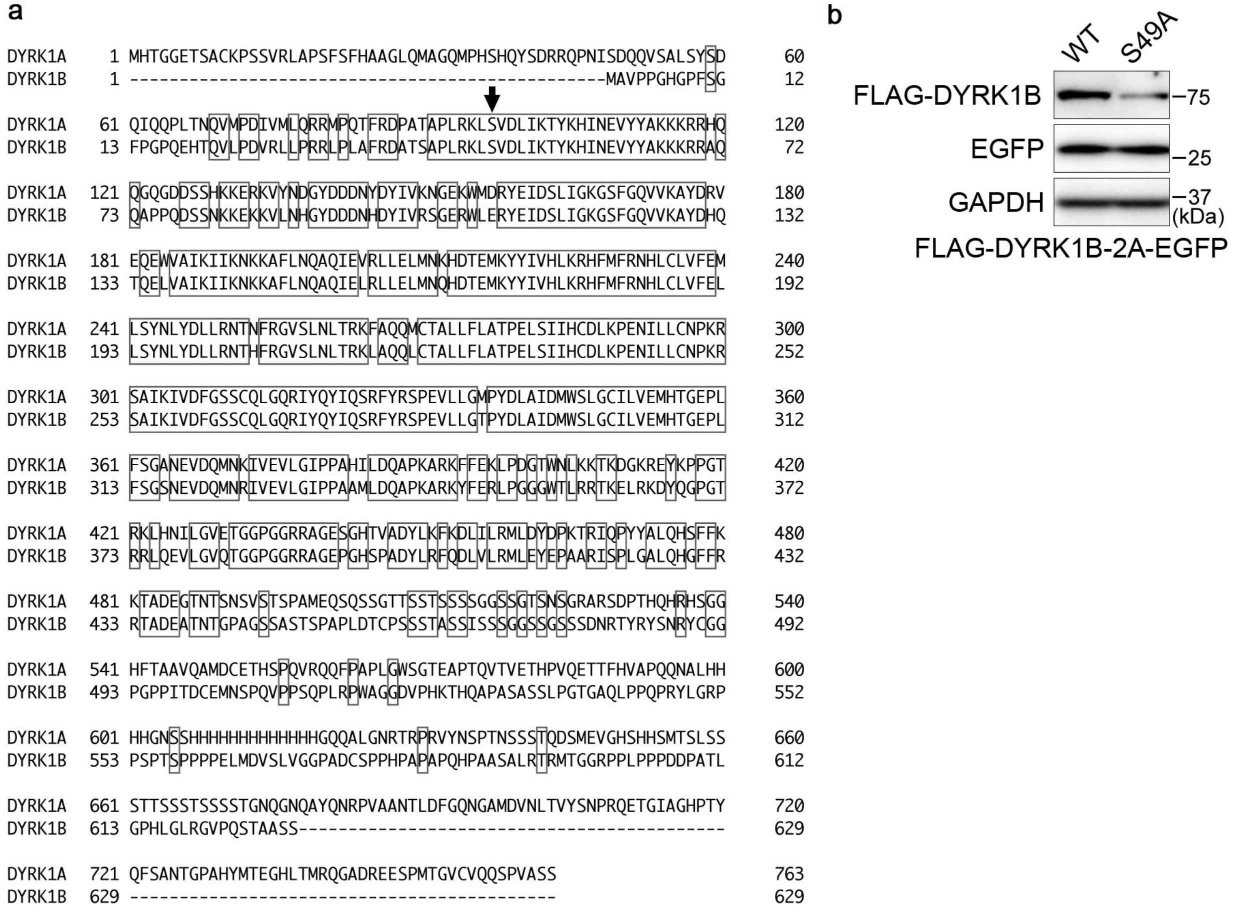

Supplementary Figure 11: Autophosphorylation of Ser49 in DYRK1B is also required for protein stability

(**a**) The amino acid sequences of *Homo sapiens DYRK1A* (NM_001396) and *DYRK1B* (BC018751) are shown. Identical residues are indicated in the rectangular boxes. An arrow indicates the conserved serine residues corresponding to Ser97 in DYRK1A and to Ser49 in DYRK1B.

(**b**) Substitution of Ser49 to Ala (S49A) in DYRK1B destabilized the mutant protein, although the bicistronic expression of EGFP was not affected. Representative data from the triplicate experiments is shown.

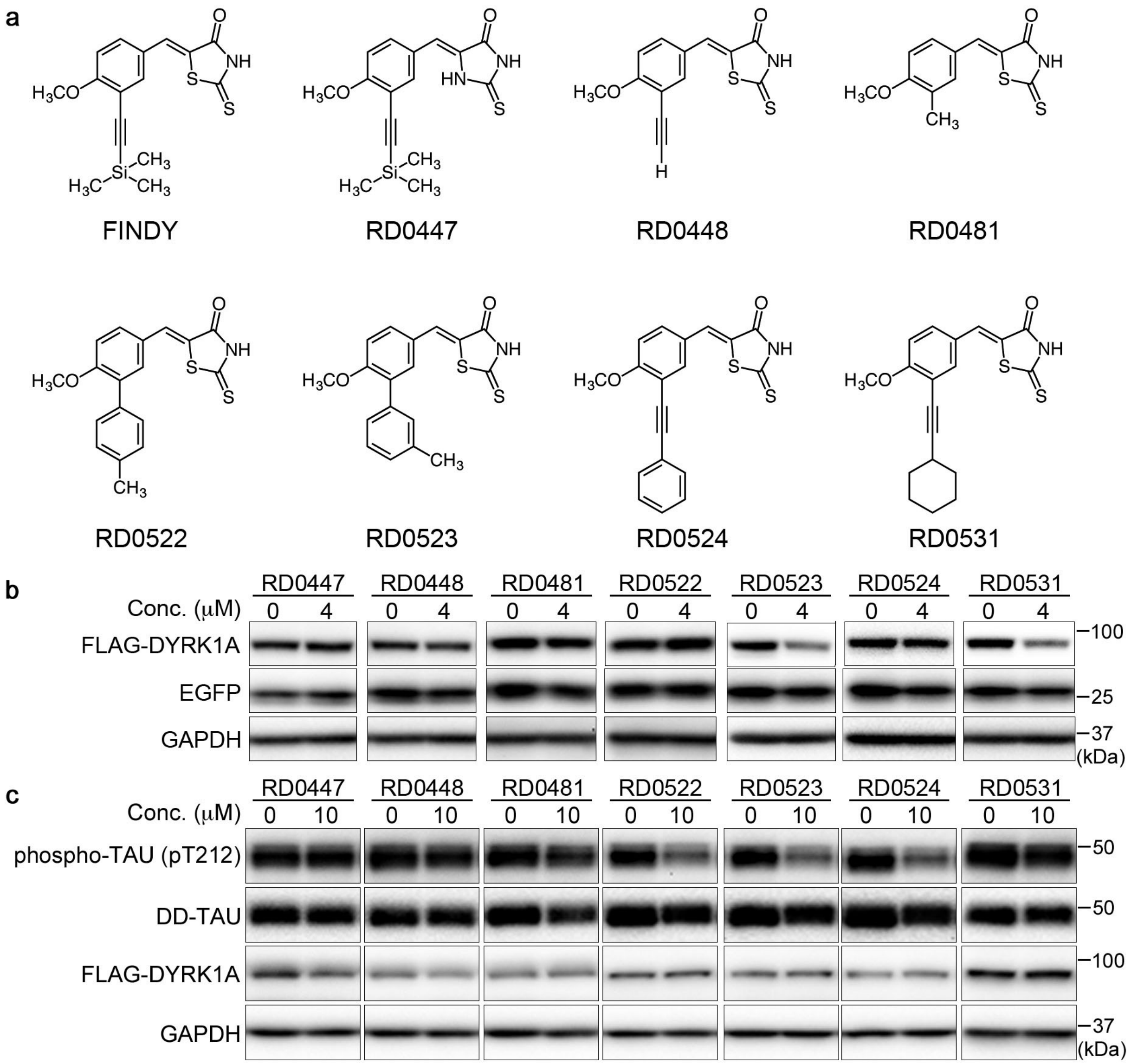

Supplementary Figure 12: Cell-based assays using structural derivatives of FINDY

(**a**) Structures of FINDY derivatives.

(**b**) Destabilization of DYRK1A by FINDY derivatives. FINDY derivatives were added with doxycycline to HEK293 cells harbouring the bicistronic expression vector. After incubation for 5 h, total cell lysates were subjected to SDS-PAGE, followed by Western blot analysis using antibodies against FLAG, EGFP, and GAPDH. Representative data from the duplicate experiments is shown.

(**c**) The effects of FINDY derivatives on the TAU phosphorylation catalysed by mature DYRK1A. The cells were incubated with FINDY derivatives according to Tc 2, which is shown in **Figure 1a**. Representative data from the duplicate experiments is shown.

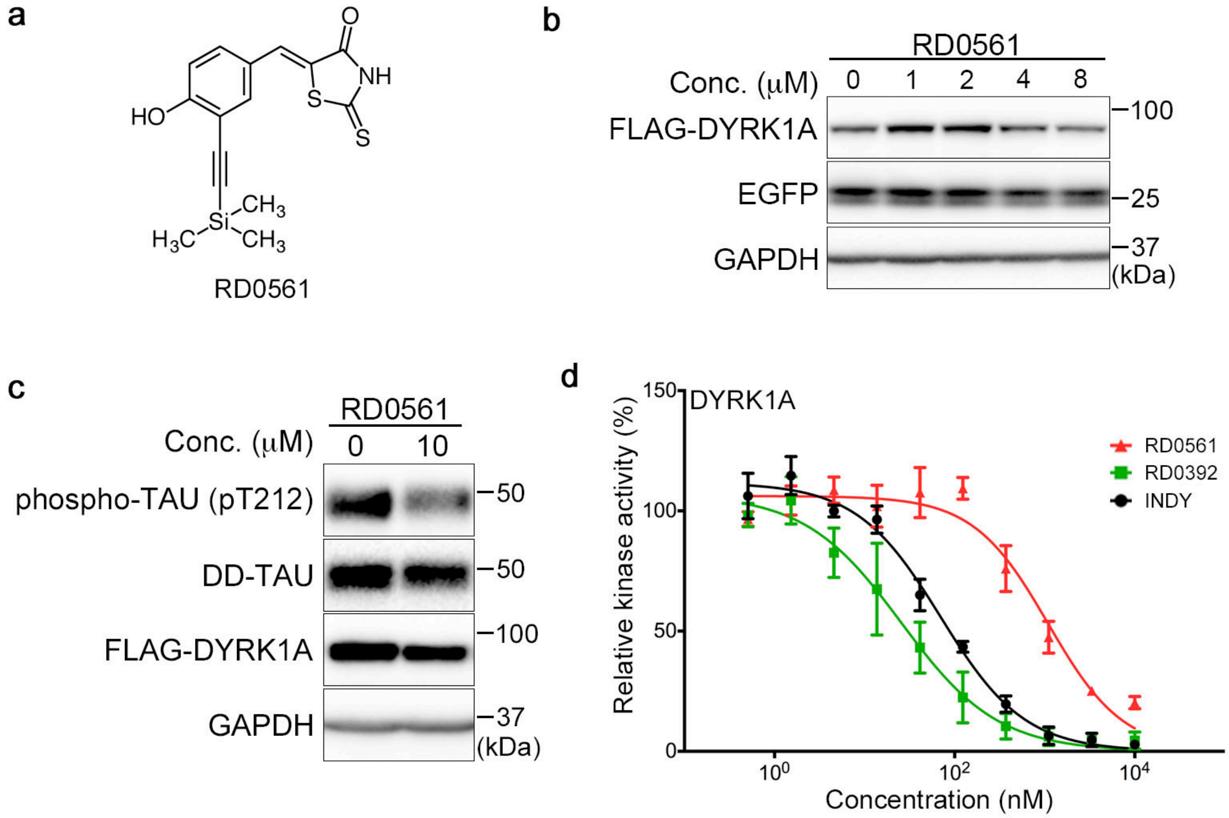

Supplementary Figure 13: The methoxyl moiety at the C4 position in FINDY underlies the intermediate-selective inhibition

(**a**) Structure of RD0561.

(**b**) RD0561 did not destabilize DYRK1A. RD0561 was added with doxycycline to HEK293 cells harbouring the bicistronic expression vector. After incubation for 5 h, total cell lysates were subjected to SDS-PAGE followed by Western blot analysis using antibodies against FLAG, EGFP, and GAPDH. Representative data from the triplicate experiments is shown.

(**c**) RD0561 inhibited the TAU phosphorylation catalysed by mature DYRK1A. The cells were incubated with FINDY derivatives in Tc 2, as shown in **Figure 1a**. Representative data from the duplicate experiments is shown.

(**d**) Recombinant DYRK1A was incubated with the peptide substrate DYRKtide in the presence of different concentrations of INDY, RD0392, or RD0561. INDY, RD0392, and RD0561 inhibited kinase activity with IC_{50} values of 70.0 nM, 25.6 nM, and 1.09 μM, respectively. Representative curves are shown. The results are presented as means +/– SD (n=3).

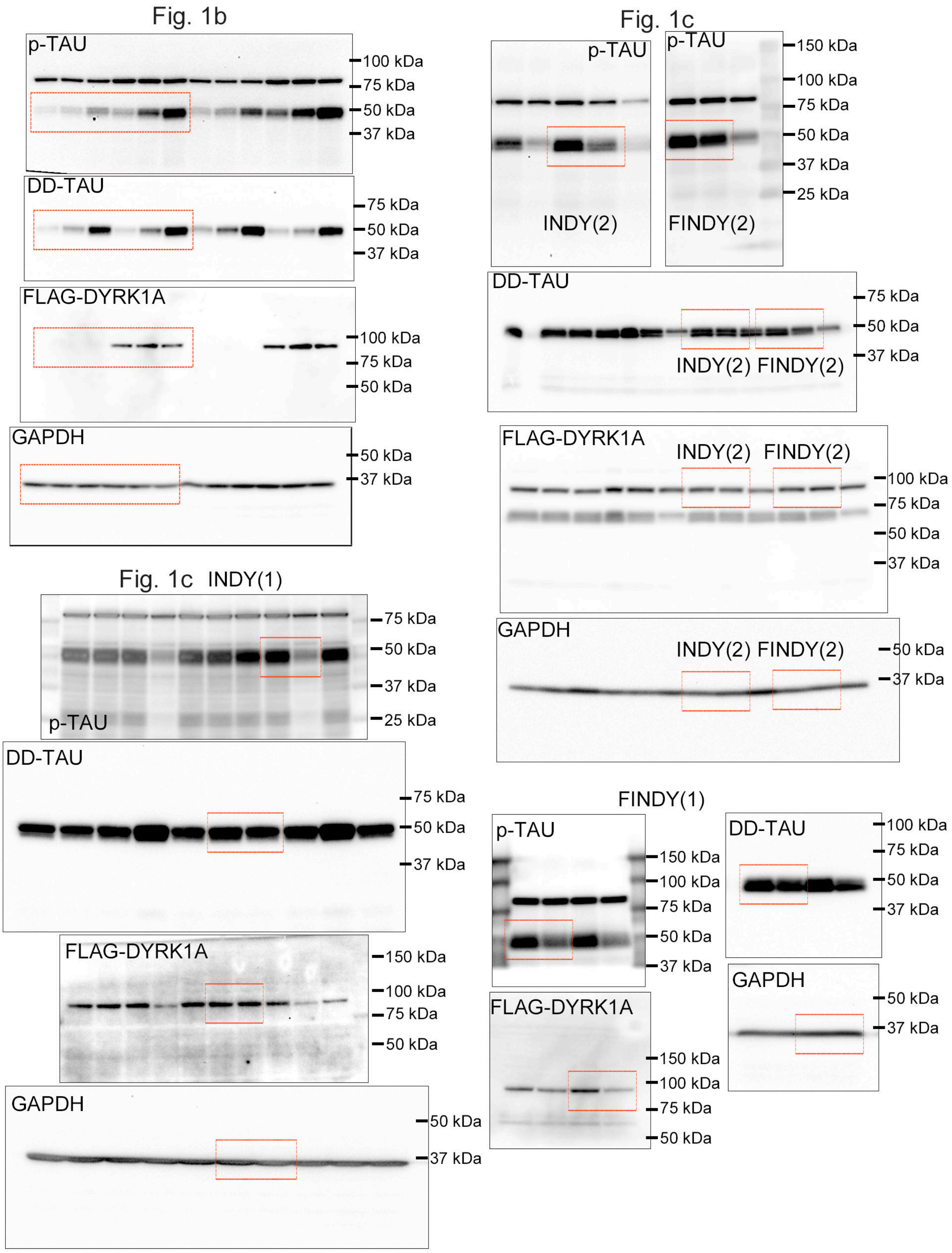

Supplementary Figure 14: Full gel images

Fig. 2b

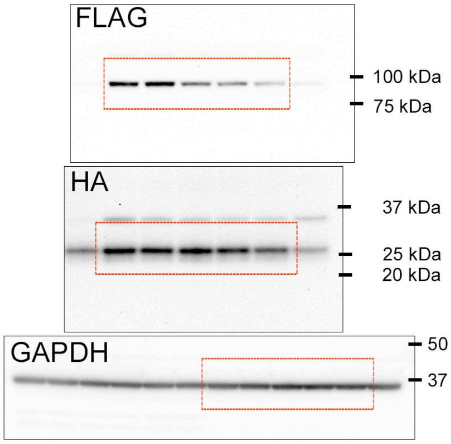

Fig. 2d

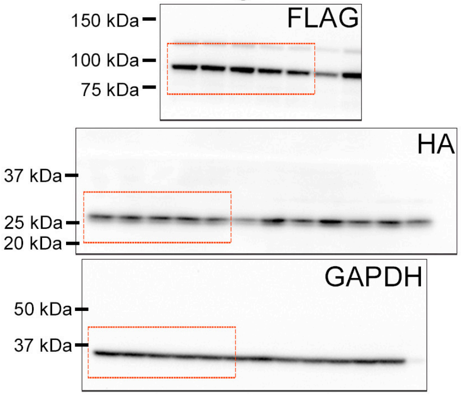

Fig. 2c

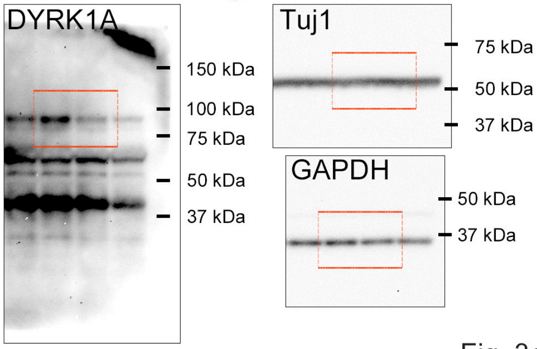

Fig. 3a

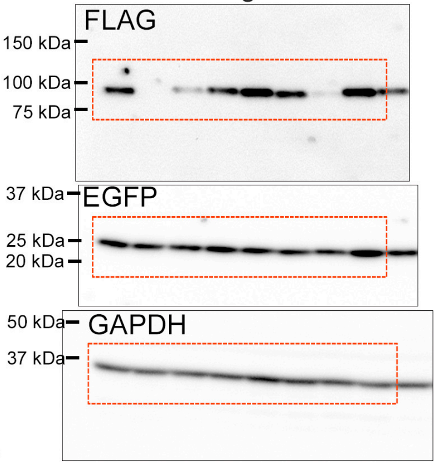

Fig. 3c

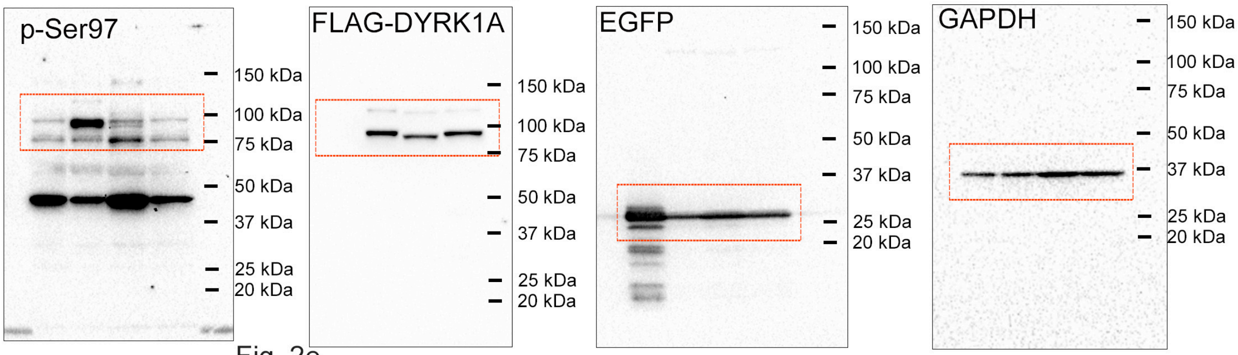

Fig. 2e

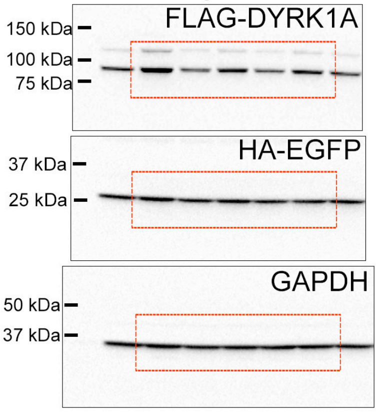

Fig. 3d

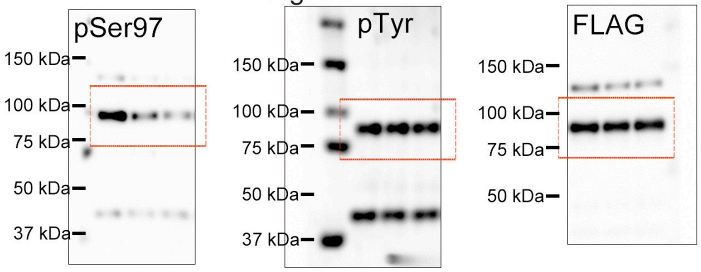

Fig. 4b

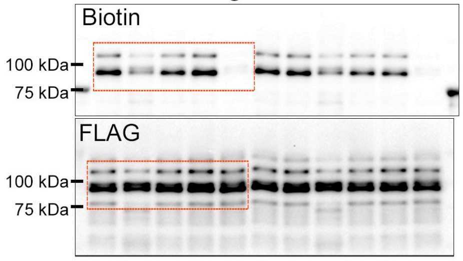

Fig. 6a

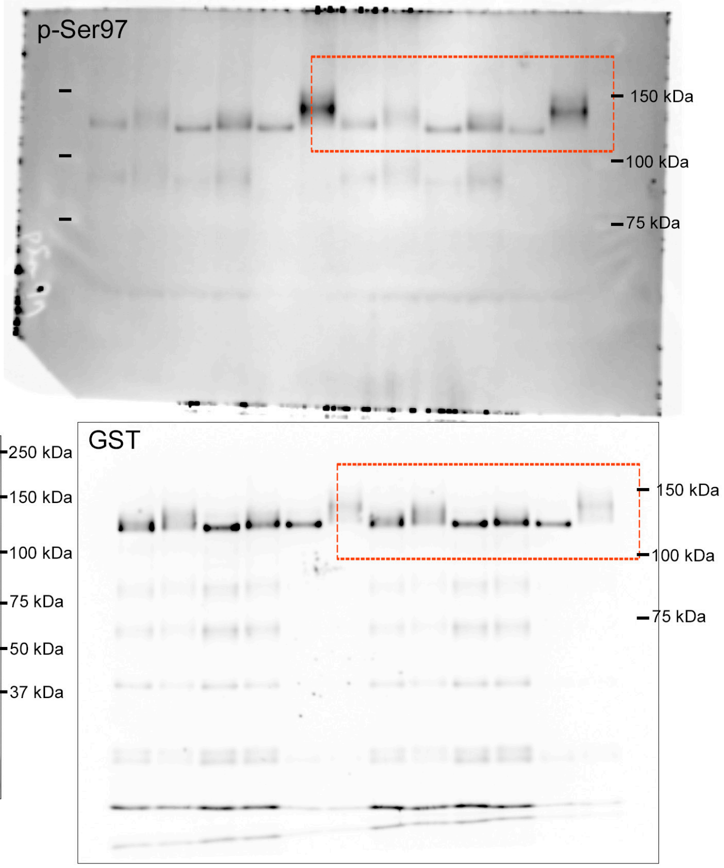

Fig. 6c

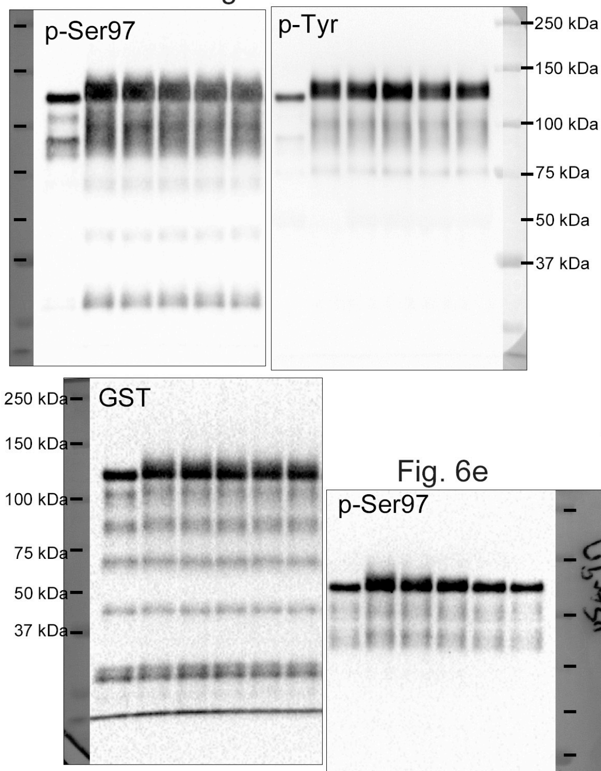

Fig. 6e

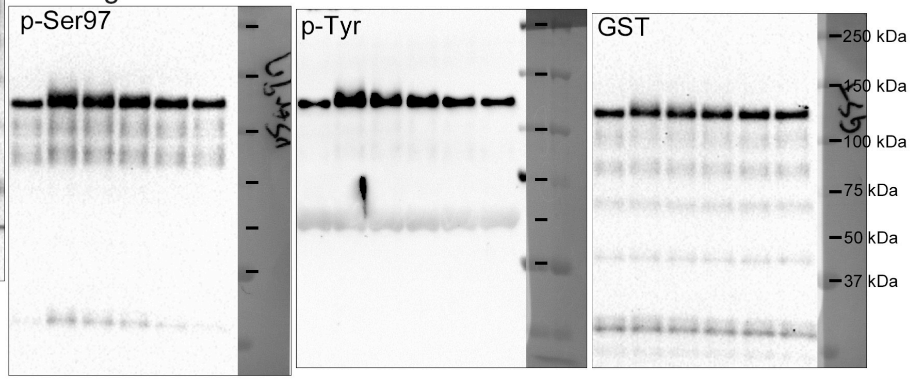

Fig. 5a

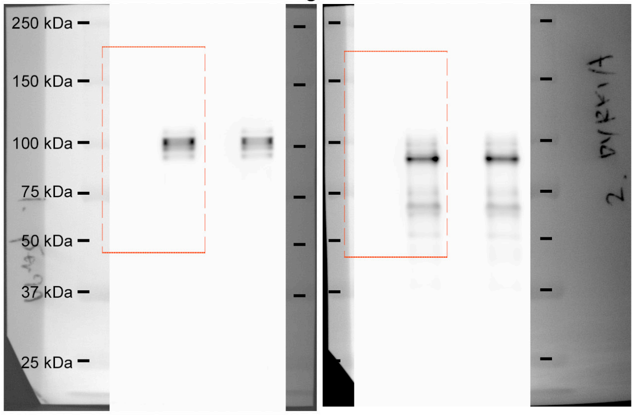

Fig. 5f

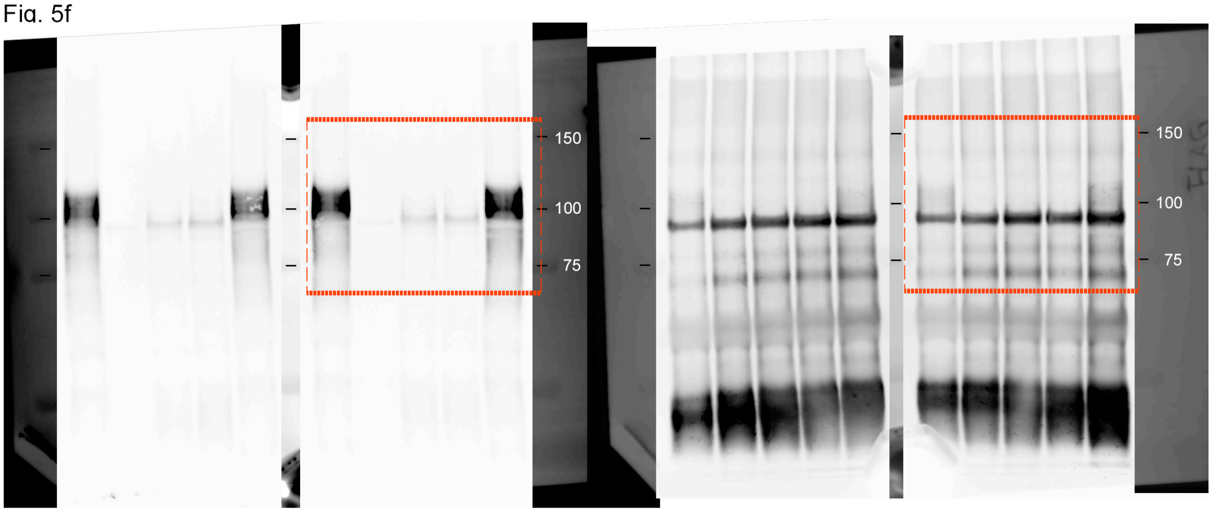

Fig. 5b

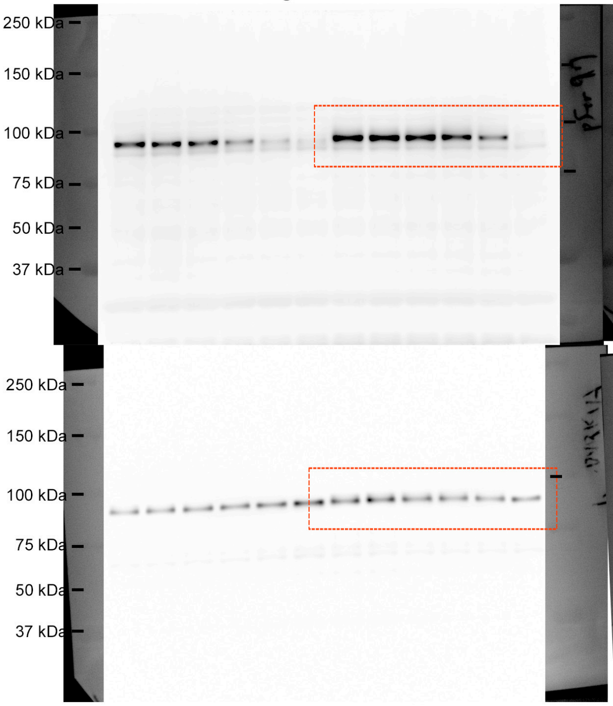

Fig. 5c

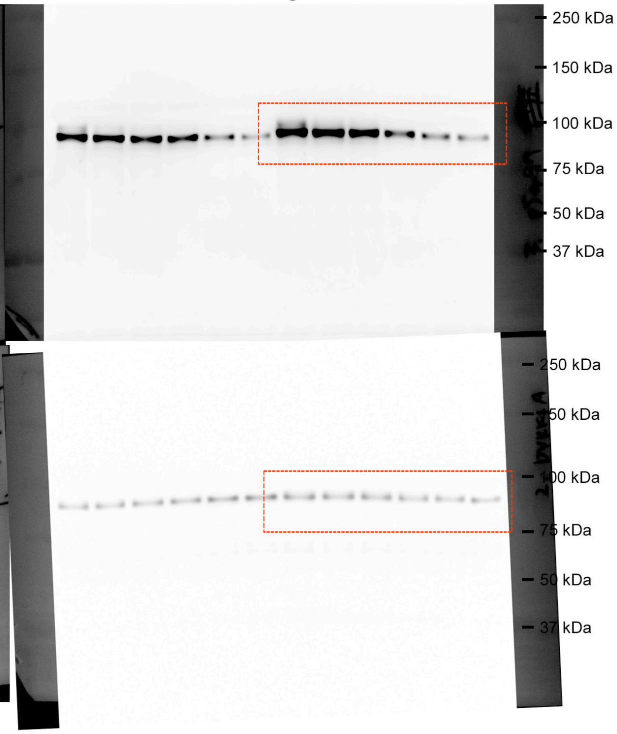

Fig. 8c,d

Fig. 8e

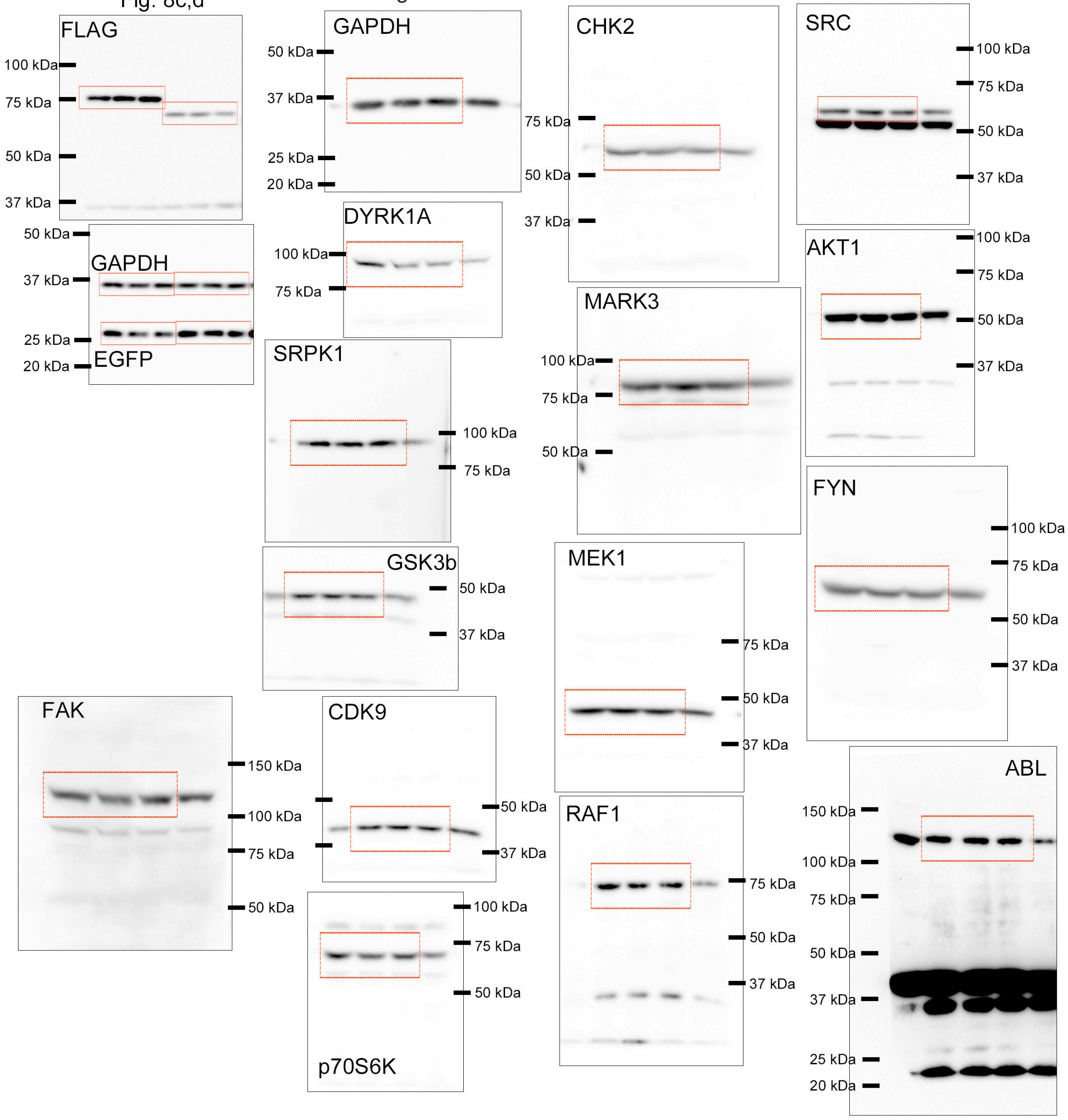

Supplementary Fig. 1

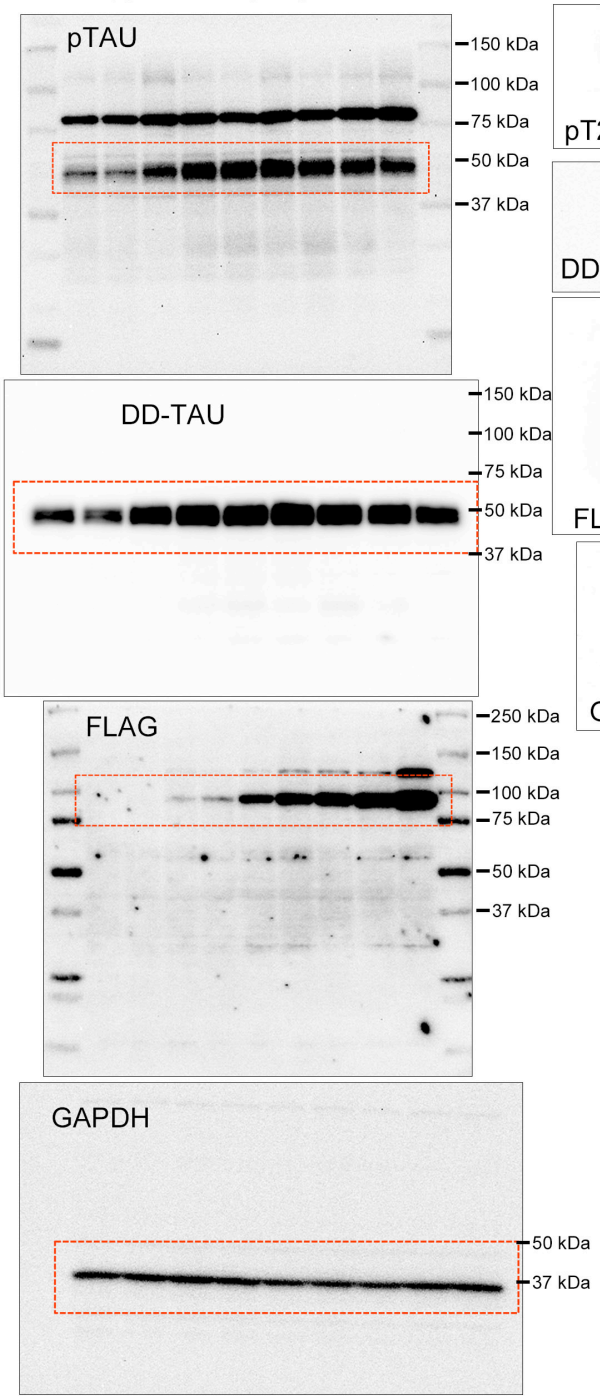

Supplementary Fig. 2b

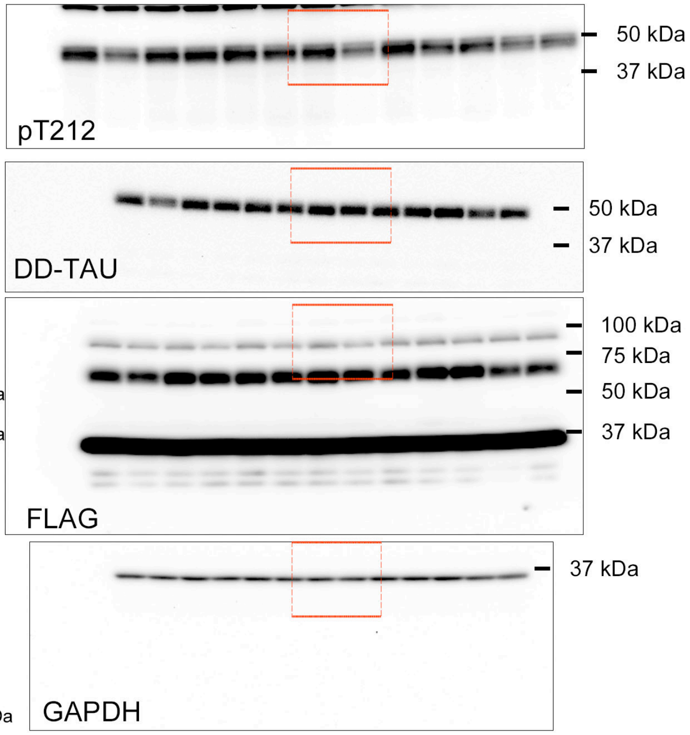

Supplementary Fig. 4

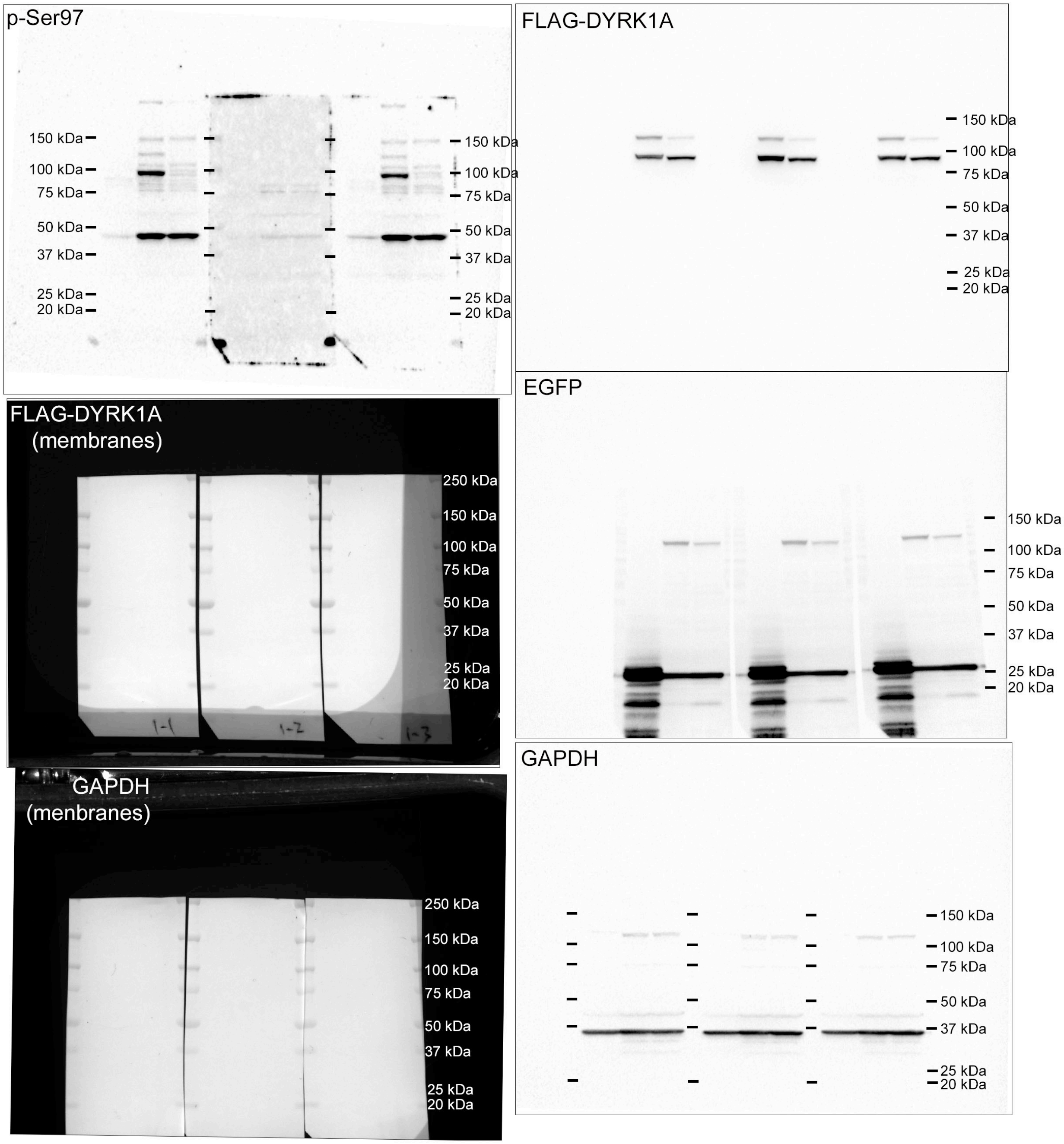

Supplementary Fig. 6a

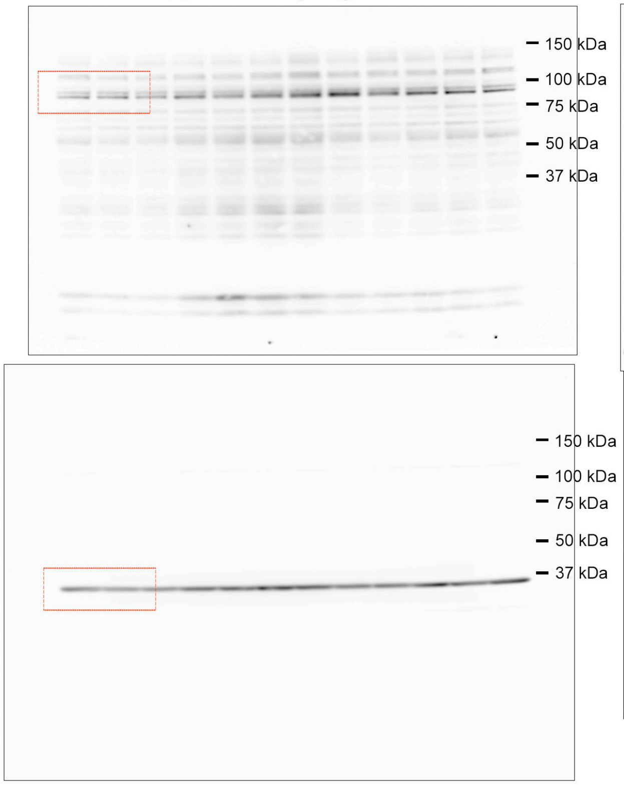

Supplementary Fig. 6b

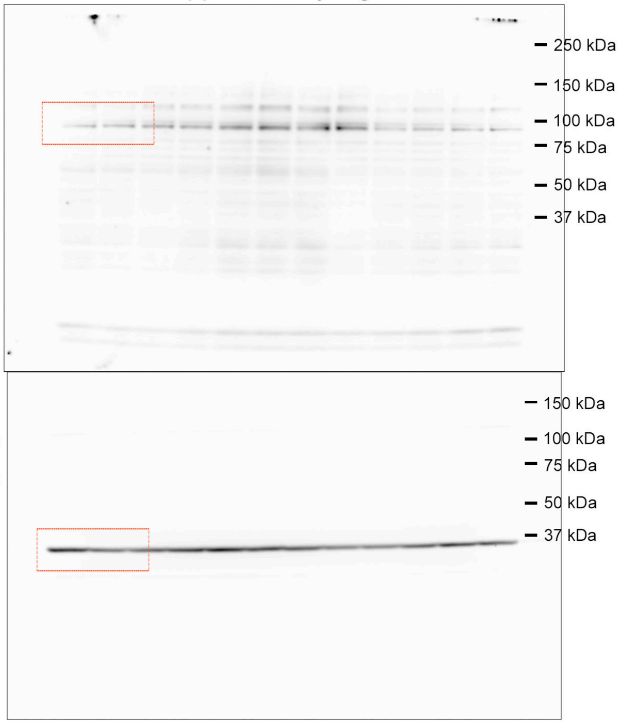

Supplementary Figure 7a

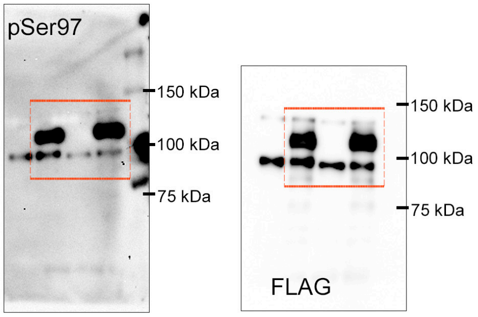

Supplementary Figure 7b

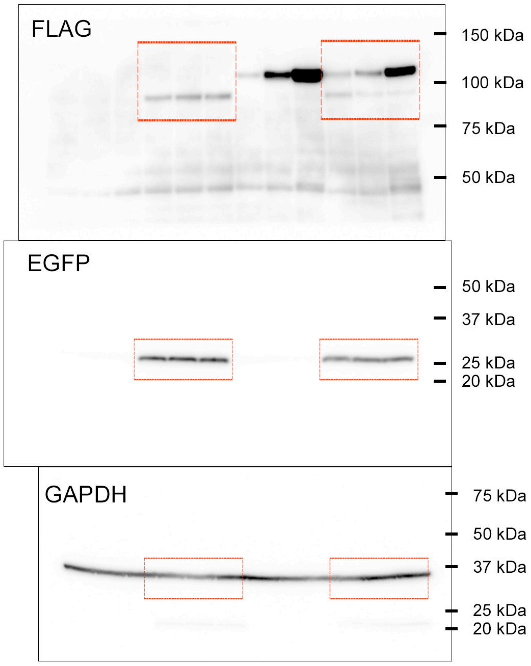

Supplementary Figure 7c

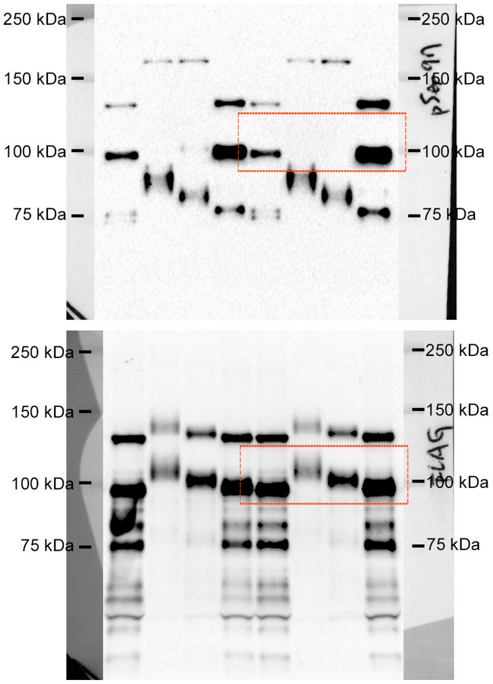

Figure 3e

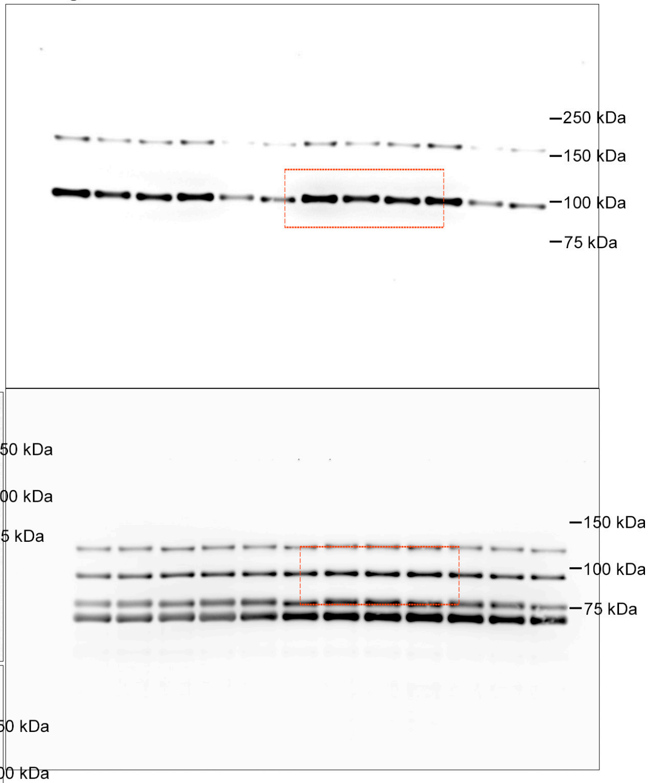

Supplementary Figure 7d

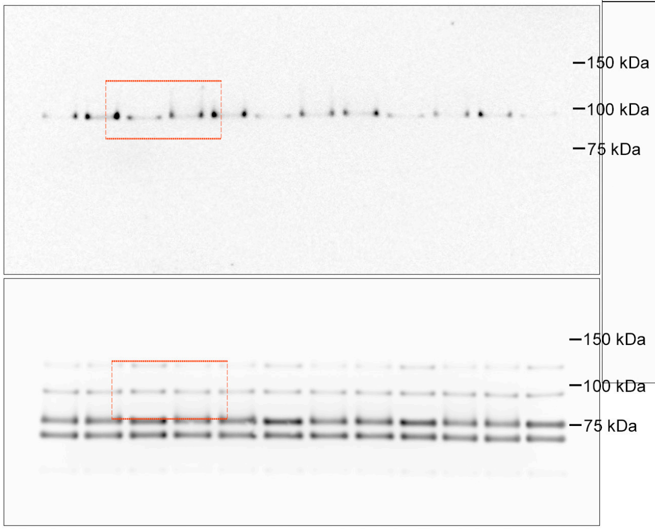

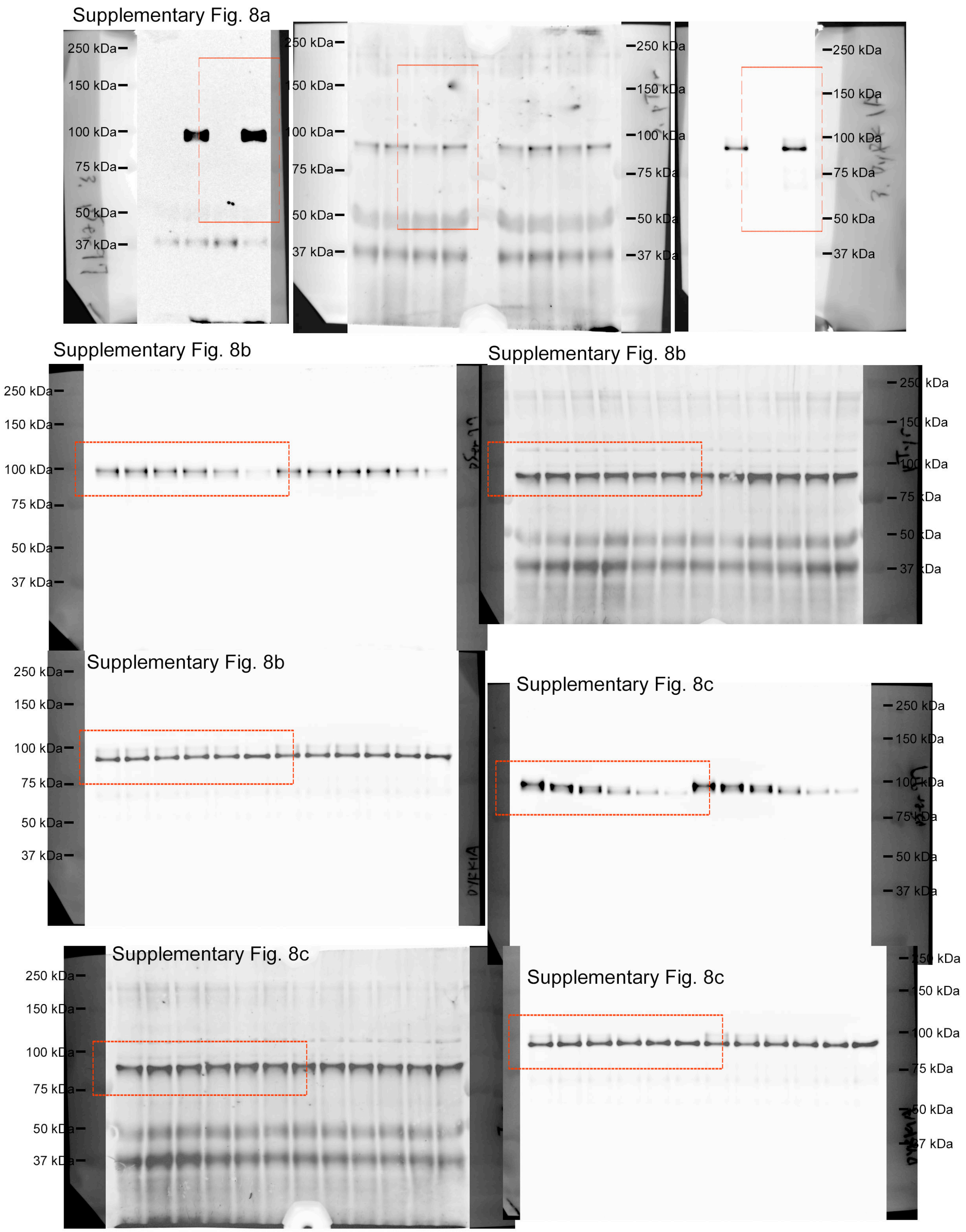
Supplementary Fig. 8a
250 kDa
150 kDa
100 kDa
75 kDa
50 kDa
37 kDa
Supplementary Fig. 8b
250 kDa
150 kDa
100 kDa
75 kDa
50 kDa
37 kDa
Supplementary Fig. 8b
250 kDa
150 kDa
100 kDa
75 kDa
50 kDa
37 kDa
Supplementary Fig. 8b
Supplementary Fig. 8c
Supplementary Fig. 8c
Supplementary Fig. 8c

Supplementary Fig. 9c

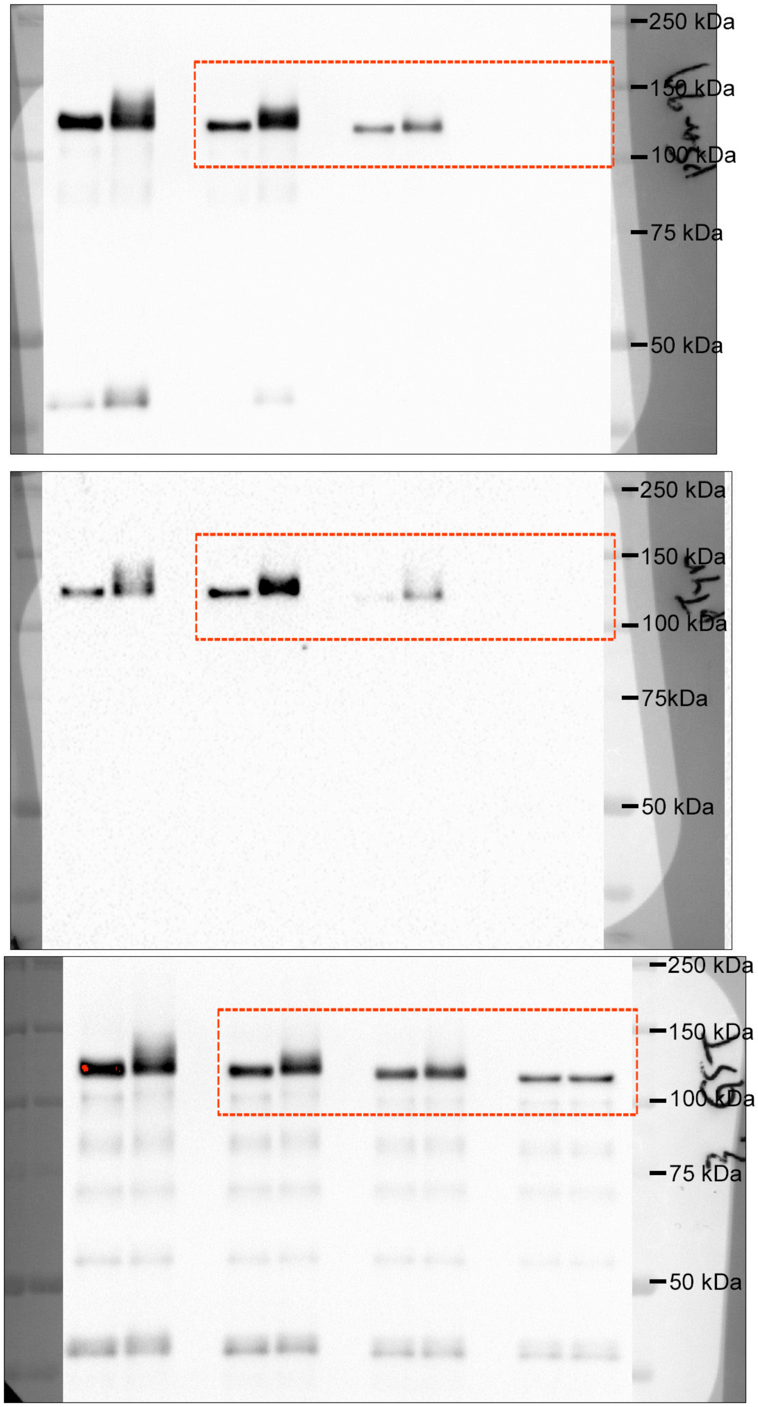

Supplementary Fig. 9f

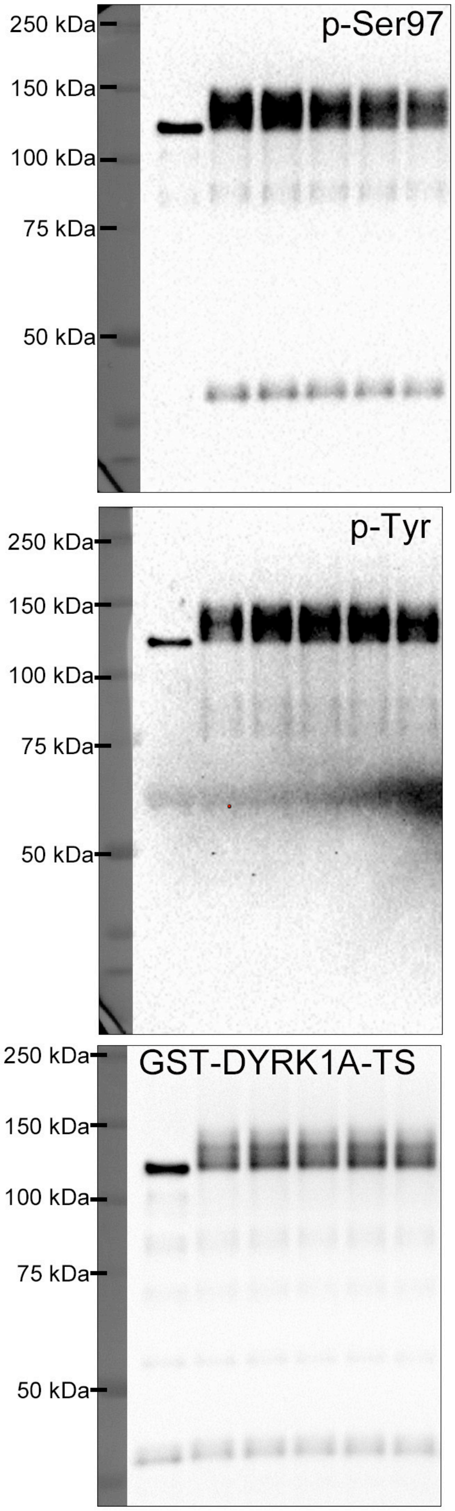

Supplementary Fig. 12b

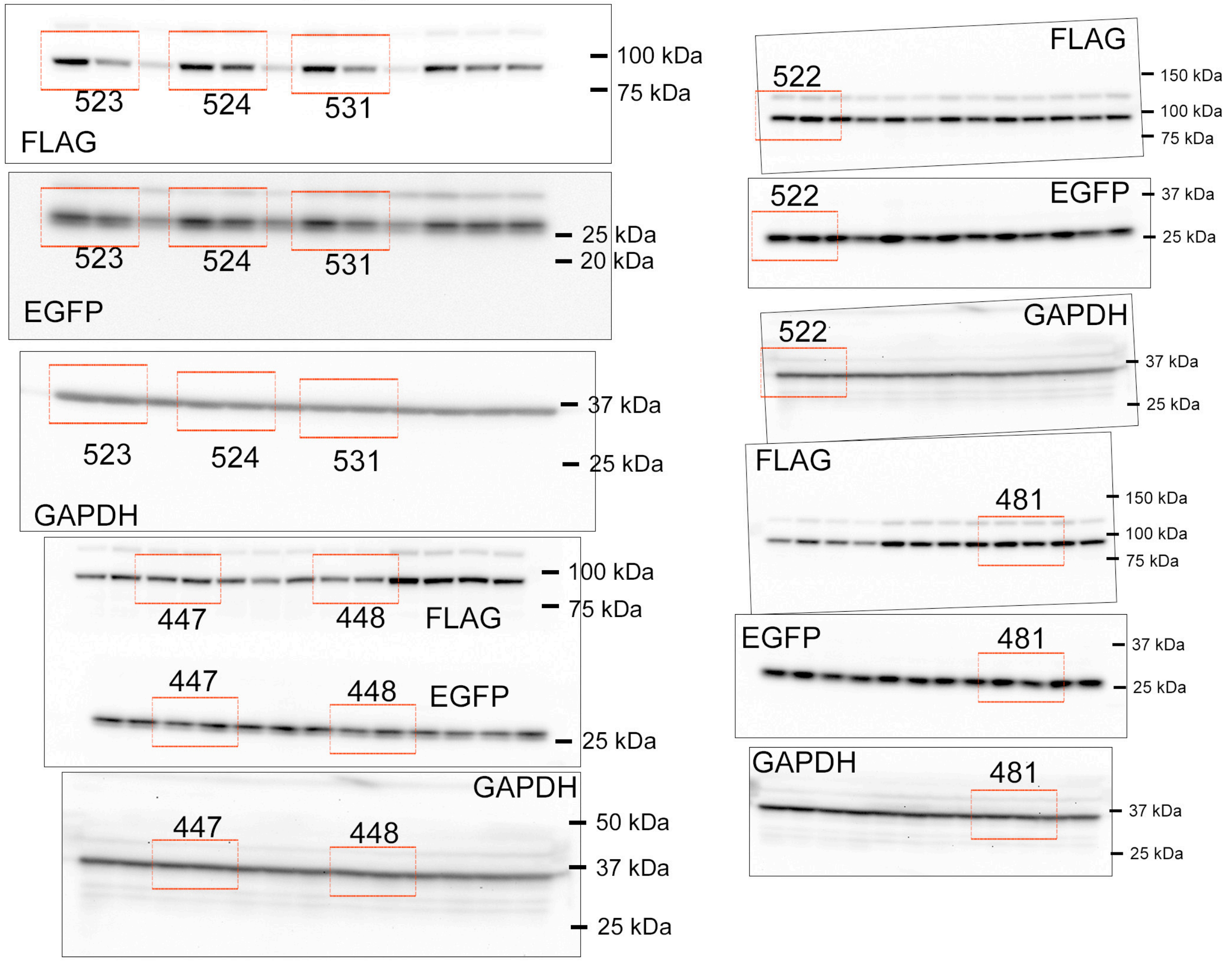

Supplementary Fig. 12c

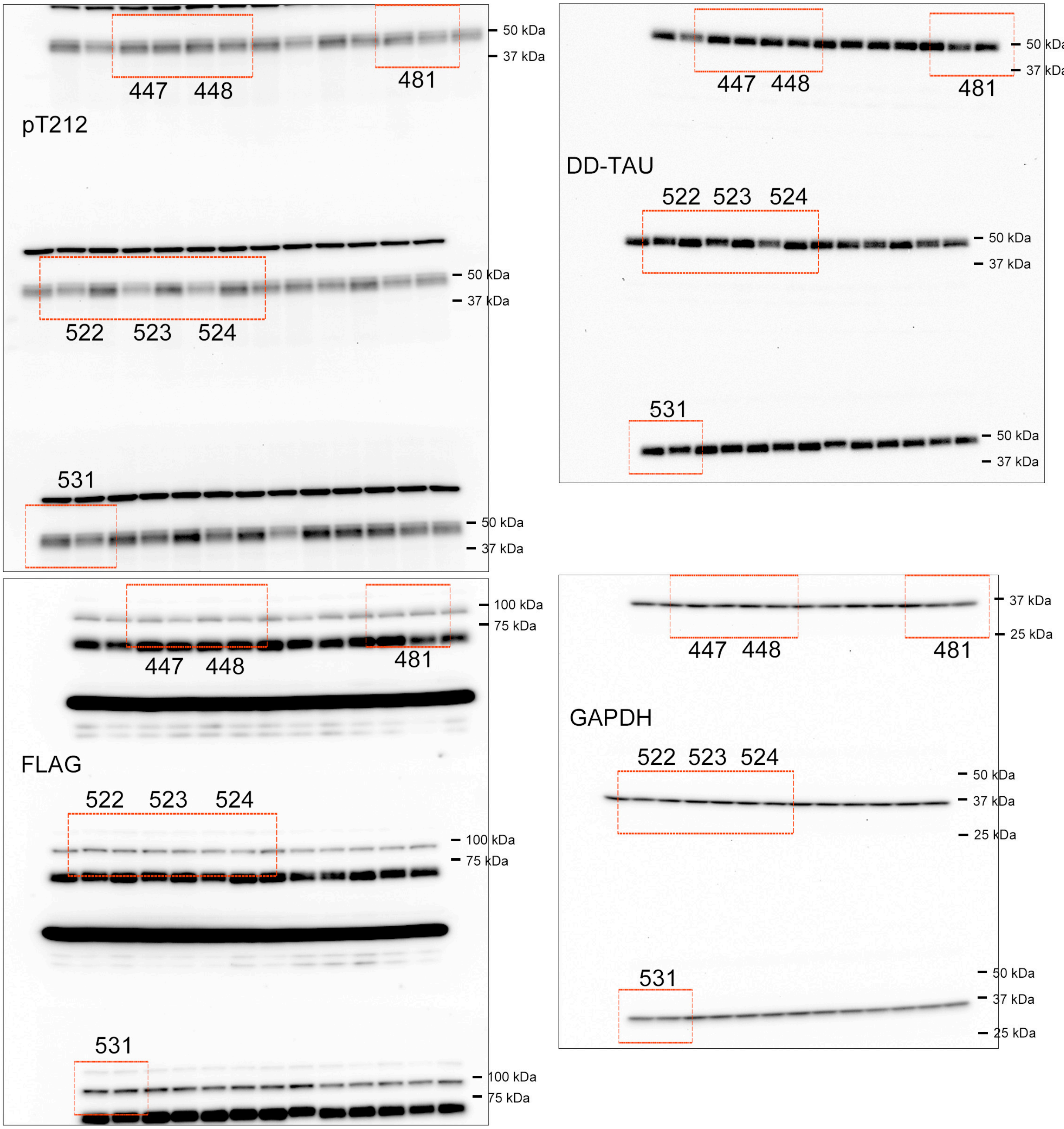

Supplementary Fig. 13b

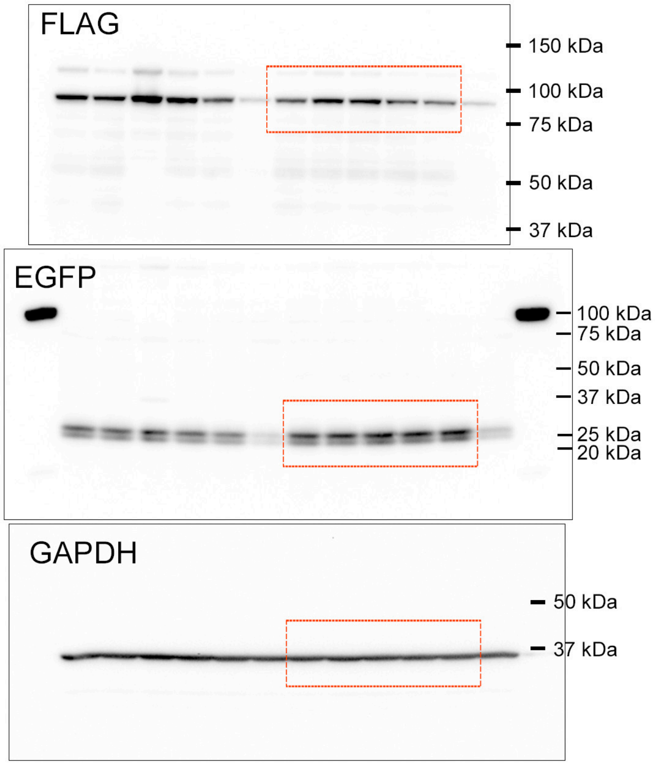

Supplementary Fig. 13c

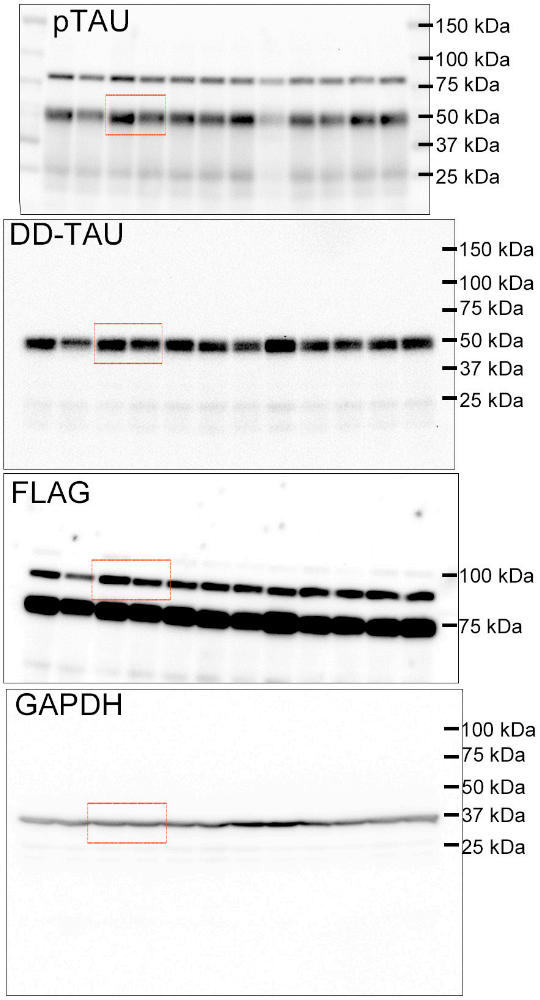

Supplementary Fig. 11b

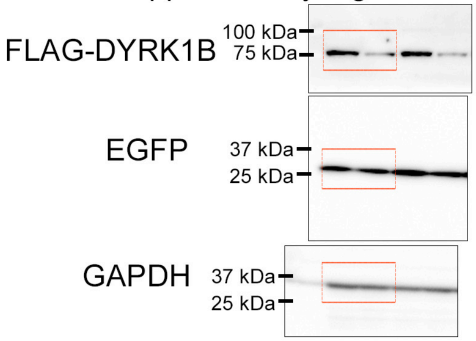

Supplementary Table 1

The per cent inhibition of kinase activity in the presence of 10 μM of FINDY relative to a solvent control (DMSO) is shown as the average of two replicates.

GSK3β, MARK4, PIM1, PIM3, and PLK3 were inhibited by over 75%, and none of these experienced an inhibition of over 85%.

Kinases	% Inhibition	Kinases	% Inhibition
ABL	13.0	FES	1.6
ACK	–2.2	FGFR1	37.0
ALK	–4.3	FGFR2	19.7
ARG	15.2	FGFR3	–0.5
AXL	6.7	FGFR4	6.9
BLK	25.5	FGR	5.3
BMX	10.5	FLT1	6.6
BRK	3.0	FLT3	16.0
BTK	57.9	FLT4	9.8
CSK	6.7	FMS	6.8
DDR1	–3.2	FRK	8.4
DDR2	13.5	FYN	58.4
EGFR	–5.1	HCK	35.3
EPHA1	40.5	HER2	–0.1
EPHA2	16.3	HER4	–13.4
EPHA3	20.8	IGF1R	2.4
EPHA4	16.3	INSR	–1.3
EPHA5	18.4	IRR	3.0
EPHA6	59.4	ITK	1.2
EPHA7	18.7	JAK1	5.7
EPHA8	48.8	JAK2	7.1
EPHB1	13.9	JAK3	8.2
EPHB2	10.1	KDR	1.6
EPHB3	22.2	KIT	2.9
EPHB4	20.5	LCK	7.2
FAK	7.3	LTK	4.6
FER	14.3	LYNa	4.0
LYNb	11.4	BRSK2	5.7
MER	3.4	CaMK1α	–4.1

MET	12.4	CaMK1δ	7.2
MUSK	4.1	CaMK2α	–6.9
PDGFRα	14.8	CaMK2β	–3.3
PDGFRβ	8.2	CaMK2γ	–4.6
PYK2	3.6	CaMK2δ	–4.0
RET	12.6	CaMK4	2.4
RON	9.2	CDC2/CycB1	43.4
ROS	0.3	CDC7/ASK	14.1
SRC	5.6	CDK2/CycA2	31.3
SRM	32.9	CDK2/CycE1	64.9
SYK	72.8	CDK3/CycE1	20.7
TEC	32.0	CDK4/CycD3	8.0
TIE2	55.7	CDK5/p25	35.6
TNK1	7.3	CDK6/CycD3	3.2
TRKA	–0.6	CDK7/CycH/MAT1	15.2
TRKB	4.9	CDK9/CycT1	3.1
TRKC	11.8	CGK2	14.6
TXK	23.7	CHK1	–118.9
TYK2	–5.4	CHK2	6.3
TYRO3	2.9	CK1α	6.1
YES	6.4	CK1γ1	19.9
ZAP70	12.9	CK1γ2	27.7
AKT1	36.6	CK1γ3	15.9
AKT2	23.5	CK1δ	29.1
AKT3	18.9	CK1ε	33.9
AMPKα1/β1/γ1	8.6	CK2α1/β	45.4
AMPKα2/β1/γ1	1.7	CK2α2/β	72.5
AurA	58.7	CLK1	10.4
AurA/TPX2	25.1	CLK2	2.0
AurB	35.7	CLK3	–12.4
AurC	40.1	COT_Cascade	55.4
BRAF_Cascade	33.9	CRIK	1.9
BRSK1	24.3	DAPK1	14.8
DCAMKL2	–3.8	MAP3K5_Cascade	32.1
DLK_Cascade	23.5	MAP4K2	3.3

EEF2K	–3.7	MAPKAPK2	51.9
Erk1	33.8	MAPKAPK3	29.2
Erk2	36.4	MAPKAPK5	5.2
Erk5	–4.0	MARK1	46.7
GSK3α	43.4	MARK2	60.8
GSK3β	80.1	MARK3	50.6
Haspin	30.3	MARK4	79.8
HGK	13.4	MELK	20.6
HIPK1	11.8	MGC42105	12.5
HIPK2	6.2	MINK	5.0
HIPK3	7.1	MLK1_Cascade	48.7
HIPK4	16.6	MLK2_Cascade	41.0
IKKα	4.9	MLK3_Cascade	40.5
IKKβ	–3.5	MNK1	6.2
IKKε	–2.0	MNK2	8.3
IRAK1	–1.1	MOS_Cascade	60.1
IRAK4	–0.8	MRCKα	2.7
JNK1	1.5	MRCKβ	0.1
JNK2	0.4	MSK1	3.8
JNK3	4.5	MSK2	20.8
LATS2	1.2	MSSK1	6.6
LOK	–28.2	MST1	–5.2
MAP2K1_Cascade	44.2	MST2	–5.7
MAP2K2_Cascade	38.8	MST3	6.4
MAP2K3_Cascade	26.0	MST4	16.7
MAP2K4_Cascade	6.4	NDR1	3.8
MAP2K5_Cascade	–0.1	NDR2	8.1
MAP2K6_Cascade	23.5	NEK1	37.3
MAP2K7_Cascade	14.4	NEK2	–5.1
MAP3K1_Cascade	44.4	NEK4	6.8
MAP3K2_Cascade	55.8	NEK6	23.6
MAP3K3_Cascade	3.3	NEK7	–12.3
MAP3K4_Cascade	39.8	NEK9	–3.3
NuaK1	–26.4	PKCζ	–6.1
NuaK2	–9.3	PKCη	–12.7

p38α	10.6	PKCθ	6.5
p38β	11.2	PKCι	–4.3
p38γ	5.0	PKD1	11.8
p38δ	11.9	PKD2	29.9
p70S6K	7.4	PKD3	–13.4
p70S6Kβ	19.9	PKN1	–0.9
PAK1	–0.2	PKR	5.8
PAK2	–2.5	PLK1	23.7
PAK3	–14.7	PLK2	11.1
PAK4	–20.1	PLK3	76.3
PAK5	–7.0	PRKX	3.0
PAK6	–145.1	QIK	–7.7
PASK	11.9	RAF1_Cascade	50.9
PBK	14.6	ROCK1	6.8
PDHK2	14.1	ROCK2	–2.0
PDHK4	14.8	RSK1	39.8
PDK1	–2.6	RSK2	39.9
PEK	13.0	RSK3	28.0
PGK	2.6	RSK4	29.6
PHKG1	8.9	SGK	27.8
PHKG2	1.9	SGK2	30.9
PIM1	76.8	SGK3	21.7
PIM2	67.4	SIK	15.0
PIM3	83.1	skMLCK	2.0
PKACα	64.7	SLK	–35.0
PKACβ	65.3	SRPK1	7.5
PKACγ	17.8	SRPK2	–5.0
PKCα	0.6	TAK1-TAB1_Cascade	52.7
PKCβ1	3.5	TAOK2	–1.2
PKCβ2	–2.3	TBK1	4.0
PKCγ	4.1	TNIK	10.7
PKCδ	–1.3	TSSK1	3.3
PKCε	–6.6	TSSK2	39.9
TSSK3	20.3	PIK3CA/PIK3R1	12.8
WNK1	12.6	SPHK1	51.2

WNK2	12.3	SPHK2	4.9
WNK3	18.9		

DYRK1A	49.0	DYRK2	47.2
DYRK1B	35.3	DYRK3	44.2

Supplementary Table 2. SPHINKS assay data

Category	Parameter	Description
Assay	Type of assay	Cell-based
	Target	DYRK1A transitional intermediate
	Measurement	Detection of TAU phosphorylation mediated by mature DYRK1A
	Key reagents	Rabbit polyclonal antibody against phosphorylated TAU (phospho-Thr212) (Life Technologies)
		Mouse monoclonal antibody against TAU (TAU-5) (CALBIOCHEM)
		ProteoTuner Shield System (Clontech Laboratories)
		pcDNA5/FRT/TO vector and Flp-In/T-REx293 cells (Life Technologies)
	Assay protocol	Described in the sections "Methods" and "Results"
Library	Library size	Approximately 200
	Library composition	Synthetic chemical compounds, including derivatives of RD0392, CaNDY, and INDY
	Source	Laboratory of Chemical Bioscience, Institute of Biomaterials and Bioengineering, Tokyo Medical and Dental University
Screen	Format	6-well plates (FALCON) coated with polyethylenimine
Screen	Concentration(s) tested	10 μM (0.1% DMSO) in DMEM with 1%FBS
	Control	INDY was used as a control for screening
	Reagent/ compound dispensing system	Manual
	Detection instrument and software	Western blot using primary anti-phospho-TAU and total TAU antibodies, followed by HRP-conjugated secondary antibodies
		Chemiluminescence of HRP was detected by the ChemiDox XRS Imaging System (BIO-RAD)
	Normalization	The amount of phosphorylated TAU protein was normalized by the amount of total TAU protein on the same membrane
	Additional comments	The throughput of this screening is low

Supplementary Methods

Chemical Synthesis

General notes: Melting points (mp) were measured on the OptiMelt MPA100 automated melting point apparatus (Stanford Research Systems), and are uncorrected. IR spectra were measured by the diffuse reflectance method on the Shimadzu IRPrestige-21 spectrometer with an attached DRS-8000A, with the absorption band given in cm^{-1}. ^{1}H and ^{13}C NMR spectra were obtained with the JEOL 400SS spectrometer at 400 and 100 MHz, respectively. DMSO-d_6 (CIL, Cat. No. DLM-10) was used as solvent for obtaining the NMR spectra. Chemical shifts (δ) are given in parts per million (ppm) downfield from the solvent peak (δ 2.49 for ^{1}H NMR and δ 39.5 for ^{13}C NMR in DMSO-d_6) as an internal reference, with the coupling constants (J) in hertz (Hz). The abbreviations s, d, t, q, m, and br signify singlet, doublet, triplet, quartet, multiplet, and broad, respectively. High-resolution mass spectra (HRMS) were obtained with a Bruker micrOTOF mass spectrometer under negative or positive electrospray ionization (ESI^- or ESI^+) conditions at Tokyo Medical and Dental University or a JEOL JMS-T100LC AccuTOF mass spectrometer under electronspray ionization (ESI^-) conditions or a JEOL JMS-700V under positive or negative electrospray ionization (ESI^-) conditions at Molecular Characterization, Collaboration Promotion Unit, RIKEN.

The rhodanine and thiohydantoin derivatives were synthesized by Knoevenagel condensation with the corresponding aldehydes, which were either purchased or easily prepared from commercial 3-bromoanisaldehyde or 4-hydroxy-3-iodobenzaldehyde by the Suzuki–Miyaura or Sonogashira cross-coupling reaction under normal conditions.

(*Z*)-5-{4-Methoxy-3-[(trimethylsilyl)ethynyl]benzylidene}-2-thioxothiazolidin-4-one (FINDY)

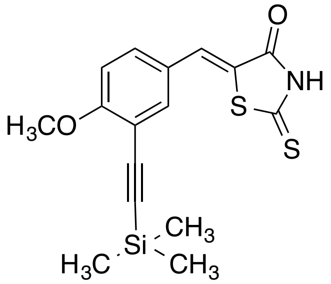

Under an argon atmosphere, acetic acid (0.500 mL, 8.64 mmol) was added to a solution of 4-methoxy-3-[2-(trimethylsilyl)ethynyl]benzaldehyde (2.01 g, 8.63 mmol), rhodanine (1.15 g, 8.63 mmol), and ammonium acetate (77.0 mg, 1.00 mmol) in acetonitrile (50 mL) at room temperature, and the mixture was heated with stirring for 3 h at 80 ºC. After cooling to room temperature, the resulting yellow solid was collected by filtration, washed with water (ca. 10 mL ×4) and diethyl ether (ca. 5 mL ×2) on the funnel, then dried under reduced pressure to give (*Z*)-5-{4-methoxy-3-[(trimethylsilyl)ethynyl]benzylidene}-2-thioxothiazolidin-4-one (FINDY) (2.64 g, 7.60 mmol, 88.0%) as a yellow solid. Details: mp 215–216 ºC; IR (KBr, cm^{-1}) 849, 1275, 1433, 1499, 1586, 1692, 2839, 2895, 2943, 2970, 3038, 3057, 3152; ^{1}H NMR (DMSO-d_6, 400 MHz) δ 0.24 (s, 9H), 3.89 (s, 3H), 7.24 (d, J = 8.8 Hz, 1H), 7.58–7.64 (m, 3H), 13.80 (br s, 1H); ^{13}C NMR (DMSO-d_6, 100 MHz) δ –0.1 (3C), 56.2, 99.2, 100.4, 112.3, 112.5, 123.3, 125.4, 130.7, 132.6, 136.0, 161.5, 169.3, 195.2; HRMS (ESI$^-$) m/z 346.0404 (346.0397 calculated for $C_{16}H_{16}NO_2S_2Si^-$, [M–H]$^-$).

(Z)-5-(3-Ethoxy-4-hydroxybenzylidene)-2-thioxothiazolidin-4-one (RD0392)

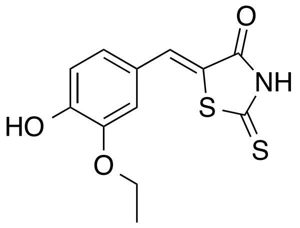

Under an argon atmosphere, acetic acid (60 μL, 1.1 mmol) was added to a solution of 3-ethoxy-4-hydroxybenzaldehyde (166 mg, 0.999 mmol), rhodanine (133 mg, 1.00 mmol), and ammonium acetate (38.5 mg, 0.499 mmol) in acetonitrile (2 mL) at room temperature, and the mixture was heated with stirring for 3 h at 80 ºC. After cooling to room temperature, the resulting yellow solid was collected by filtration, washed with water (ca. 3 mL ×4) and diethyl ether (ca. 3 mL ×2) on the funnel, then dried under reduced pressure to give (*Z*)-5-(3-ethoxy-4-hydroxybenzylidene)-2-thioxothiazolidin-4-one (RD0392) (231 mg, 0.821 mmol, 82.2%) as a yellow solid. Details: mp 217–218 ºC; IR (KBr, cm^{-1}) 818, 1036, 1184, 1285, 1445, 1514, 1694, 2984, 3017, 3046, 3848; ^{1}H NMR (DMSO-d_6, 400 MHz) δ 1.36 (t, J = 6.8 Hz, 3H), 4.07 (q, J = 6.8 Hz, 2H), 6.94 (d, J = 8.4 Hz, 1H), 7.07 (d, J = 8.4 Hz, 1H), 7.13 (s, 1H), 7.56 (s, 1H), 10.01 (s, 1H), 13.70 (br s, 1H); ^{13}C NMR (DMSO-d_6, 100 MHz) δ 14.6, 63.9, 115.3, 116.4, 121.0, 124.4, 125.1, 132.9, 147.3, 150.2, 169.4, 195.5; HRMS (ESI$^-$) m/z 280.0105 (280.0108 calculated for $C_{12}H_{10}NO_3S_2^-$, [M–H]$^-$).

(*Z*)-5-{4-Methoxy-3-[(trimethylsilyl)ethynyl]benzylidene}-2-thioxoimidazolidin-4-one (RD0447)

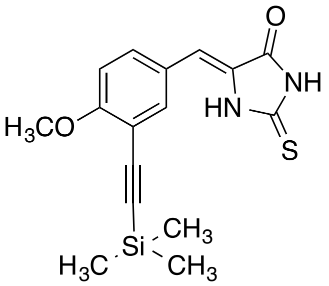

Under an argon atmosphere, acetic acid (60 μL, 1.0 mmol) was added to a solution of 4-methoxy-3-[2-(trimethylsilyl)ethynyl]benzaldehyde (232 mg, 1.00 mmol), thiohydantoin (116 mg, 1.00 mmol), and ammonium acetate (38.5 mg, 0.500 mmol) in acetonitrile (5 mL) at room temperature, and the mixture was heated with stirring for 3 h at 90 ºC. After cooling to room temperature, the resulting yellow solid was collected by filtration, washed with water (ca. 5 mL ×3) and diethyl ether (ca. 5 mL ×2) on the funnel, then dried under reduced pressure to give (*Z*)-5-{4-methoxy-3-[(trimethylsilyl)ethynyl]benzylidene}-2-thioxoimidazolidin-4-one (RD0447) (56.4 mg, 0.171 mmol, 17.1%) as a brown solid. Details: mp 232–233 ºC; ^{1}H NMR (DMSO-d_6, 400 MHz) δ 0.23 (s, 9H), 3.86 (s, 3H), 6.42 (s, 1H), 7.08 (d, J = 8.8 Hz, 1H), 7.74 (dd, J = 2.0, 8.8 Hz, 1H), 7.79 (d, J = 2.0 Hz, 1H), 12.05–12.41 (br, 2H); ^{13}C NMR (DMSO-d_6, 100 MHz) δ 0.0 (3C), 56.1, 98.6, 101.1, 111.0, 111.6, 111.8, 124.8, 126.6, 133.1, 134.8, 160.7, 165.7, 178.9; HRMS (ESI$^-$) m/z 329.0781 (329.0780 calculated for $C_{16}H_{17}N_2O_2SSi^-$, [M–H]$^-$).

(*Z*)-5-(3-Ethynyl-4-methoxybenzylidene)-2-thioxothiazolidin-4-one (RD0448)

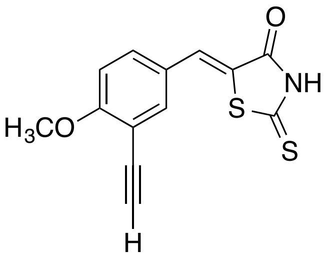

Under an argon atmosphere, acetic acid (24 μL, 0.42 mmol) was added to a solution of 3-ethynyl-4-methoxybenzaldehyde (65.0 mg, 0.406 mmol), rhodanine (54.0 mg, 0.405 mmol), and ammonium acetate (16.0 mg, 0.208 mmol) in acetonitrile (0.8 mL) at room temperature, and the mixture was heated with stirring for 6 h at 90 ºC. After cooling to room temperature, the resulting yellow solid was collected by filtration, washed with water (ca. 5 mL ×3) and diethyl ether (ca. 5 mL ×2) on the funnel, then dried under reduced pressure to give (*Z*)-5-(3-ethynyl-4-methoxybenzylidene)-2-thioxothiazolidin-4-one (RD0448) (84.3 mg, 0.306 mmol, 75.6%) as a yellow solid. Details: mp 190–193 ºC (dec.); ^{1}H NMR (DMSO-d_6, 400 MHz) δ 3.89 (s, 3H), 4.38 (s, 1H), 7.24 (d, J = 8.6 Hz, 1H), 7.57–7.67 (m, 3H), 13.82 (br s, 1H); ^{13}C NMR (DMSO-d_6, 100 MHz) δ 56.2, 78.9, 85.7, 111.9, 112.4, 123.6, 125.5, 130.6, 132.7, 136.0, 161.7, 169.5, 195.4; HRMS (ESI$^-$) m/z 273.9995 (273.9996 calculated for $C_{13}H_8NO_2S_2^-$, [M–H]$^-$).

(*Z*)-5-(4-Methoxy-3-methylbenzylidene)-2-thioxothiazolidin-4-one (RD0481)

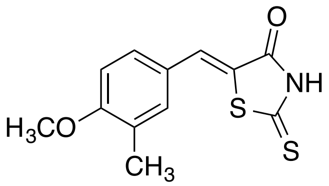

Under an argon atmosphere, acetic acid (60 μL, 1.0 mmol) was added to a solution of 4-methoxy-3-methylbenzaldehyde (150 mg, 1.00 mmol), rhodanine (133 mg, 1.00 mmol), and ammonium acetate (38.5 mg, 0.500 mmol) in acetonitrile (2 mL) at room temperature, and the mixture was heated with stirring for 2 h at 90 ºC. After cooling to room temperature, the resulting yellow solid was collected by filtration, washed with water (ca. 5 mL ×3) and diethyl ether (ca. 5 mL ×2) on the funnel, then dried under reduced pressure to give (*Z*)-5-(4-methoxy-3-methylbenzylidene)-2-thioxothiazolidin-4-one (RD0481) (197 mg, 0.742 mmol, 74.2%) as a yellow solid. Details: mp 218–219 ºC; ^{1}H NMR (DMSO-d_6, 400 MHz) δ 2.19 (s, 3H), 3.86 (s, 3H), 7.12 (d, J = 8.6 Hz, 1H), 7.38 (s, 1H), 7.47 (d, J = 8.6 Hz, 1H), 7.56 (s, 1H), 13.74 (br s, 1H); ^{13}C NMR (DMSO-d_6, 100 MHz) δ 16.0, 55.7, 111.2, 121.9, 125.0, 127.0, 130.9, 132.1, 132.6, 159.6, 169.5, 195.6; HRMS (ESI$^-$) m/z 264.0154 (264.0153 calculated for $C_{12}H_{10}NO_2S_2^-$, [M–H]$^-$).

(*Z*)-5-[4-Methoxy-3-(4-methylphenyl)benzylidene]-2-thioxothiazolidin-4-one (RD0522)

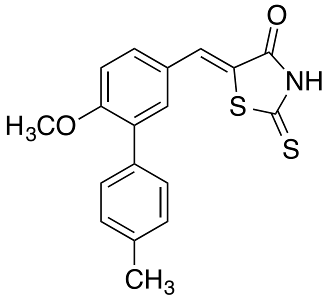

Under an argon atmosphere, acetic acid (140 μL, 2.45 mmol) was added to a solution of 4-methoxy-3-(4-methylphenyl)benzaldehyde (566 mg, 2.50 mmol), rhodanine (333 mg, 2.50 mmol), and ammonium acetate (96.4 mg, 1.25 mmol) in acetonitrile (5 mL) at room temperature, and the mixture was heated with stirring for 6 h at 90 ºC. After cooling to room temperature, the resulting yellow solid was collected by filtration, washed with water (ca. 10 mL ×3) and diethyl ether (ca. 10 mL ×2) on the funnel, then dried under reduced pressure to give (*Z*)-5-[4-methoxy-3-(4-methylphenyl)benzylidene]-2-thioxothiazolidin-4-one (RD0522) (313 mg, 0.916 mmol, 36.7%) as a yellow solid. Details: mp 216–217 ºC; ^{1}H NMR (DMSO-d_6, 400 MHz) δ 2.34 (s, 3H), 3.84 (s, 3H), 7.25 (d, J = 8.0 Hz, 2H), 7.28 (d, J = 8.8 Hz, 1H), 7.39 (d, J = 8.0 Hz, 2H), 7.50 (d, J = 2.5 Hz, 1H), 7.60 (dd, J = 2.5, 8.8 Hz, 1H), 7.67 (s, 1H), 13.78 (br s, 1H); ^{13}C NMR (DMSO-d_6, 100 MHz) δ 20.8, 56.0, 112.7, 122.5, 125.7, 128.8 (2C), 129.1 (2C), 130.7, 131.6, 131.9, 132.8, 134.0, 136.7, 158.3, 169.4, 195.4; HRMS (ESI$^-$) m/z 340.0467 (340.0466 calculated for $C_{18}H_{14}NO_2S_2^-$, [M–H]$^-$).

(*Z*)-5-[4-Methoxy-3-(3-methylphenyl)benzylidene]-2-thioxothiazolidin-4-one (RD0523)

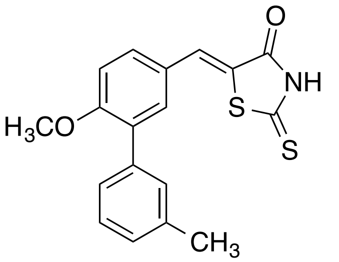

Under an argon atmosphere, acetic acid (57 μL, 1.0 mmol) was added to a solution of 4-methoxy-3-(3-methylphenyl)benzaldehyde (226 mg, 1.00 mmol), rhodanine (133 mg, 1.00 mmol), and ammonium acetate (38.5 mg, 0.499 mmol) in acetonitrile (2 mL) at room temperature, and the mixture was heated with stirring for 5 h at 90 ºC. After cooling to room temperature, the resulting orange solid was collected by filtration, washed with water (ca. 5 mL ×3) and diethyl ether (ca. 5 mL ×2) on the funnel, then dried under reduced pressure to give (*Z*)-5-[4-methoxy-3-(3-methylphenyl)benzylidene]-2-thioxothiazolidin-4-one (RD0523) (311 mg, 0.911 mmol, 91.1%) as an orange solid. Details: mp 204–205 ºC; ^{1}H NMR (DMSO-d_6, 400 MHz) δ 2.34 (s, 3H), 3.83 (s, 3H), 7.17 (d, J = 7.3 Hz, 1H), 7.26–7.34 (m, 4H), 7.49 (d, J = 2.3 Hz, 1H), 7.59 (dd, J = 2.3, 8.8 Hz, 1H), 7.66 (s, 1H), 13.77 (br s, 1H); ^{13}C NMR (DMSO-d_6, 100 MHz) δ 21.1, 56.0, 112.6, 122.5, 125.6, 126.4, 128.0 (2C), 129.8, 130.9, 131.6, 131.8, 133.1, 136.9, 137.2, 158.2, 169.3, 195.3; HRMS (ESI$^-$) m/z 340.0470 (340.0466 calculated for $C_{18}H_{14}NO_2S_2^-$, [M–H]$^-$).

(*Z*)-5-[4-Methoxy-3-(phenylethynyl)benzylidene]-2-thioxothiazolidin-4-one (RD0524)

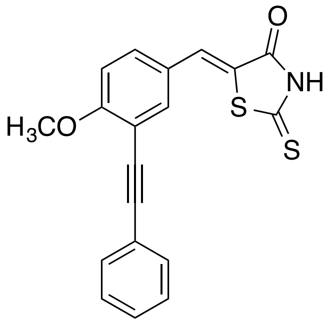

Under an argon atmosphere, acetic acid (140 μL, 2.45 mmol) was added to a solution of 4-methoxy-3-(phenylethynyl)benzaldehyde (591 mg, 2.50 mmol), rhodanine (333 mg, 2.50 mmol), and ammonium acetate (96.4 mg, 1.25 mmol) in acetonitrile (5 mL) at room temperature, and the mixture was heated with stirring for 10 h at 90 ºC. After cooling to room temperature, the resulting orange solid was collected by filtration, washed with water (ca. 10 mL ×3) and diethyl ether (ca. 10 mL ×2) on the funnel, then dried under reduced pressure to give (*Z*)-5-[4-methoxy-3-(phenylethynyl)benzylidene]-2-thioxothiazolidin-4-one (RD0524) (619 mg, 1.76 mmol, 70.5%) as an orange solid. Details: mp 224–225 ºC; ^{1}H NMR (DMSO-d_6, 400 MHz) δ 3.94 (s, 3H), 7.28 (d, J = 8.8 Hz, 1H), 7.41–7.44 (m, 3H), 7.53–7.56 (m, 2H), 7.62 (s, 1H), 7.63 (dd, J = 2.0, 8.8 Hz, 1H), 7.72 (d, J = 2.0 Hz, 1H), 13.80 (br s, 1H); ^{13}C NMR (DMSO-d_6, 100 MHz) δ 56.3, 84.9, 93.9, 112.5 (2C), 122.2, 123.4, 125.6, 128.8, 128.9 (2C), 130.8, 131.4 (2C), 132.7, 135.4, 161.2, 169.4, 195.3; HRMS (ESI$^-$) m/z 350.0306 (350.0309 calculated for $C_{19}H_{12}NO_2S_2^-$, [M–H]$^-$).

(*Z*)-5-[(3-Cyclohexylethynyl)-4-methoxybenzylidene]-2-thioxothiazolidin-4-one (RD0531)

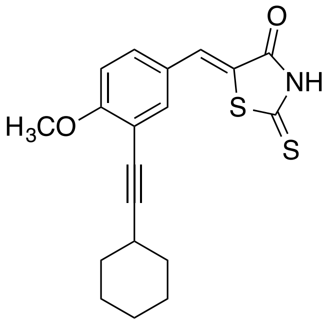

Under an argon atmosphere, acetic acid (57 μL, 1.0 mmol) was added to a solution of 3-(cyclohexylethynyl)-4-methoxybenzaldehyde (242 mg, 1.00 mmol), rhodanine (133 mg, 1.00 mmol), and ammonium acetate (38.5 mg, 0.499 mmol) in acetonitrile (2 mL) at room temperature, and the mixture was heated with stirring for 2 h at 80 ºC. After cooling to room temperature, the resulting yellow solid was collected by filtration, washed with water (ca. 5 mL ×3) and diethyl ether (ca. 5 mL ×2) on the funnel, then dried under reduced pressure to give (*Z*)-5-[(3-cyclohexylethynyl)-4-methoxybenzylidene]-2-thioxothiazolidin-4-one (RD0531) (207 mg, 0.579 mmol, 57.9%) as a yellow solid. Details: mp 185–186 ºC; ^{1}H NMR (DMSO-d_6, 400 MHz) δ 1.34–1.36 (m, 3H), 1.46–1.50 (m, 3H), 1.68–1.71 (m, 2H), 1.79–1.81 (m, 2H), 2.65–2.69 (m, 1H), 3.87 (s, 3H), 7.20 (d, J = 9.5 Hz, 1H), 7.53–7.59 (m, 3H), 13.79 (br s, 1H); ^{13}C NMR (DMSO-d_6, 100 MHz) δ 24.2 (2C), 25.4, 29.0, 32.1 (2C), 56.2, 75.9, 99.4, 112.2, 113.5, 123.1, 125.4, 131.1, 131.8, 135.4, 161.2, 169.4, 195.3; HRMS (ESI$^-$) m/z 356.0782 (356.0779 calculated for $C_{19}H_{18}NO_2S_2^-$, [M–H]$^-$).

(*Z*)-5-{4-Hydroxy-3-[(trimethylsilyl)ethynyl]benzylidene}-2-thioxothiazolidin-4-one (RD0561)

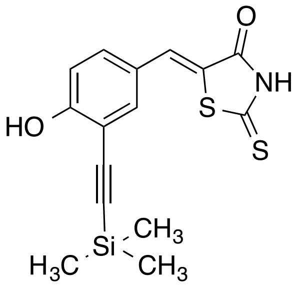

Under an argon atmosphere, acetic acid (12 μL, 0.21 mmol) was added to a solution of 4-hydroxy-3-[(trimethylsilyl)ethynyl]benzaldehyde (44.0 mg, 0.202 mmol), rhodanine (27.0 mg, 0.203 mmol), and ammonium acetate (8.0 mg, 0.10 mmol) in acetonitrile (0.5 mL) at room temperature, and the mixture was heated with stirring for 2 h at 80 ºC. After cooling to room temperature, the resulting yellow solid was collected by filtration, washed with water (ca. 5 mL ×3) and diethyl ether (ca. 5 mL ×2) on the funnel, then dried under reduced pressure to give (*Z*)-5-{4-Hydroxy-3-[(trimethylsilyl)ethynyl]benzylidene}-2-thioxothiazolidin-4-one (RD0561) (27.5 mg, 82.5 μmol, 40.8%) as a yellow solid. Details: mp 215–217 ºC (dec.); ^{1}H NMR (DMSO-d_6, 400 MHz) δ 0.22 (s, 9H), 7.04 (d, *J* = 8.8 Hz, 1H), 7.44 (d, *J* = 2.0, 8.8 Hz, 1H), 7.54 (s, 1H), 7.57 (d, *J* = 2.0 Hz, 1H), 11.00 (s, 1H), 13.75 (br s, 1H); ^{13}C NMR (DMSO-d_6, 100 MHz) δ 0.0 (3C), 98.4, 101.0, 111.0, 116.7, 122.2, 124.2, 131.3, 132.5, 136.7, 161.1, 169.4, 195.3; HRMS (ESI$^-$) m/z 332.0231 (332.0235 calculated for $C_{15}H_{14}NO_2S_2Si^-$, [M–H]$^-$).

^{1}H NMR (400 MHz) and ^{13}C NMR (100 MHz) spectra of **FINDY** (DMSO-d_6)

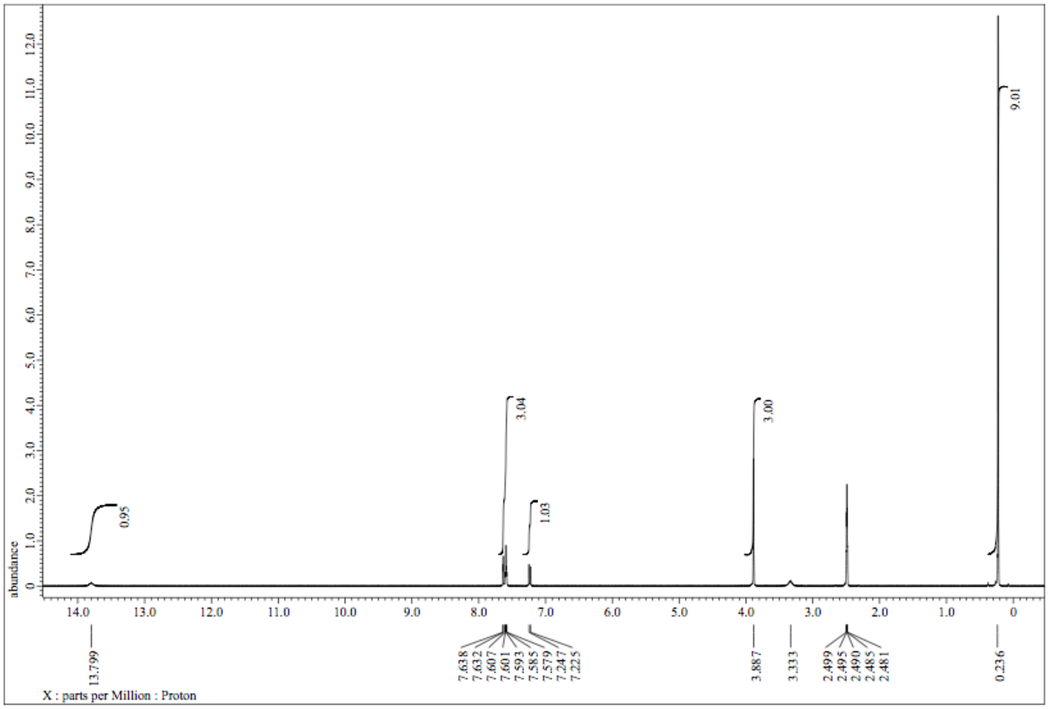

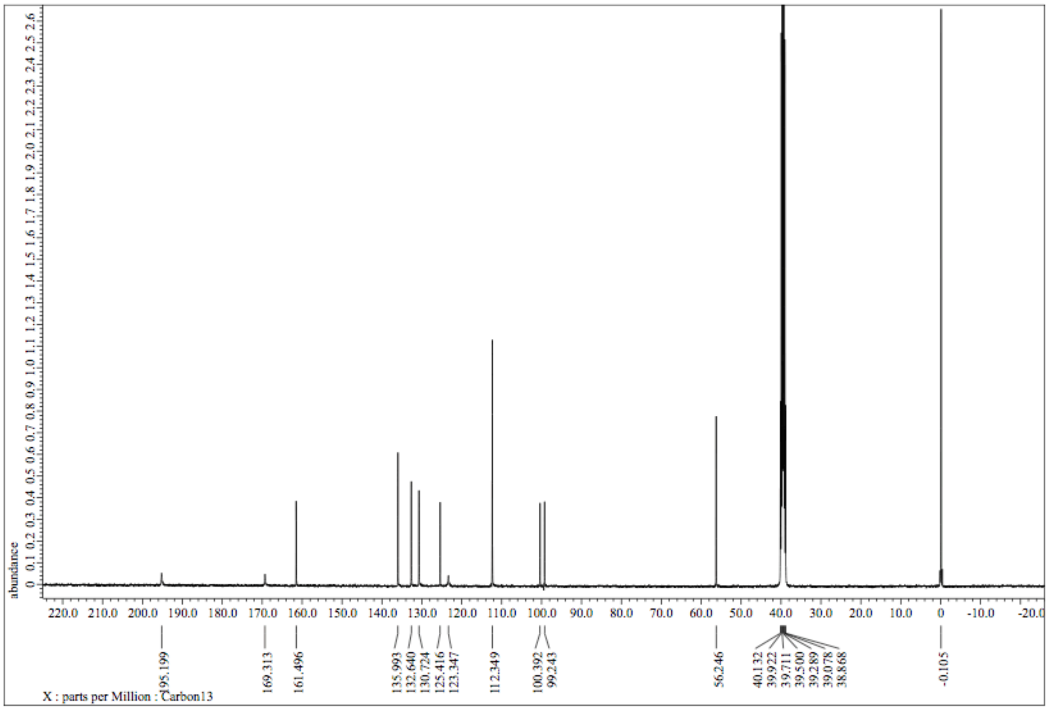

^{1}H NMR (400 MHz) and ^{13}C NMR (100 MHz) spectra of **RD0392** (DMSO-d_6)

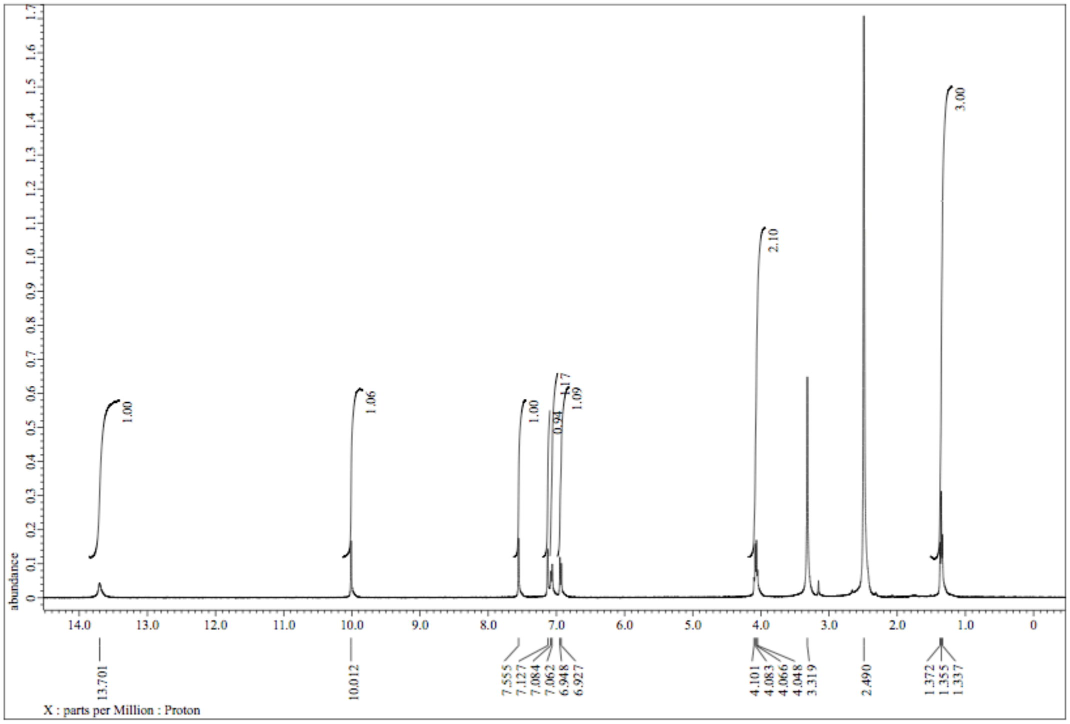

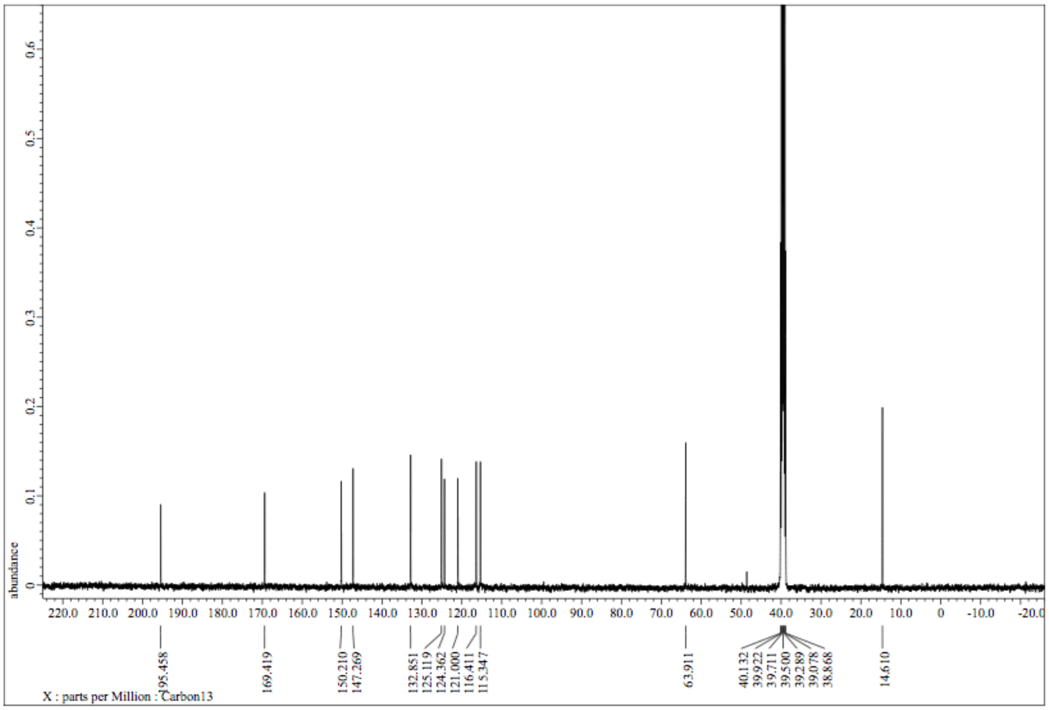

^{1}H NMR (400 MHz) and ^{13}C NMR (100 MHz) spectra of **RD0447** (DMSO-d_6)

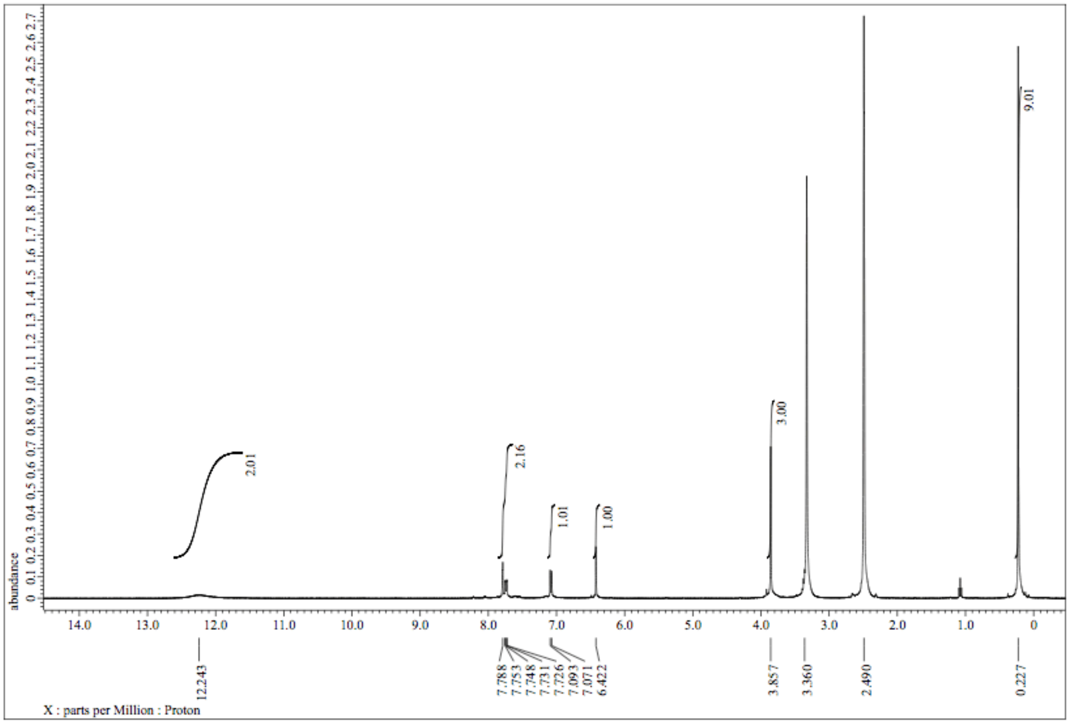

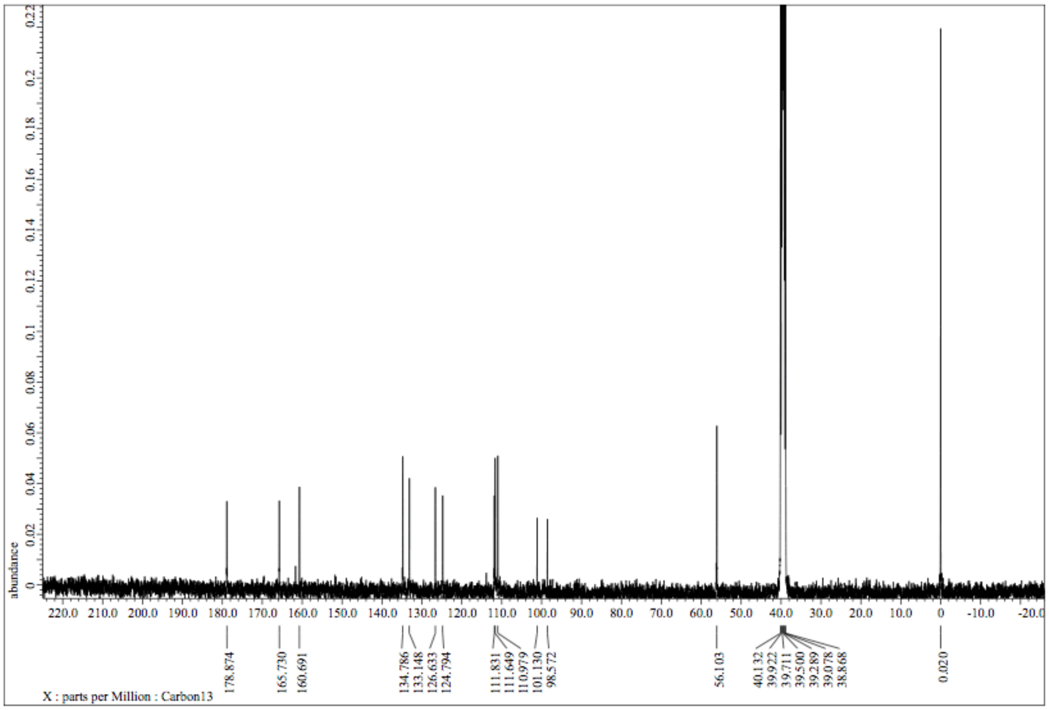

^{1}H NMR (400 MHz) and ^{13}C NMR (100 MHz) spectra of **RD0448** (DMSO-d_6)

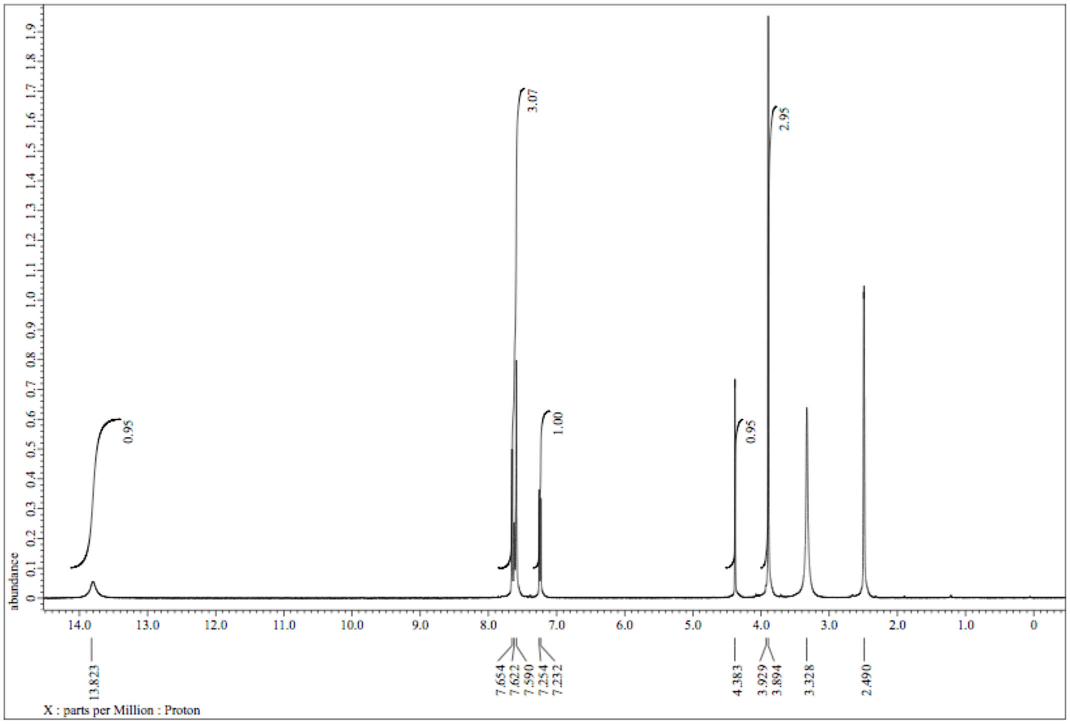

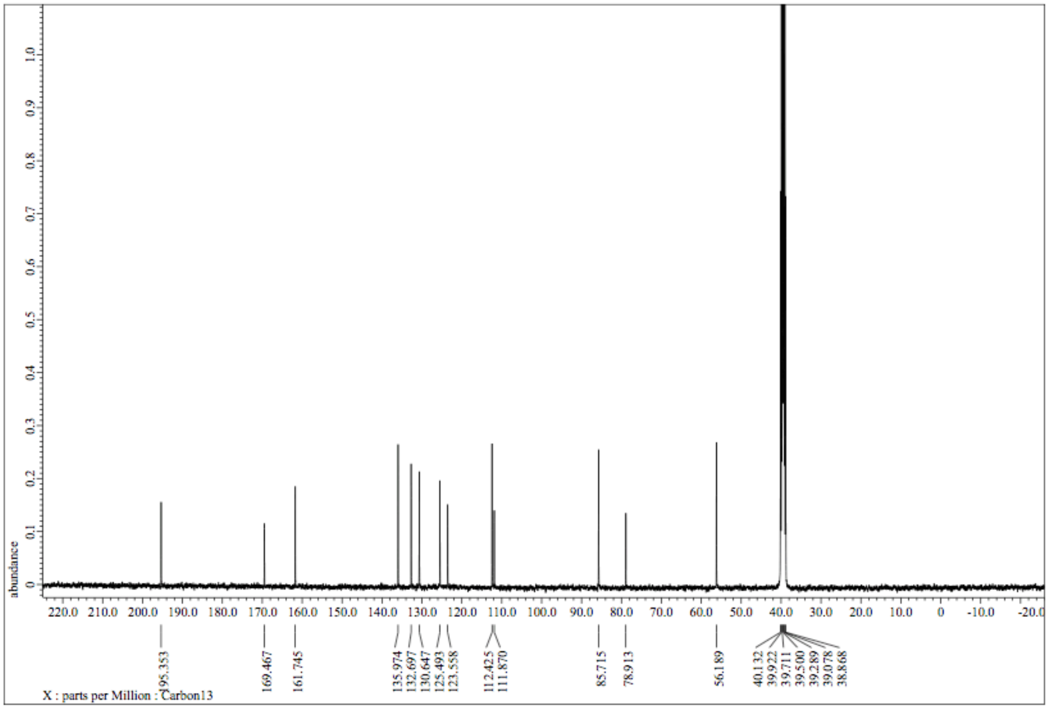

^{1}H NMR (400 MHz) and ^{13}C NMR (100 MHz) spectra of **RD0481** (DMSO-d_6)

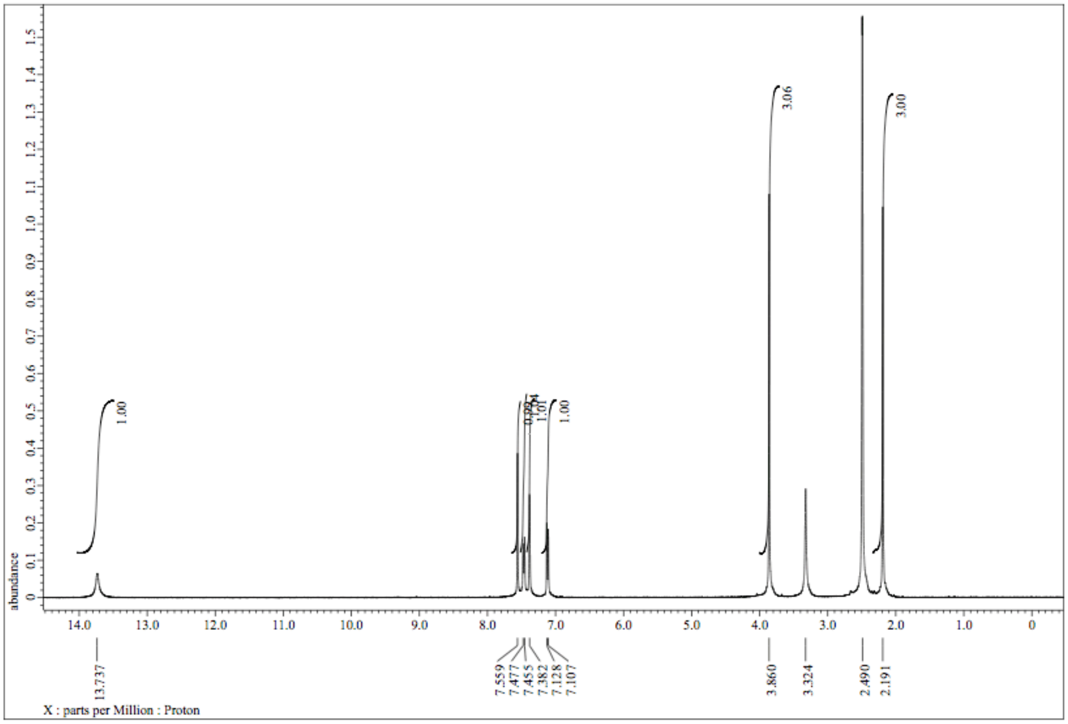

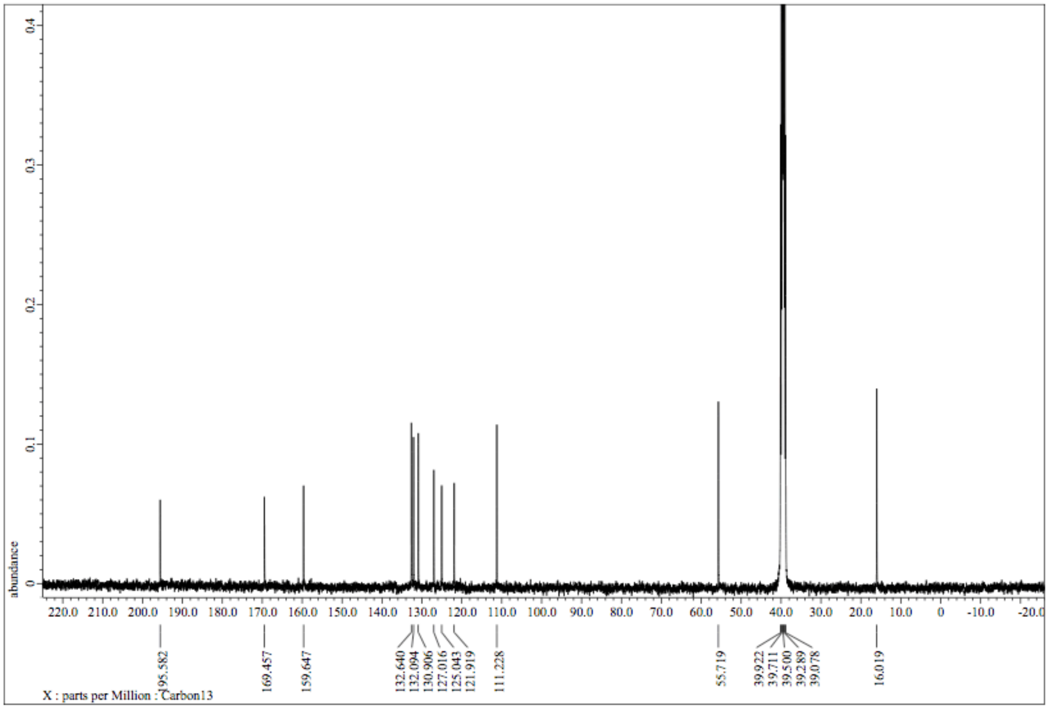

^{1}H NMR (400 MHz) and ^{13}C NMR (100 MHz) spectra of **RD0522** (DMSO-d_6)

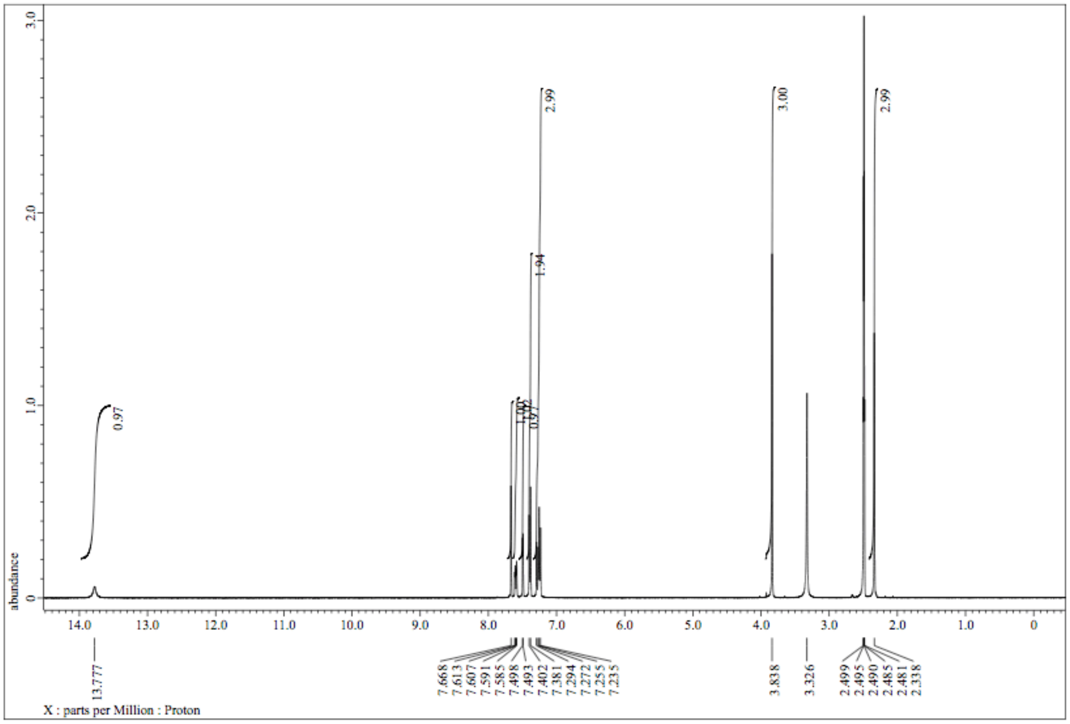

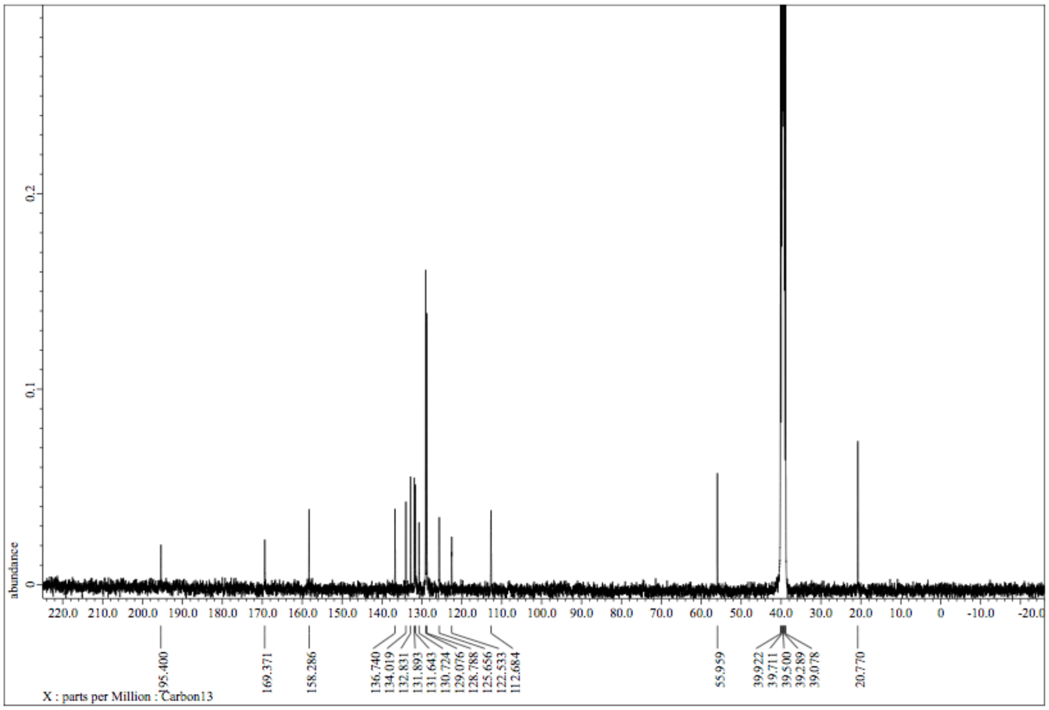

^{1}H NMR (400 MHz) and ^{13}C NMR (100 MHz) spectra of **RD0523** (DMSO-d_6)

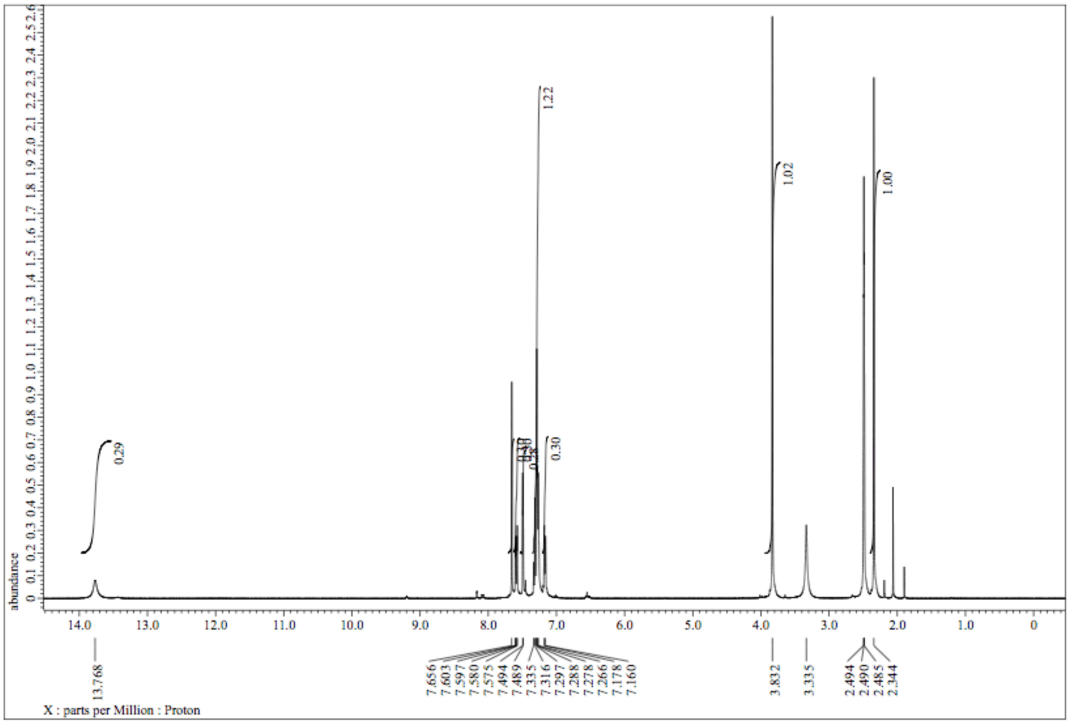

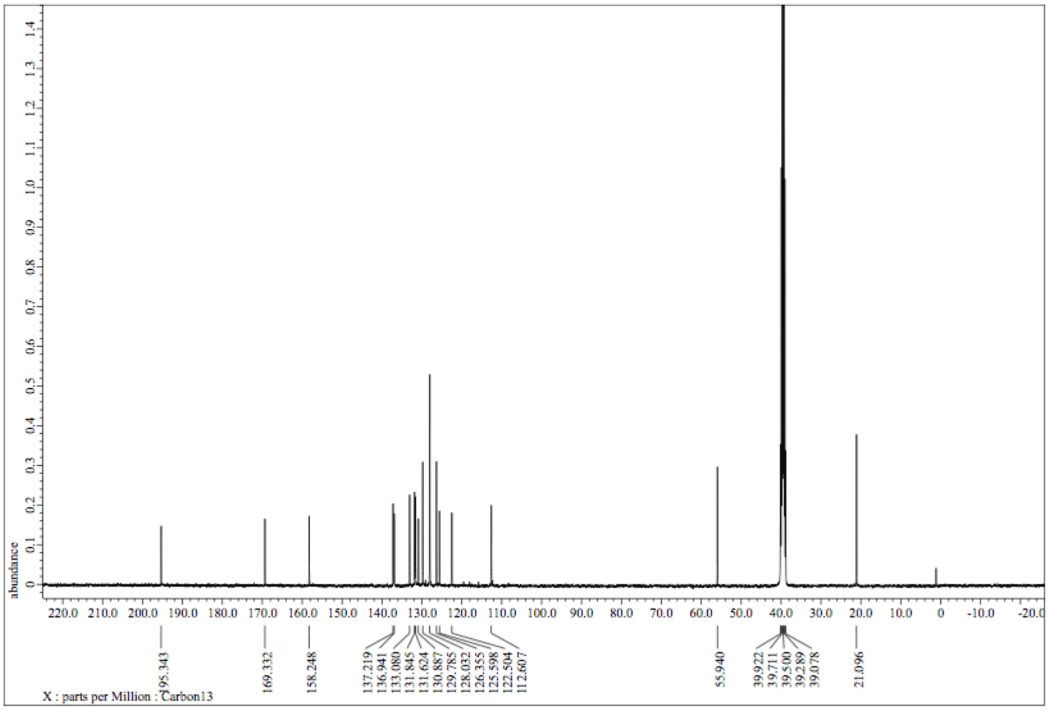

^{1}H NMR (400 MHz) and ^{13}C NMR (100 MHz) spectra of **RD0524** (DMSO-d_6)

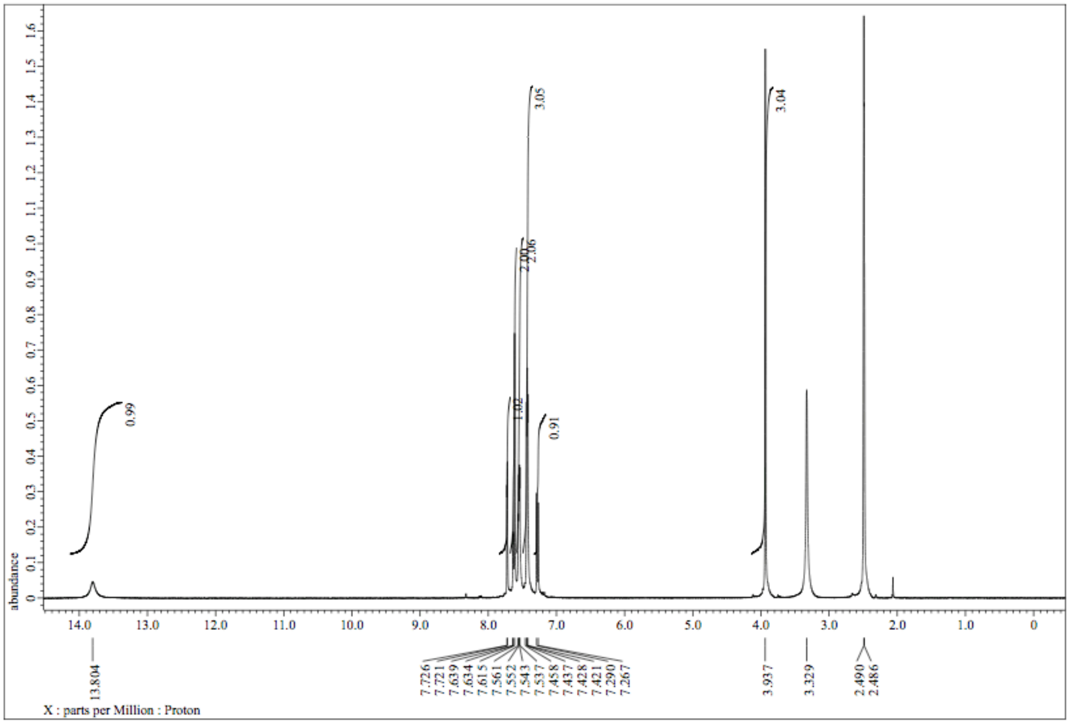

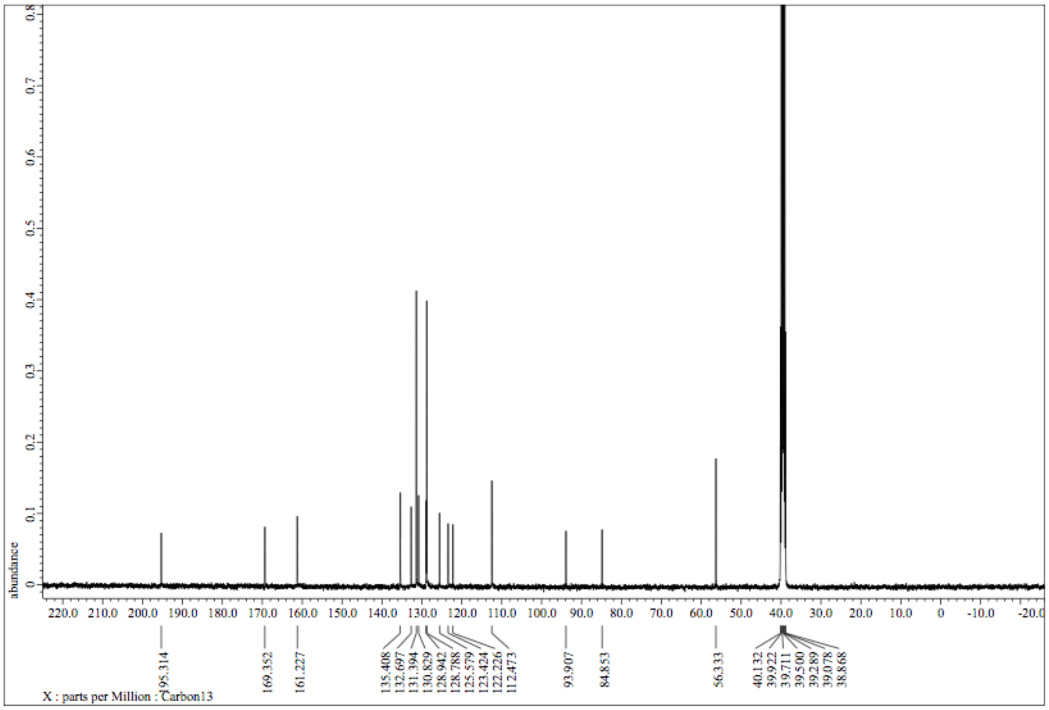

^{1}H NMR (400 MHz) and ^{13}C NMR (100 MHz) spectra of **RD0531** (DMSO-d_6)

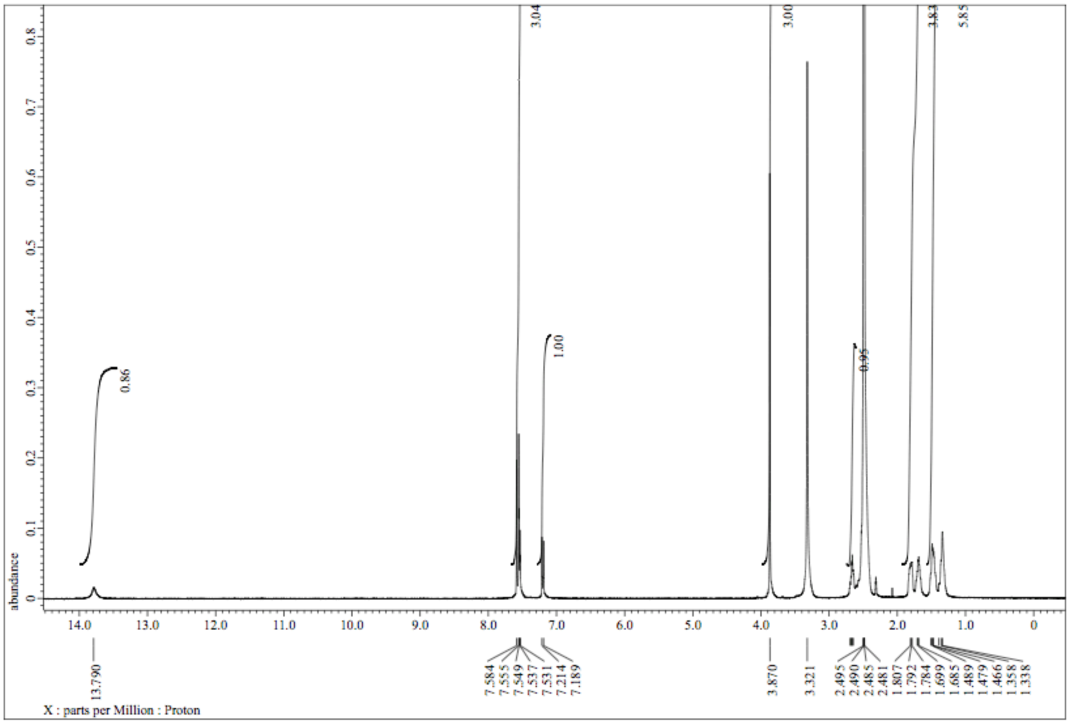

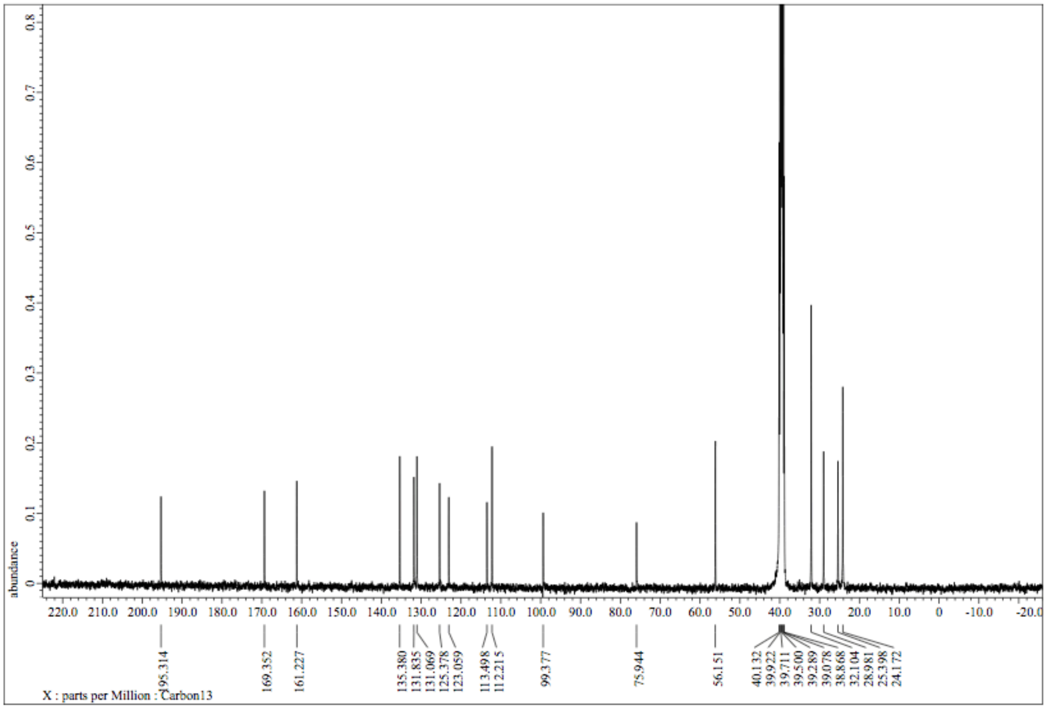

^{1}H NMR (400 MHz) and ^{13}C NMR (100 MHz) spectra of **RD0561** (DMSO-d_6)

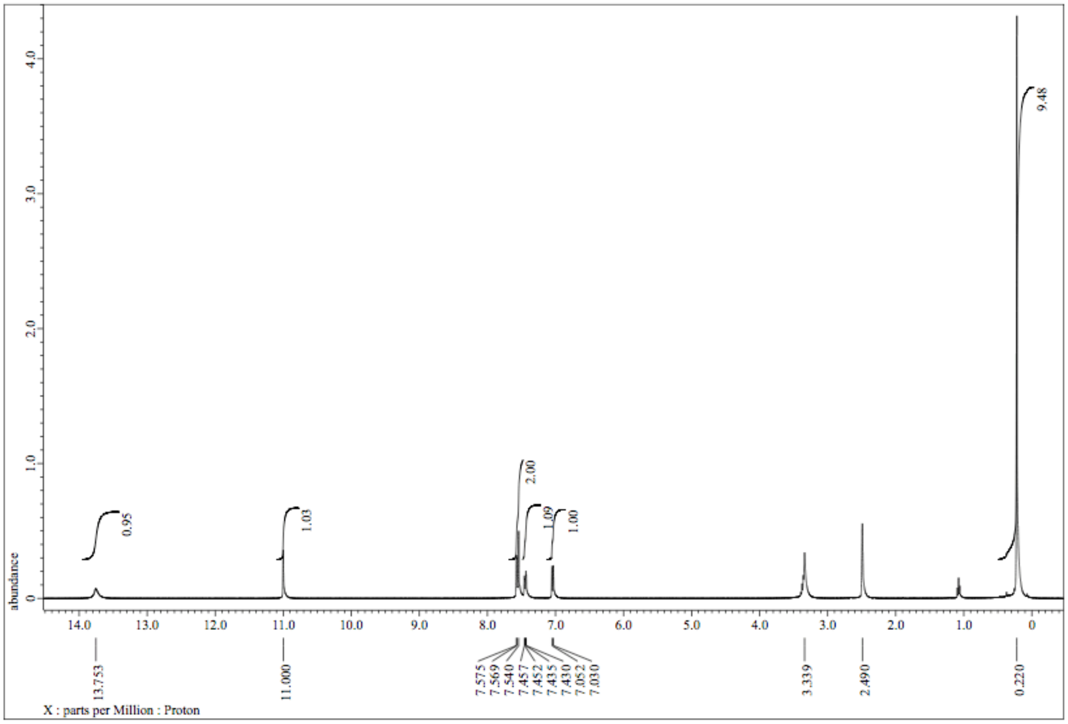

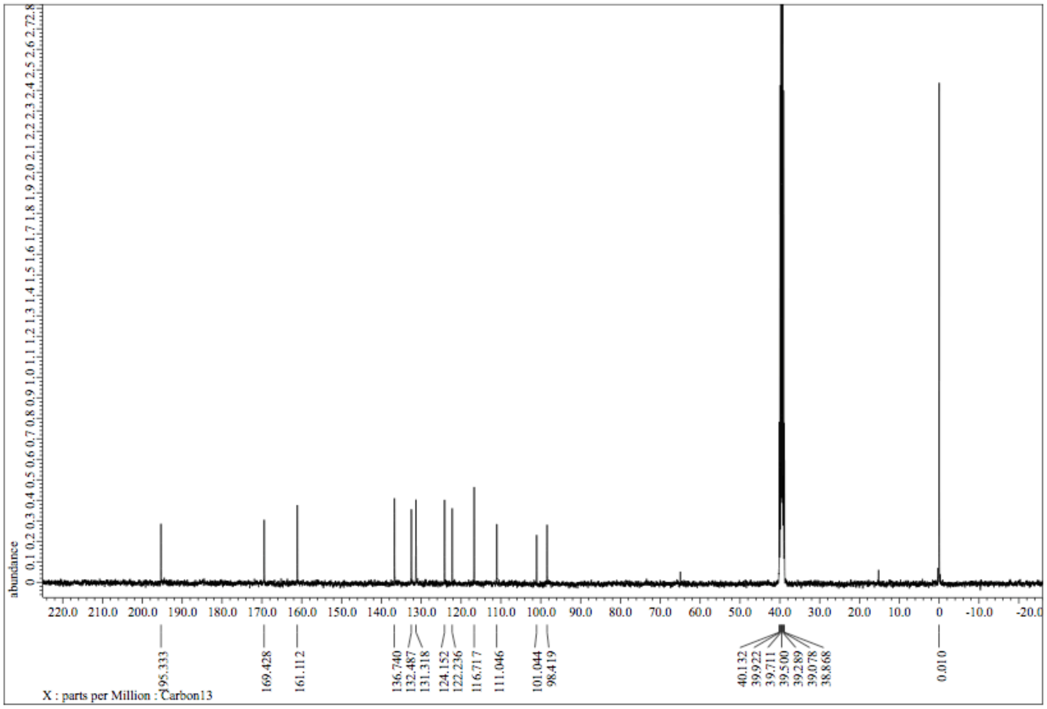

Supplementary References

1 Ogawa, Y. *et al*. Development of a novel selective inhibitor of the Down syndrome-related kinase Dyrk1A. *Nature communications* **1**, 86 (2010).
